# Mitoguardin 1 and 2 promote granulosa cell proliferation by activating AKT and regulating the Hippo-YAP1 signaling pathway

**DOI:** 10.1038/s41419-023-06312-y

**Published:** 2023-11-27

**Authors:** Ming-Qi Yan, Bing-Hong Zhu, Xiao-Hong Liu, Yu-Meng Yang, Xiu-Yun Duan, Yong Wang, Hui Sun, Mei Feng, Tao Li, Xiao-Man Liu

**Affiliations:** 1 Department of Central Laboratory, Shandong Provincial Hospital, Shandong University, 250021 Jinan, China; 2 Department of Obstetrics and Gynecology, The First Affiliated Hospital of Shandong First Medical University & Shandong Provincial Qianfoshan Hospital, 250014 Jinan, China; 3 Department of Infection Control, Jen Ching Memorial Hospital, 215300 Kunshan, China; 4 Department of Clinical Laboratory Medicine, Shandong Provincial Hospital Affiliated to Shandong First Medical University; Institute of Clinical Microbiology, Shandong Academy of Clinical Medicine, 250021 Jinan, China; 5 Department of Central Laboratory, Shandong Provincial Hospital Affiliated to Shandong First Medical University, 250021 Jinan, China; 6 Department of Obstetrics and Gynecology, Shandong Provincial Hospital Affiliated to Shandong First Medical University, 250021 Jinan, China; 7 Present Address: Department of Central Laboratory, Shandong Provincial Hospital Affiliated to Shandong First Medical University, 250021 Jinan, China

**Keywords:** Cell proliferation, Infertility, Mitochondria, HIPPO signalling

## Abstract

Mitochondria have been identified to be involved in oxidative phosphorylation, lipid metabolism, cell death, and cell proliferation. Previous studies have demonstrated that mitoguardin (Miga), a mitochondrial protein that governs mitochondrial fusion, mitochondria-endoplasmic reticulum (ER) contacts, lipid formation, and autophagy, is crucial for ovarian endocrine and follicular development. Nevertheless, whether mammalian MIGA1 or MIGA2 (MIGA1,-2) regulates ovarian granulosa cell proliferation remains unclear. This study revealed that mammalian MIGA1,-2 promotes cell proliferation and regulates the phosphorylation and localization of Yes-associated protein 1 (YAP1) in ovarian granulosa cells. MIGA2 upregulation resulted in reduced YAP1 activity, while MIGA2 removal led to increased YAP1 activity. Further analysis indicated that MIGA1,-2 regulated YAP1 via the Hippo signaling pathway and regulated protein kinase B (AKT) activity in collaboration with YAP1. In addition, lysophosphatidic acid (LPA) regulated MIGA2 expression and AKT activity by activating YAP1. Briefly, we demonstrated that the mitochondrial MIGA1 and MIGA2, especially MIGA2, promoted cellular proliferation by activating AKT and regulating the Hippo/YAP1 signaling pathway in ovarian granulosa cells, which may contribute to the molecular pathogenesis of reproductive endocrine diseases, such as polycystic ovary syndrome (PCOS).

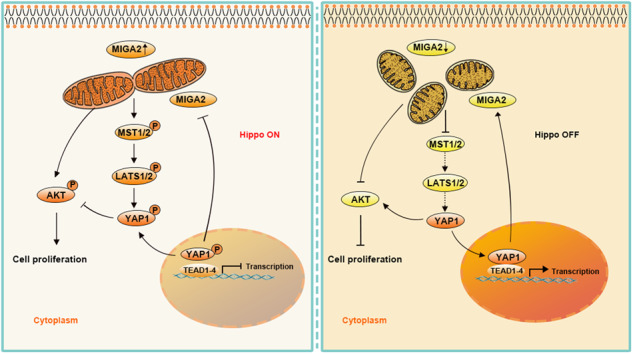

## Introduction

During follicular development, granulosa cells (GCs) undergo a transition from a predominantly proliferative to a highly differentiated state [1, 2]. Disruptions in GC proliferation can cause ovarian dysfunction and various diseases, including polycystic ovarian syndrome (PCOS) and ovarian cancer (OC) [3, 4]. In patients with diminished ovarian reserve, mitochondrial dysfunction has been identified as contributing to impaired cell proliferation and GC apoptosis [5]. However, the underlying regulatory mechanisms remain unclear.

Mitochondria serve as energy generators and signaling organelles, playing pivotal roles in maintaining redox homeostasis, calcium homeostasis, and apoptosis [6]. Along with physiological activities, the mitochondrial network undergoes constant dynamic remodeling via fusion and fission [7]. Studies have demonstrated that mitochondrial dynamics govern mitochondrial morphology and mass, influencing mitochondrial functions like respiration activity, reactive oxygen species (ROS) production, and mtDNA stability [8, 9]. Mitofusin 2 (MFN2) promotes mitochondrial fusion, decreases ROS production, and impedes cell proliferation in ovarian cancer cells [10]. Dynamin-related protein 1 (DRP1) upregulation induces mitochondrial fragmentation and elevates ROS production, altering cellular bioenergy and inhibiting OC progression [11]. These findings suggested that disordered mitochondrial dynamics may be crucial in OC progression, leading to abnormal GC proliferation.

Recent research has demonstrated the critical role of mitochondrial dynamics in cell quality control and proliferation. Damage to mitochondrial dynamics can negatively affect bioenergy supply and result in the generation of ROS, ultimately hindering cell proliferation [12]. Gonadotropin-induced mitochondrial elongation and network formation suggested that mitochondrial fusion is significantly induced during GC growth and differentiation [13]. However, the role of mitochondrial fusion in GC proliferation and the exact regulatory mechanism remain unclear.

The indispensable role of Yes-associated protein 1 (YAP1) in cellular proliferation is governed by the Hippo pathway [14]. YAP1 is sequestered in the cytoplasm after phosphorylation on Ser (127), curtailing its function as a transcription factor [15–17]. The delicate control of YAP1 activity in GCs guarantees ovarian follicle development by regulating cell proliferation and differentiation [18]. High expression of YAP1 has been reported as a prognostic marker for OC progression [19]. YAP1 downregulation inhibits GC proliferation and induces cell apoptosis, partially due to mitochondrial malfunction [5]. YAP1 has been identified as a regulator of the phosphoinositide 3-kinase (PI3K)/Protein kinase B (AKT) signaling during cardiomyocyte proliferation [20]. AKT is also crucial for cell proliferation, and its activation through phosphorylation on Ser 473 (S473) and Thr 308 (T308) also enhances YAP1 activity in ovarian GCs [21, 22]. Ovarian fragmentation has been demonstrated to activate the PI3K/AKT pathway, leading to the nuclear translocation of YAP1 [23].

Previous studies have demonstrated that the mitochondrial proteins mitoguardin 1 (MIGA1) and mitoguardin 2 (MIGA2) facilitate outer mitochondrial membrane (OMM) fusion [24]. Furthermore, MIGA2 regulates PI3K activity and autophagy through autophagy-related protein 14 (ATG14) [25]. MIGA1 or MIGA2 (MIGA1,-2) expression has been implicated in hyperandrogenism in patients with PCOS [26]. Additionally, *YAP1* has been identified as a susceptibility gene for PCOS and is regulated by androgens [27, 28]. This study examined whether human MIGA1,-2 regulates cell proliferation in GCs through the PI3K/AKT or the Hippo/YAP1 pathway. A *Miga1 and Miga2* (*Miga1/2*) double knockout mouse model and a human granulosa tumor cell line (KGN) were applied to reveal the essential roles of MIGA1,-2 in GC proliferation. Importantly, we uncovered that MIGA2 is crucial in regulating YAP1 localization and activity in ovarian GCs, which might be a promising target for breaking through the follicular development disorder in patients with PCOS.

## Materials and methods

### Animals

Wild-type (WT) mice and *Miga1/2* knockout mice were obtained as previously described [24]. Mice were maintained under a 12 h light-dark cycle with ad libitum access to regular food and water. Female mice at postnatal days (PD) 21–23 were injected with 5 international units (IU) pregnant mare serum gonadotropin (PMSG) intraperitoneally (Ningbo Sansheng Pharmaceutical Co., ZJ, China) for 44 h, followed by the injecting of 5 IU human chorionic gonadotropin (hCG) for 48 h (Ningbo Sansheng Pharmaceutical Co., ZJ, China) [27]. At least six mice in each group were randomly selected and their ovaries were obtained for assay. All animal experiments were conducted according to the guidelines of the Animal Research Committee of Shandong Provincial Hospital affiliated to Shandong First Medical University, following recommendations outlined in the guidelines for the Care and Use of Laboratory Animals of the Animal Research Committee of Shandong Provincial Hospital affiliated to Shandong First Medical University.

### Cell culture

The human GC line KGN (RIKEN BioResource Center, IBR, Japan) was cultured in DMEM/F-12 medium (HyClone, UT, USA) supplemented with 10% fetal bovine serum (FBS) (Biological Industries, USA) and antibiotics (100 IU/mL penicillin and 100 μg/mL streptomycin, Gibco, NY, USA). Similarly, HeLa cells (National Collection of Authenticated Cell Cultures, SH, China) were cultured in DMEM medium (Gibco, NY, USA) supplemented with 10% FBS and antibiotics (100 IU/mL penicillin, 100 μg/mL streptomycin). Cells were incubated under a humid environment at 37 °C and 5% CO_2_. Treatment of cells with a combination of forskolin (FSK; 10 mM) and phorbol 12-myristate 13-acetate (PMA; 20 nM) (Sigma, SL, USA) for 24 h can significantly induce cyclic adenosine monophosphate (cAMP) production and luteinization of GCs.

### DNA constructs and lentivirus production

Construct of *pCMV-flag YAP1 5SA*, which can consistently express active YAP1, was a gift from Kunliang Guan (Addgene plasmid # 27371; http://n2t.net/addgene:27371; RRID:Addgene_27371) [29]. And the *p2×Flag CMV2-YAP1-S127D* mutant, which can consistently express inactivated YAP1, was a gift from Marius Sudol (Addgene plasmid # 19051; http://n2t.net/addgene:19051; RRID:Addgene_19051) [30]. The human MIGA1,-2 cDNA sequences were labeled with a FLAG tag (DYKDDDDK) and cloned into the corresponding lentiviral vectors as previously reported [26]. The GFP-expressing lentivirus was used as a control during infection. Transfection efficiency was confirmed by detecting the mRNA or protein expression of target genes using quantitative real-time polymerase chain reaction (qRT-PCR) and western blotting.

### qRT-PCR

Total RNA was extracted using TRIzol Reagent (Invitrogen, CA, USA) and used as a template for reverse transcription to obtain cDNAs using Prime Script RT reagent Kit with gDNA Eraser (TaKaRa, SHG, Japan). Each cDNA sample was repeated three times in an independent experiment, and each experiment was repeated at least three times. The qRT-PCR was performed on a LightCycler 480 II Real-Time PCR instrument (Roche, BY, Germany) using the SYBR^®^ Green PCR Master Mix (TaKaRa, SHG, Japan). Relative mRNA expression of genes was calculated using the comparative crossing points (Cp) method and the formula 2^−^^∆∆^^Cp^. *GAPDH* was used as the reference gene [31]. The relative expression of mRNA is presented as fold change. Primer sequences are listed in Supplementary Table S1.

### Western blotting (WB)

Proteins were separated using sodium dodecyl sulfate-polyacrylamide gel electrophoresis (SDS-PAGE), transferred to the polyvinylidene difluoride (PVDF) membranes and blocked in 5% skim milk. The membranes were then incubated with primary antibodies at 4 °C overnight, followed by incubation with horseradish peroxidase (HRP)-conjugated secondary antibodies. Table 1 lists the antibodies used in this study. The blots were developed using chemiluminescent HRP Substrates (Millipore, MA, USA) in a GelDoc2 XR Gel Documentation System (BioRad, CA, USA). Each experiment was repeated at least three times. The band intensity was analyzed using the ImageJ software. Full and uncropped western blot images have been uploaded in Supplemental Materials.

**Table 1:** Antibodies applied in this study.

Antibodies	RRID	Source company	Catalog number	Working concentration	Description
FLAG	AB_10950495	Cell Signaling Technology	8146	WB: 1:1000	Mouse monoclonal
HA	AB_10691311	Cell Signaling Technology	2367	WB: 1:1000	Mouse monoclonal
pAKT (S473)	AB_2315049	Cell Signaling Technology	4060	WB: 1:1000	Rabbit monoclonal
AKT	AB_915783	Cell Signaling Technology	4691	WB: 1:1000	Rabbit monoclonal
PCNA	AB_2160330	Proteintech	10205-2-AP	WB: 1:1000	Rabbit polyclonal
pYAP1 (S127)	AB_2650553	Cell Signaling Technology	13008	WB: 1:1000	Rabbit monoclonal
YAP1	AB_2650491	Cell Signaling Technology	14074	WB: 1:1000; IF: 1:200; IHC: 1:100	Rabbit monoclonal
pMST1 (Thr183)/MST2 (Thr180)	AB_2799355	Cell Signaling Technology	49332	WB: 1:1000	Rabbit monoclonal
MST1	AB_2144632	Cell Signaling Technology	3682	WB: 1:1000; IHC: 1:200	Rabbit polyclonal
STK3 antibody (MST2)	AB_2198801	Proteintech	12097-1-AP	WB: 1:1000; IHC: 1:200	Rabbit polyclonal
pLATS1 (Thr1079)	AB_10971635	Cell Signaling Technology	8654	WB: 1:1000	Rabbit monoclonal
LATS1	AB_2133513	Cell Signaling Technology	3477	WB: 1:1000	Rabbit monoclonal
LATS2	AB_10835233	Cell Signaling Technology	5888	WB: 1:1000	Rabbit monoclonal
MIGA2 (FAM73B)	AB_11129174	Abcam	ab122713	WB: 1:1000; IHC: 1:200; IP: 1:100	Rabbit polyclonal
MFN2	AB_2266320	ProteinTech	12186-1-AP	WB: 1:1000	Rabbit polyclonal
TOMM20	AB_945896	Abcam	ab56783	IF: 1:2000	Mouse monoclonal
GAPDH	AB_2107436	ProteinTech	60004-1-Ig	WB: 1:1000	Mouse monoclonal
β-ACTIN	AB_2223172	Cell Signaling Technology	4970	WB: 1:1000	Rabbit monoclonal

### Cell viability

Cell viability was measured using the cell counting kit-8 (CCK-8, Beyotime Biotechnology Co., Ltd., SH, China) according to the manufacturer’s instructions. Briefly, 1 × 10^4^ cells/well were dispensed in aliquots and seeded in a 96-well plate. Absorbance was measured at 450 nm after different treatments of the cells using a Multiskan Go microplate reader (BioTeK, WA, USA). Each experiment included triplicate wells from the same group and was independently repeated three times.

### EdU assay

EdU assay was performed using EdU assay kit (RIBOBIOCo., Ltd., GZ, China) according to the manufacturer’s instructions. Briefly, the KGN cells were seeded in 96-well plates (100 μL per well) at a density of 1 × 10^4^ cells/mL. After different treatments, EdU was added to the cell culture medium 2 h before the assay and then was analyzed using the kit, and images were captured in the ImageXpress Micro Confocal device (Molecular Devices, SH, China). The number of EdU-positive cells was counted using ImageJ software. The proportion of EdU-positive cells was calculated, counting at least 500 cells per sample.

### Mitochondrial morphology observation

Mitochondrial morphology was observed by staining with Mitotracker Red (Molecular Probes, Invitrogen, USA) at the concentration of 250 nM for 30 min at 37 °C in dark. Cells were co-stained with Hoechst33342 and were observed with a confocal microscope (SP8, Leica, HE, Germany). Mitochondrial morphology was analyzed according to four major types and the cells were counted using ImageJ software. The proportion of cells with each mitochondrial type was calculated, counting at least 200 cells per sample.

### Mitochondrial membrane potential (MMP)

MMP was detected by staining with the fluorescent probe JC-1 (5 μM in DMEM/F-12; Invitrogen, CA, USA) for 30 min at 37 °C in dark. For confocal microscopy images, the cells were seeded on glassware for confocal, stained with JC-1 and Hoechst33342, and imaged under a confocal microscope (SP8, Leica, HE, Germany). Flow cytometry was used to quantify the MMP. Cells were suspended and then stained with JC-1, washed with PBS, and 1 × 10^4^ cells per sample were harvested and analyzed using flow cytometry (Becton, Dickinson & Company, CA, USA). MMP was calculated as the proportion of the intensity of red fluorescence to green fluorescence after staining of JC-1.

### Immunofluorescence (IF)

The cells were fixed in 4% paraformaldehyde (PFA) for 30 min, permeabilized with 0.1% Triton X-100 in PBS for 10 min, and blocked in 5% bovine serum albumin (BSA) for 30 min The cells were then incubated overnight with primary antibodies at 4 °C. After washing three times, the cells were incubated with secondary antibodies conjugated to a fluorescent dye for 30 min at room temperature in the dark. The cells were counterstained with DAPI and imaged under a Leica TCS SP8 confocal microscope (Leica, HE, Germany). Antibody information is listed in Table 1.

### Immunohistochemistry (IHC)

Mouse ovaries were fixed in 4% PFA, embedded in paraffin, and sectioned. IHC staining was performed as described before [32]. Ovarian sections were incubated with primary antibodies against YAP1, pYAP1, and MST1/2 (Abcam, Cambridge, UK), followed by incubation with biotin-labeled secondary antibodies (VECTASTAIN ABC kit, Vector Laboratories, CA, USA). Staining was performed using 3, 3 diaminobenzidine peroxidase substrate (DAB, Vector Laboratories, CA, USA). The sections were counterstained with hematoxylin. Images were captured using the TissueFAXS Plus system (TissueGnostics, WIE, Austria).

### Luminescence assay

Assays were performed in 96-well plates using the Dual-Luciferase® Reporter assay system kit (Promega Corporation, WI, USA) according to the manufacturer’s instructions. The luminescence was detected using an LB 960 microplate luminometer (Berthold Technologies, BW, Germany). The firefly luciferase activity of YAP1 was normalized to the Renilla luciferase activity. The data are presented as fold change compared to the non-treatment/non-transfection group.

### Statistical analysis

All data are presented as mean ± standard deviation. Comparisons between the two groups were done using the Student’s *t*-test, and multiple comparisons were done using one-way ANOVA. All experiments were repeated at least three times. The intra-assay variation coefficients were less than 10%, and the inter-assay variation coefficients were less than 15%. Differences were considered statistically significant when *P < 0.05 and **P < 0.01.

## Results

### MIGA1 and 2 promote ovarian GC proliferation

**Fig. 1:**
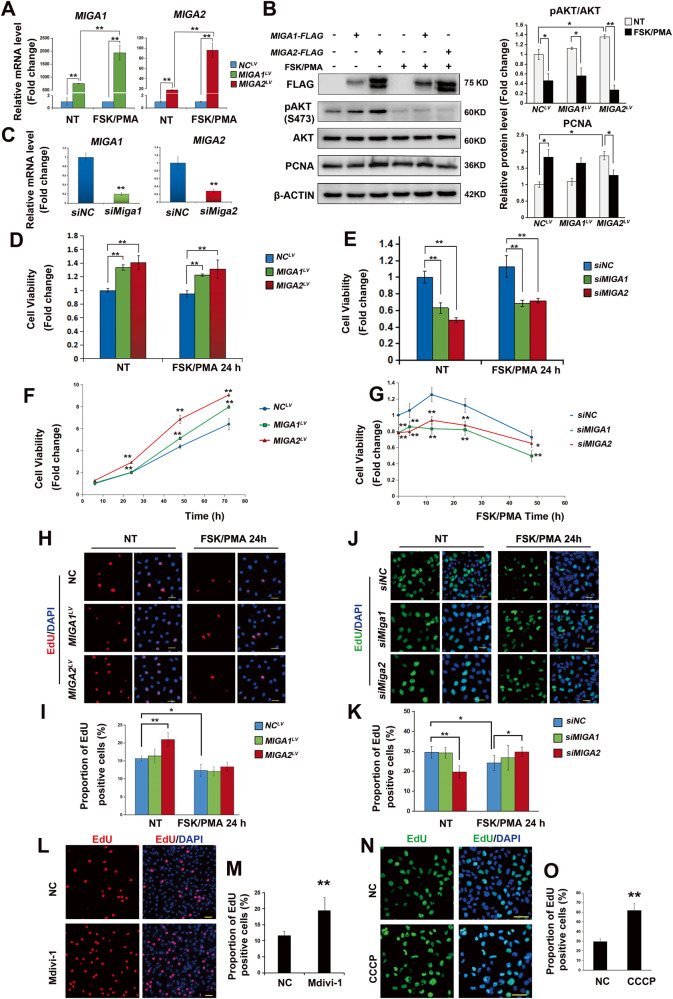
MIGA1,-2 regulates AKT activity and KGN cell proliferation. **A** Relative mRNA expression of MIGA1,-2 in *MIGA1-* or MIGA*2-*overexpressing KGN cells. Cells were treated with or without FSK/PMA for 24 h to induce cAMP production and luteinization. NT untreated, NC negative control, LV lentivirus. **B** Western blotting analysis of pAKT (S473), AKT, and PCNA in *MIGA1-* or MIGA*2-*overexpressing cells treated with or without FSK/PMA. **C** Identification of the knockdown efficiency of *MIGA1* or *-2* on mRNA levels. **D**, **E** Cell viability in KGN cells after overexpression of *MIGA1* or *-2* (**D**), or knockdown of *MIGA1* or *-2* for 48 h (**E**) with or without FSK/PMA treatment. **F**, **G** Cell viability after overexpression of *MIGA1* or *-2* for different times as indicated from 6–72 h (**F**), or after knockdown of *MIGA1* or *-2* and treatment with FSK/PMA for different times from 0 to 48 h (**G**). **H**, **I** Representative images of EdU assay in *MIGA1-* or MIGA*2-*overexpressing cells with or without FSK/PMA treatment. Scale bars, 50 μm (**H**), and the statistical analysis (**I**). **J**, **K** Representative images of EdU assay after *MIGA1* or *-2* knockdown for 48 h with or without FSK/PMA treatment for 24 h. Scale bars, 50 μm (**J**), and their statistical analysis (**K**). **L**, **M** Representative images of EdU assay for Mdivi-1 treatment at 25 μM for 24 h in KGN cells. Scale bars, 50 μm (**L**), and the statistical analysis data (**M**). **N**, **O** Representative images of EdU assay for CCCP treatment at 10 μM for 24 h. Scale bars, 50 μm (**N**). Statistical analysis of the proportion of EdU-positive cells in (**O**). Data were presented as mean ± SD. *, *P* < 0.05, **, *P* < 0.01.

To determine whether MIGA1,-2 were involved in human GC proliferation, KGN cells were modified to stably overexpress *MIGA1*,-*2*, or knockdown *MIGA1*,-*2*. Overexpression or knockdown efficiency was confirmed at the mRNA and protein levels (Fig. 1A–C). FSK/PMA treatment for 24 h significantly increased *MIGA1*,*-2* gene expression (Fig. 1A, B), while *MIGA2* overexpressing cells showed elevated levels of pAKT (S473), suggesting the enhancement of AKT activity, while FSK/PMA-induced luteinization reduced AKT activity in either group (Fig. 1B). Besides, the proliferating cell nuclear factor (PCNA) was increased after *MIGA2* overexpression but decreased after FSK/PMA treatment compared to the untreated cells (Fig. 1B). Overexpression of *MIGA1*,-*2* significantly increased cell viability in a time-dependent manner compared to the negative control even after 24 h of FSK/PMA treatment (Fig. 1D, F). In contrast, the knockdown of *MIGA1*,-*2* significantly decreased cell viability compared to the negative control, even when cells were treated with FSK/PMA at different time points compared to the negative control (Fig. 1E, G).

Cell proliferation was further examined using EdU assay to detect nuclear proliferative activity. Overexpression of *MIGA2* rather than *MIGA1* significantly increased the proportion of EdU-positive cells, suggesting that *MIGA2* may play a more critical role in cell proliferation. However, FSK/PMA treatment eliminated this difference and resulted in an overall decrease in the proportion of EdU-positive cells compared to untreated cells (Fig. 1H, I). Likewise, the knockdown of *MIGA2* rather than *MIGA1* significantly reduced the proportion of EdU-positive cells (Fig. 1J, K). Interestingly, MIGA2 knockdown increased the percentage of EdU-positive cells after 24 h of FSK/PMA treatment. To test whether *MIGA2* regulates cell proliferation through mitochondrial dynamic changes, KGN cells were treated with mitochondrial division inhibitor-1 (Mdivi-1). Results revealed that inhibiting mitochondrial division by Mdivi-1 promoted mitochondrial aggregation and significantly increased the proportion of EdU-positive KGN cells (Fig. 1L, M, Supplementary Fig. S1A, B), similar to the results of *MIGA2* overexpression. This indicates that MIGA2 may promote cell proliferation by promoting mitochondrial fusion in KGN cells.

Furthermore, carbonyl cyanide 3-chlorophenylhydrazone (CCCP), a mitochondrial oxidative phosphorylation uncoupling agent, has been used to confirm the pivotal function of mitochondrial activity in cell proliferation. The proportion of EdU-positive cells was significantly increased after CCCP treatment for 24 h in KGN cells (Fig. 1N, O). The mitochondrial activity was further measured by detecting MMP in KGN cells, and the results showed that knocking down *MIGA2* significantly reduced MMP (Supplementary Fig. S1C–E). All these results suggest that MIGA1,-2 promotes cell proliferation, whereas MIGA2 exhibits a stronger effect, possibly by regulating mitochondrial fusion and function.

### MIGA1 and 2 regulate YAP1 phosphorylation and localization

**Fig. 2:**
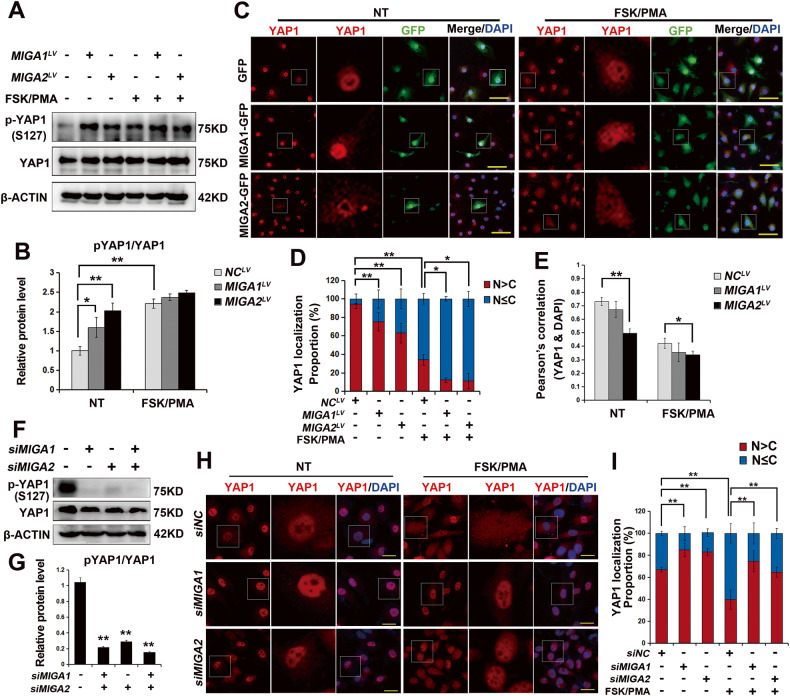
MIGA1,-2 regulates YAP1 phosphorylation and localization in KGN cells. **A**, **B** Representative western blotting images of pYAP1 (S127) and YAP1 in *MIGA1-* or MIGA*2-*overexpressing KGN cells with or without FSK/PMA treatment for 24 h (**A**), and the quantitative analysis of pYAP1(S127)/YAP1 ratio (**B**). NT untreated, NC negative control. **C** Representative immunofluorescence images showing YAP1 localization in GFP-tagged *MIGA1-* or *MIGA2-*overexpressing cells treated with or without FSK/PMA. Scale bars, 50 μm. **D** The statistical analysis of YAP1 localization for data from (**C**). N > C, predominantly nuclear; N ≤ C, predominantly cytoplasm or even distribution in nucleus and cytoplasm. **E** Pearson’s correlation analysis between YAP1 and DAPI for data from (**C**). **F**, **G** Representative western blotting images of pYAP1 (S127) and YAP1 proteins after knockdown of *MIGA1* or *-2* for 48 h (**F**), and pYAP1(S127)/YAP1 ratio was calculated (**G**). **H** Representative immunofluorescence images of YAP1 localization in *MIGA1* or *-2* knockdown cells with or without FSK/PMA treatment for 24 h. Scale bars, 50 μm. **I** The statistical analysis of YAP1 localization for (**H**). N > C, predominantly nuclear; N ≤ C, predominantly cytoplasm or even distribution in nucleus and cytoplasm. Data were presented as mean ± SD. *, *P* < 0.05, **, *P* < 0.01.

Since the Hippo/YAP1 pathway has been implicated in regulating GC proliferation and differentiation during ovarian follicle development. Therefore, the involvement of YAP1 was evaluated in MIGA1- or MIGA2-regulated cell proliferation in KGN cells. The results disclosed that overexpression of MIGA1,-2 increased YAP1 phosphorylation (pYAP1) at S127, and luteinization induced by FSK/PMA increased the levels of pYAP1 (S127) compared to the untreated cells, whereas the increase in pYAP1 (S127) by MIGA1,-2 overexpression was slightly attenuated by luteinization (Fig. 2A, B). Additionally, overexpression of MIGA1,-2 changed the subcellular localization of YAP1, shifting it predominantly from the nucleus to the cytoplasm, especially after 24 h of FSK/PMA treatment. The Pearson’s correlation coefficient between YAP1 and the nucleus decreased in MIGA2 overexpressing cells after 24 h of FSK/PMA treatment (Fig. 2C–E). In contrast, the knockdown of MIGA1,-2 resulted in a significant decrease in the phosphorylation of YAP1 at S127 (Fig. 2F, G) and an increase in the distribution of YAP1 in the nucleus. However, FSK/PMA treatment decreased the proportion of YAP1 localized in the nucleus, while knockdown of MIGA1,-2 increased the proportion of YAP1 localized in the nucleus compared with the negative control (Fig. 2H, I).

It was further observed that CCCP promoted YAP1 localization to the nucleus but also promoted YAP1 out of the nucleus after 24 h of FSK/PMA treatment (Supplementary Fig. S2A, B). Furthermore, Mdivi-1 inhibited YAP1 from entering the nucleus and enhanced YAP1 phosphorylation at S127 (Supplementary Fig. S2C–E). These results suggest that MIGA1- or MIGA2-mediated mitochondrial function regulates YAP1 phosphorylation at S127 and its localization in GCs.

### MIGA2 regulates YAP1 activity in ovarian GCs

**Fig. 3:**
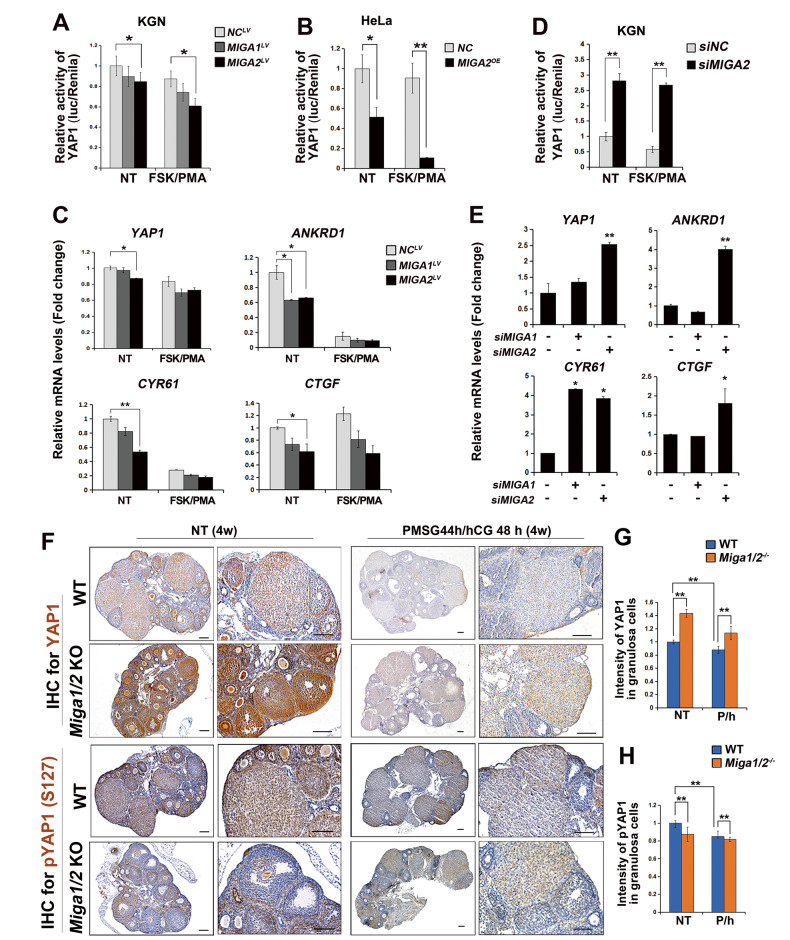
MIGA2 inhibits YAP1 activity in ovarian granulosa cells. **A**, **B** Luciferase analysis of the transcriptional activity of *YAP1* in KGN cells after overexpression of *MIGA1* or *-2* (**A**) and in Hela cells after overexpression of *MIGA2* (**B**) with or without FSK/PMA treatment. NT untreated, NC negative control. **C** Relative mRNA expression of *YAP1* and its target genes of *ANKRD1*, *CYR61*, and *CTGF* in KGN cells stably overexpressing *MIGA1* or *-2* with or without FSK/PMA treatment. **D** Luciferase analysis of the transcriptional activity of *YAP1* after knockdown of *MIGA2* in KGN cells treated with or without FSK/PMA. **E** Relative mRNA expression of *YAP1* and its targeting genes of *ANKRD1*, *CYR61*, and *CTGF* after knockdown of *MIGA2* in KGN cells. **F**–**H** Representative images of immunohistochemistry staining of YAP1 and pYAP1 (S127) in *Miga1/2* double knockout and wild-type (WT) mouse ovaries. The mice at the age of 4 weeks were either treated with PMSG for 44 h and followed by hCG for 48 h or not. Scale bars, 100 μm (**F**), and relative intensity of immunohistochemical staining for YAP1 (**G**) and pYAP1 (S127) (**H**) in ovarian granulosa cells. P/h, PMSG 44 h/hCG 48 h. Data were presented as mean ± SD. *, *P* < 0.05, **, *P* < 0.01.

The impact of MIGA2 on the transcriptional activity of YAP1 was examined in KGN cells. The results showed a decrease in YAP1 transcriptional activity after *MIGA2* overexpression with FSK/PMA treatment or not (Fig. 3A), which was further validated in HeLa cells (Fig. 3B), revealing that MIGA2 significantly reduced the transcriptional activity of YAP1 with or without FSK/PMA treatment. Compared with the control group, the mRNA expression of *YAP1* and its target genes *ANKRD1*, *CYR61* and *CTGF* were downregulated in *MIGA2*-overexpressing cells (Fig. 3C). Other YAP1 targeting genes, *AMOTL2* and *IGFBP3*, did not significantly change in *MIGA1* or *-2* overexpressing cells (Supplementary Fig. S3A). In contrast, when *MIGA2* was knocked down, the transcriptional activity of *YAP1* in KGN cells significantly increased, even after 24 h of FSK/PMA treatment (Fig. 3D). Consistently, the mRNA expression of *YAP1* and its target genes, *ANKRD1*, *CYR61* and *CTGF* increased after *MIGA2* knockdown (Fig. 3E). Similarly, CCCP treatment resulted in increased mRNA expression of *YAP1*, *ANKRD1* and *CYR61* (Supplementary Fig. S3B). Surprisingly, the mRNA expression changes of *AMOTL2* and *IGFBP* in *MIGA2* knockdown cells exhibited different patterns after FSK/PMA treatment, where *AMOTL2* was significantly decreased after treatment with FSK/PMA for 2 h or 24 h, whereas *IGFBP3* was increased significantly (Supplementary Fig. S3C). IHC staining for YAP1 and pYAP1 (S127) was performed on mouse ovarian sections. The results presented that compared with the WT group, YAP1 expression was enhanced in the follicular GCs of *Miga1/2* knockout mice (Fig. 3F, G), while pYAP1 (S127) expression was reduced (Fig. 3F, H). Similar results were observed in mouse luteal cells after stimulating luteinization with PMSG 44h/hCG 48 h in vivo (Fig. 3F–H). YAP1 expression in follicular GCs was also verified by immunofluorescence staining (Supplementary Fig. S3D). These results suggest that MIGA1 and MIGA2, especially the latter, regulate YAP1 transcriptional activity and its target gene expression in ovarian GCs.

### MIGA2 regulates YAP1 and TEAD1-4 via the Hippo pathway in GCs

**Fig. 4:**
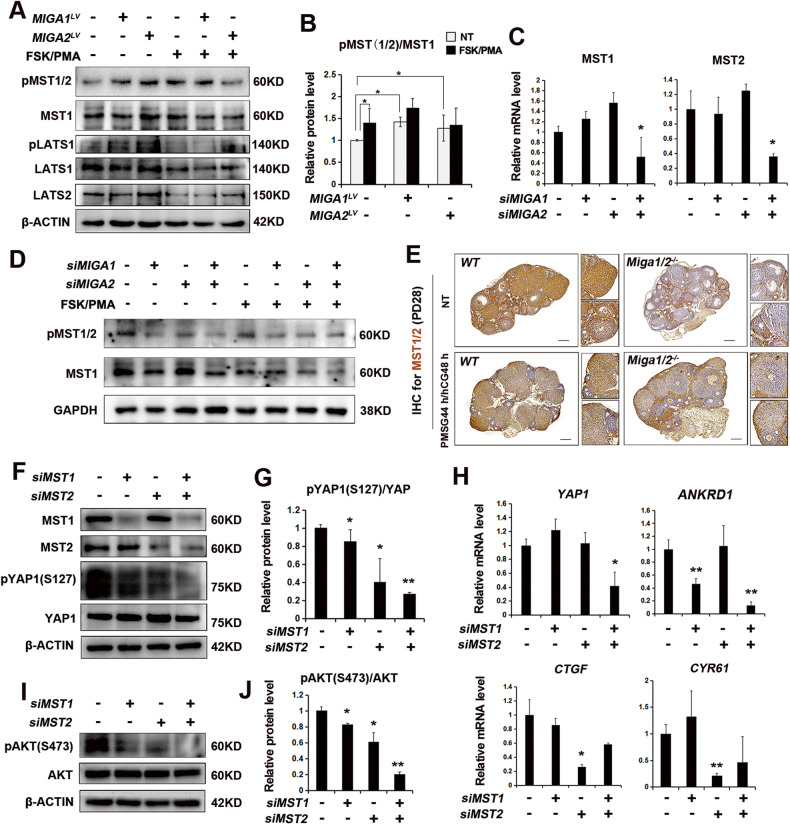
MIGA1,-2 regulates YAP1 activity via the modulation of the Hippo pathway. **A**, **B** Western blotting analysis of pMST1/2, MST1, pLATS1, LATS1, and LATS2 proteins after overexpression of *MIGA1 or -2* and with the treatment of FSK/PMA or not in KGN cells (**A**), and the quantitative analysis of pMST(1/2)/MST1 ratio. NT, untreated (**B**). **C** Relative mRNA expression of *MST1* and *MST2* after knockdown of *MIGA1* or -*2* in KGN cells. **D** Western blotting images of pMST1/2 and MST1 after knockdown of *MIGA1* or *-2* and with FSK/PMA treatment or not for 24 h in KGN cells. **E** Representative images of immunohistochemistry staining of MST1/2 in *Miga1/2* double knockout and wild-type (WT) mouse ovaries. Mice aged 4 weeks were either injected with PMSG 44 h/hCG 48 h or untreated (NT). Scale bars, 200 μm. **F**, **G** Western blotting images of MST1, MST2, pYAP1 (S127), and YAP1 proteins after knockdown of *MST1* or *MST2* in KGN cells (**F**), and the quantitative analysis of pYAP1(S127)/YAP1 ratio (**G**). **H** Relative mRNA expression of *YAP1*, *ANKRD1*, *CTGF*, and *CYR61* after transfection of *siMST1* or *siMST2* for 48 h in HeLa cells. **I**, **J** Western blotting images of pAKT (S473) and AKT proteins after knockdown of *MST1* or *MST2* in KGN cells (**I**), and the quantitative analysis of pAKT(S473)/AKT ratio (**J**). Data were presented as mean ± SD. *, *P* < 0.05, **, *P* < 0.01.

The findings that MIGA1,-2 regulates YAP1 phosphorylation at S127 suggest the possible activation of the Hippo pathway by *MIGA1*,*-2*. Therefore, changes in key kinases in the Hippo pathway were examined, and increased phosphorylation of MST1/2 (T183 of MST1 and T180 of MST2) and LATS1 (T1079) was found in *MIGA1* or *-2* overexpressing KGN cells, indicating the activation of the Hippo pathway. However, FSK/PMA treatment only increased pMST1/2 but not pLATS1 levels in KGN cells (Fig. 4A, B). Consistently, the knockdown of *MIGA1* and *-2* resulted in a significant decrease in the mRNA transcriptional expression of *MST1* and *MST2* in KGN cells (Fig. 4C). Protein levels of pMST1/2 and MST1 were also determined, the results showed that the ratio of pMST(1/2)/MST1 was almost unchanged after *MIGA1* or *-2* knockdown in KGN cells, however, the total protein levels of MST1 were significantly decreased (Fig. 4D, Supplementary Fig. S4A), indicating the inactivation of Hippo signaling pathway. The expression of MST1/2 in the ovaries of *Miga1/2* knockout mice was further examined using IHC assays. It was found that MST1/2 protein expression was significantly reduced in follicular GCs of *Miga1/2* knockout mice with or without PMSG 44h/hCG 48 h treatment, whereas it was not reduced in stromal cells (Fig. 4E, Supplementary Fig. S4B). To confirm the role of MST1/2 in YAP1 activity in GCs, we knocked down *MST1* and *MST2* in KGN cells (Fig. 4F, Supplementary Fig. S4C, D). Knockdown of either *MST1* or *MST2* significantly inhibited YAP1 phosphorylation at S127 compared to the control, and double knockdown had a superimposed effect in reducing YAP1 phosphorylation (Fig. 4F, G). Besides, the mRNA levels of the *YAP1* gene were significantly reduced after knockdown of *MST1* and *MST2*, and the YAP1 target gene *ANKRD1* was significantly reduced in *MST1* knockdown cells or *MST1/2* double knockdown cells, whereas *CTGF* and *CYR61* were decreased only in *MST2* knockdown cells (Fig. 4H). Furthermore, the knockdown of either *MST1* or *MST2* reduced AKT activity by reducing AKT phosphorylation at S473 (Fig. 4I, J). It was also observed that the knockdown of *MST1* and *MST2* in KGN cells significantly reduced the expression of MIGA2 and MFN2 proteins but had a lesser effect on the MFN2 protein (Supplementary Fig. S4E–G). These results suggest that MIGA1,-2 may regulate YAP1 activity by regulating the Hippo pathway and that the Hippo pathway may regulate MIGA2 expression GCs.

Since YAP1 initiates its target gene expression by binding to the nuclear transcription factors TEADs, the mRNA expression of *TEADs* was further examined in *MIGA1* or *-2* overexpression or knockdown cells. Overexpression of *MIGA1* or *-2* reduced the transcriptional levels of *TEAD1-4* in both proliferating and luteinized GCs (Supplementary Fig. S4H). Knockdown of *MIGA2* increased *TEAD1-4* mRNA expression, and concurrent knockdown of YAP1 and *MIGA2* increased the expression of *TEAD2*, *3*, and *4* compared to the knockdown of *YAP1* (Supplementary Fig. S4I), suggesting that both YAP1 and TEADs are involved in the MIGA2-mediated proliferation of GCs.

### MIGA2 and YAP1 synergistically regulate AKT activity

**Fig. 5:**
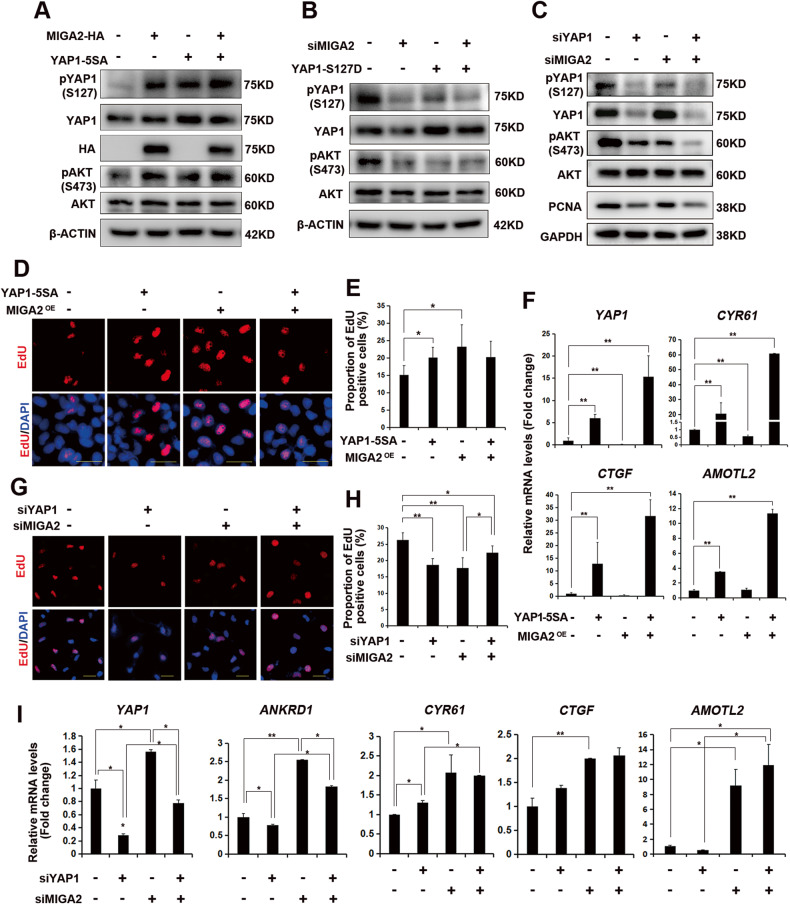
MIGA2 and YAP1 synergistically regulate AKT phosphorylation. **A** Representative western blotting images of pYAP1 (S127), YAP1, pAKT (S473), AKT proteins after overexpression of HA-tagged *MIGA2* or *YAP1-5SA* in HeLa cells. **B** Representative western blot images of pYAP1 (S127), YAP1, pAKT (S473), and AKT proteins after transfection of *MIGA2* siRNA or *YAP1-S127D* plasmid. **C** Representative western blot images of pYAP1 (S127), YAP1, pAKT (S473), AKT, and PCNA proteins in KGN cells transfected with *siMIGA2* or *siYAP1*. **D**, **E** Representative images of EdU assay after overexpression of *YAP1-5SA* or *MIGA2-HA* in HeLa cells. Scale bars, 25 μm (**D**), and statistical analysis of the proportion of EdU-positive cells (**E**). **F** Relative mRNA expression of *YAP1* and *YAP1* targeted *CYR61*, *CTGF*, and *AMOTL2* genes after overexpression of *YAP1-5SA* or *MIGA2-HA* in HeLa cells. **G**, **H** Representative images of EdU assay after transfection of siRNAs for *MIGA2* or *YAP1* in KGN cells. Scale bars, 25 μm (**G**), and the statistical analysis of the proportion of EdU-positive cells (**H**). **I** Relative mRNA expression of *YAP1*, *ANKRD1*, *CYR61*, *CTGF*, and *AMOTL2* in KGN cells transfected with *siMIGA2* or *siYAP1*. Data were presented as mean ± SD. *, *P* < 0.05, **, *P* < 0.01.

To determine whether MIGA2 and YAP1 regulate cell proliferation through PI3K/AKT signaling, changes in AKT activity were examined after the regulation of *MIGA2* and *YAP1* in HeLa cells. Overexpression of either *MIGA2* or *YAP1-5SA* resulted in increased phosphorylation of YAP1 at S127 and AKT at S473 (Fig. 5A, Supplementary Fig. S5A). In contrast, knockdown of *MIGA2* or transfection of the *YAP1-S127D* dominant-negative mutant reduced phosphorylation of YAP1 at S127 and AKT at S473 in Hela cells (Fig. 5B, Supplementary Fig. S5B). Furthermore, the knockdown of *MIGA2* or *YAP1* also decreased AKT phosphorylation at S473, and the knockdown of both genes resulted in a superimposed downregulation effect in KGN cells (Fig. 5C, Supplementary Fig. S5C). These results suggest that MIGA2 and YAP1 could regulate AKT activity and exert a superimposed effect on AKT activity.

We then tested the proliferative activity in *MIGA2-* or *YAP1-5SA*-overexpressing cells and found either *MIGA2* or *YAP1-5SA* significantly increased the proportion of EdU-positive cells, suggesting a high proliferative rate. However, after co-transfection of *MIGA2* and *YAP1-5SA*, the increase in cell proliferation became insignificant (Fig. 5D, E). However, the mRNA expression of *YAP1* and YAP1 target genes *CYR61*, *CTGF*, and *AMOTL2* were significantly increased (Fig. 5F). Interestingly, overexpression of *MIGA2* alone did not induce the expression of YAP1 target gene transcription. However, co-transfection with *YAP1* caused an extra enhancement, suggesting that MIGA2 and YAP1 may exert synergistic effects upon overexpression. Conversely, knocking down *MIGA2* or *YAP1* revealed a significant reduction in the proportion of EdU-positive cells, whereas double knockdown resulted in an increased proportion of EdU-positive cells compared with single *MIGA2* knockdown (Fig. 5G, H). Knockdown of *YAP1* significantly decreased *YAP1* mRNA expression, whereas knockdown of *MIGA2* increased the mRNA expression of *YAP1*. Furthermore, the concurrent knockdown of *MIGA2* and *YAP1* further increased *YAP1* mRNA expression compared to the knockdown of *YAP1* alone. Similarly, the expression patterns of *ANKRD1*, *CYR61*, and *AMOTL2* resembled *YAP1* (Fig. 5I). These results suggest that MIGA2 and YAP1 synergistically play important roles in regulating AKT activity and cell proliferation.

MIGA2 can regulate YAP1 expression, but whether MIGA2 expression is regulated by YAP1 remains unknown. Overexpression of *MIGA2* or *YAP1-5SA* increased *MIGA2* mRNA transcription levels, whereas co-expression of *YAP1-5SA* and *MIGA2* resulted in a decreased *MIGA2* mRNA level than *YAP1-5SA* overexpression alone (Supplementary Fig. S5D). Decreased protein levels of MIGA2 were detected after *MIGA2* co-expression with *YAP1-5SA* or *YAP1-S127D*, compared to *MIGA2* overexpression alone (Supplementary Fig. S5E, F). In addition, MIGA2 protein levels were decreased after the knockdown of *YAP1* or *MIGA2* and further decreased when both genes were knocked down (Supplementary Fig. S5G, H). These data suggest that MIGA2 and YAP1 regulate and restrict the expression of each other.

### Lysophosphatidic acid (LPA) regulates AKT activity and GC proliferation

**Fig. 6:**
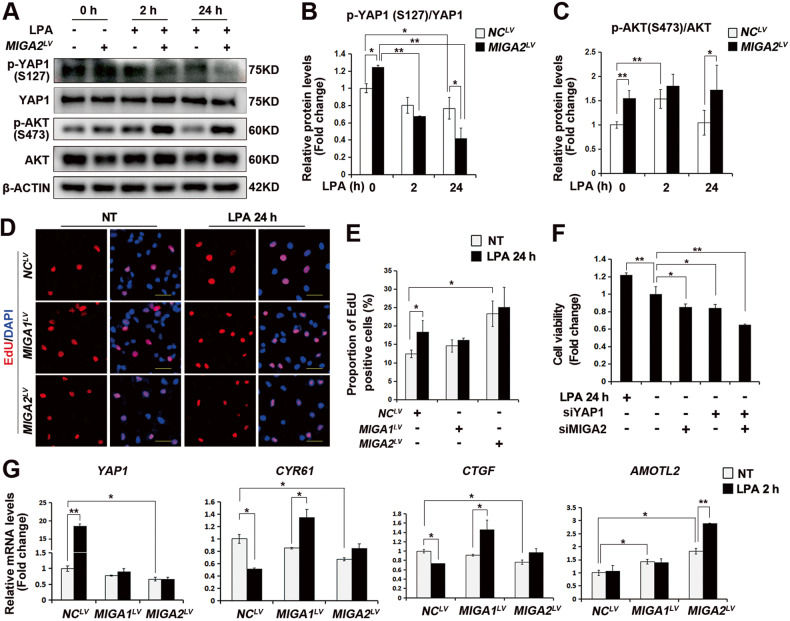
LPA regulates AKT activity and cell proliferation. **A**–**C** Representative western blot images of pYAP1 (S127), YAP1, pAKT (S473), and AKT proteins in cells stably overexpressing *MIGA2* or not and treated with LPA (25 μM) for 0 h, 2 h or 24 h (**A**), the quantitative analysis of pYAP1/YAP1 ratio (**B**) and pAKT (S473)/AKT ratio (**C**). NC, negative control. **D**, **E** Representative images of EdU assay in cells stably overexpressing *MIGA1* or *-2* and treated with LPA for 24 h. Scale bars, 25 μm (**D**), and the statistical analysis of the proportion of EdU-positive cells (**E**). NT, untreated. **F** Cell viability of KGN cells was analyzed after treatment with LPA for 24 h knockdown of *YAP1* or *MIGA2* for 48 h. **G** Relative mRNA expression of *YAP1*, *CYR61*, *CTGF*, and *AMOTL2* after overexpression of *MIGA1* or *-2* treated with LPA for 2 h or not. Data were presented as mean ± SD. *, *P* < 0.05, **, *P* < 0.01.

To verify the involvement of YAP1 in regulating AKT activity by MIGA2 during GC proliferation, LPA was applied to activate YAP1 in KGN cells. Phosphorylation of YAP1 at S127 was significantly reduced after 24 h of LPA treatment, which was further reduced in *MIGA2-*overexpressing cells (Fig. 6A, B). Conversely, LPA treatment for 2 h, but not for 24 h, increased the AKT phosphorylation at S473 in KGN cells, which was further potentiated in *MIGA2-*overexpressing cells (Fig. 6C).

EdU assay indicated that LPA treatment for 24 h alone or overexpression of *MIGA2* significantly increased the proportion of EdU-positive cells, where LPA-induced increase was attenuated in *MIGA1* or *-2* overexpressing cells (Fig. 6D, E). Moreover, cell viability was significantly decreased after knocking down *YAP1* or *MIGA2* and further reduced after simultaneous *YAP1* and *MIGA2* knockdown compared to the control (Fig. 6F). LPA treatment for 2 h significantly increased *YAP1* mRNA expression, while insignificant induction effect was observed on *YAP1* mRNA levels in *MIGA1* or *-2* overexpressing cells. Furthermore, the mRNA expression of YAP1 target genes was differentially altered, i.e., *CYR61* and *CTGF* mRNA expression was decreased after 2 h of LPA treatment while increased in *MIGA1* overexpressing cells. In contrast, *AMOTL2* mRNA expression increased after 2 h of LPA treatment in *MIGA2* overexpressing cells (Fig. 6G). In addition, LPA treatment for 2 h increased *MIGA1* and *-2* mRNA expression in *MIGA1* or *-2* overexpressing cells (Supplementary Fig. S6A, B), and promoted YAP1 localization from the cytoplasm to the nucleus (Supplementary Fig. S6C, D). This suggests that LPA may increase cell proliferation through YAP1- and MIGA2-mediated PI3K/AKT signaling in a coordinated manner.

## Discussion

Timely expression of YAP1 is critical for follicular development. YAP1 promotes GC proliferation but inhibits GC differentiation. Inactivation or deletion of YAP1 in GCs increases cell apoptosis and prevents follicular development. However, hyperactive YAP1 inhibits GC differentiation [18, 27]. Our previous study also found an increased GC apoptosis and defects in ovulation and luteinization due to the failure of GC differentiation in *Miga1/2* double knockout mice [32]. In addition, YAP1 has been identified as a susceptibility gene for PCOS [28], and MIGA2 is associated with hyperandrogenism in PCOS [26]. Consequently, it is hypothesized that the dysfunction of YAP1 and MIGA2 may disrupt follicular development and ovulation in patients with PCOS. Recently, the PI3K activity has been proven to be regulated by Miga in *Drosophila* [25], suggesting that the PI3K/AKT pathway may play an important role in the MIGA1/2-mediated regulation of GCs. To further understand the molecular signaling mechanisms underlying the functions of MIGA1 and -2 in follicular growth and development, this study investigated the effects of MIGA1 and -2 on GCs proliferation as well as on the Hippo/YAP1 and PI3K/AKT signaling pathways, to elucidate their involvement in the molecular pathogenesis of PCOS.

Our findings demonstrate that MIGA1 and -2 promote GC proliferation by facilitating mitochondrial fusion and regulating AKT and YAP1 activities in ovarian GCs. *MIGA2* upregulation increases AKT activity but inhibits YAP1 activity, whereas MIGA2 deletion inhibits AKT activity but promotes YAP1 activity in human granulosa cells. MIGA2 upregulation increased AKT and cell proliferation activity, whereas MIGA2 downregulation reduced AKT and granulosa cell proliferation activity. This suggests that reduced AKT activity may directly inhibit GC proliferation, thereby preventing follicular growth in *Miga1/2* knockout mice. Moreover, *Miga1/2* deletion leads to increased YAP1 activity in follicular GCs, which can inhibit GC differentiation and ultimately lead to failure of ovulation and luteinization.

Patients with PCOS are characterized by multiple small follicles that fail to mature and ovulate, similar to the phenotype of YAP1 activation or Miga1/2 deletion [18, 32]. Moreover, high levels of androgens promote YAP1 activity and MIGA2 expression but inhibit MIGA2 expression in luteinized ovarian GCs [27, 32]. Therefore, defects in follicular development and ovulation in patients with PCOS may be caused by high levels of androgen-induced increased YAP1 activity and reduced MIGA2 expression. However, the molecules directly linking MIGA2 and the Hippo/YAP1 pathway remain unknown, and the in vivo evidence regarding the regulatory relationship between MIGA2 and YAP1 in humans is still lacking. Accordingly, the regulatory mechanism between MIGA2 and YAP1 in PCOS patients is worthy of further investigation.

A previous study demonstrated that *MFN2* overexpression increased YAP1 expression, whereas YAP1 deletion impaired the function of *MFN2* in response to ER stress, oxidative stress, and calcium homeostasis in inflammation-induced neuronal dysfunction [33]. In addition, the lack of mitochondrial fusion proteins, such as Marf, Opa1, or Chchd3, was found to inactivate the Hippo pathway during *Drosophila* development, suggesting a cross-talk between mitochondrial fusion and the Hippo pathway [34]. It has also been reported that inactivation of the Hippo/YAP1 pathway induces mitochondrial fission by increasing DRP1 expression during myoblast differentiation [35]. Our findings revealed that overexpression of MIGA1 and -2 activates the Hippo/YAP1 pathway and is inactivated in the absence of MIGA1 and -2 in ovarian GCs. Furthermore, sustained activation of YAP1 promotes MIGA2 expression. However, it restricts MIGA2 expression in MIGA2-overexpressing cells, whereas deletion of YAP1 further represses MIGA2 expression even in MIGA2 knockdown cells, suggesting that MIGA2 and YAP1 may coordinately regulate each other to maintain the proliferative activity of ovarian GCs, verifying the link between mitochondrial dynamics and the Hippo/YAP1 pathway. Moreover, MIGA2 regulates the expression of the *TEAD1-4* genes, the core participants of the Hippo pathway, in concert with the regulatory role of YAP1. Interestingly, *TEAD4*, but not other members of the TEAD family, was recently reported to translocate to the mitochondria and be involved in regulating mitochondrial dynamics and cellular metabolism [36], further validating the link between mitochondrial dynamics and the Hippo pathway.

In summary, this study demonstrated the circulatory regulation of MIGA2 and YAP1, which positively regulated AKT activity and ovarian GC proliferation. *MIGA2* overexpression suppressed YAP1 activity, whereas its deletion increased YAP1 activity. Our data novelly presented the role of MIGA2-mediated mitochondrial fusion in regulating the Hippo/YAP1 signaling pathway during follicular development and identified a novel regulator of YAP1 during ovarian GCs. Since MIGA2 and YAP1 are associated with hyperandrogenism in PCOS, this study may provide new clues to the molecular pathogenesis of PCOS.

### Supplementary information

Supplementary Information

Reproducibility Checklist

Original Data File

## Data Availability

The original data generated and analyzed during this study are included in this article or the supplementary files.
