# Supplementary material for: Mitoguardin 1 and 2 promote granulosa cell proliferation by activating AKT and regulating the Hippo-YAP1 signaling pathway
Source: Cell Death Dis. 2023 Nov 27;14(11):779. doi: 10.1038/s41419-023-06312-y (PMC10682431; doi:10.1038/s41419-023-06312-y)
Supplement: Supplementary file 1 — Supplementary Information [file 41419_2023_6312_MOESM1_ESM.docx]

**Mitoguardin 1 and 2 promote granulosa cell proliferation by activating AKT and regulating the Hippo-YAP1 signaling pathway**

Ming-Qi Yan^1#^, Bing-Hong Zhu^2#^, Xiao-Hong Liu^3^, Yu-Meng Yang^1^, Xiu-Yun Duan^1^, Yong Wang^4^, Hui Sun^4^, Mei Feng^1^, Tao Li^5^ & Xiao-Man Liu^1, 4*^

^1^Central Laboratory, Shandong Provincial Hospital Affiliated to Shandong First Medical University; Shandong Provincial Hospital, Shandong University, Jinan 250021, China.

^2^Department of Obstetrics and Gynecology, The First Affiliated Hospital of Shandong First Medical University & Shandong Provincial Qianfoshan Hospital, Jinan 250014, China.

^3^Department of Infection Control, Jen Ching Memorial Hospital, Kunshan 215300, China.

^4^Department of Clinical Laboratory Medicine, Shandong Provincial Hospital Affiliated to Shandong First Medical University; Institute of Clinical Microbiology, Shandong Academy of Clinical Medicine, Jinan 250021, China.

^5^Department of Obstetrics and Gynecology, Shandong Provincial Hospital Affiliated to Shandong First Medical University, Jinan 250021, China.

^#^These authors contributed equally to this work.

***Correspondence author**:

Xiao-Man Liu (ORCiD: <https://orcid.org/0000-0002-8978-5937)>

Central Laboratory, Shandong Provincial Hospital Affiliated to Shandong First Medical University; Shandong Provincial Hospital, Shandong University

E-mail: [liuxiaoman@sdfmu.edu.cn](mailto:liuxiaoman@sdfmu.edu.cn)

Tel: +86-053168776912

Fax: +86-053168776912

544 Jingsi Road, Jinan, China 250021.

**Table S1. Primers used in quantitative PCR analysis.**

| Gene symbol | Gene ID (NCBI) | Forward primer  (5’-3’) | Reverse primer  (5’-3’) | Product length |
| --- | --- | --- | --- | --- |
| *MIGA1* | 374986 | GGCTTTTTGGGTCCTAGAAATTCTC | CTGCATGAGGTCTTCAGCTAAAGTC | 141 bp |
| *MIGA2* | 84895 | AGAAGTGGGAGCAGGCACTA | CCAGCTTCTCTGCAAACTCC | 147 bp |
| *YAP1* | 10413 | TAGCCCTGCGTAGCCAGTTA | TCATGCTTAGTCCACTGTCTGT | 177 bp |
| *ANKRD1* | 27063 | AGTAGAGGAACTGGTCACTGG | TGTTTCTCGCTTTTCCACTGTT | 180 bp |
| *CYR61* | 3491 | CTTGTTGGCGTCTTCGTCG | AGCCTGGTCAAGTGGAGAAG | 115 bp |
| *CTGF* | 1490 | CAGCATGGACGTTCGTCTG | AACCACGGTTTGGTCCTTGG | 115 bp |
| *AMOTL2* | 51421 | GACATGACCAAGTGGGAGCA | GGGTGCTCTGTCTGCTGTAG | 426 bp |
| *IGFBP3* | 3486 | AGAGCACAGATACCCAGAACT | GGTGATTCAGTGTGTCTTCCATT | 93 bp |
| *MST1* | 4485 | GATGGGCACTGTCCGAGTAG | AGGTCCTCCACTGTCCAACT | 321 bp |
| *MST2* | 6788 | GGGCCCAGACCATGATTGAA | CATCATGGGGTCCAGTGCTT | 341 bp |
| *TEAD1* | 7003 | ACTTCCCTTCCCTTTCGGTTT | CGTCTTGCCTGTCCTGAGTT | 323 bp |
| *TEAD2* | 8463 | TCGGAATGAACTGATCGCCC | TGGAAACCTGGTCCTTCAACT | 140 bp |
| *TEAD3* | 7005 | ACCCTCTCAGGACATCAAGC | GCTGTACGTGTCAGGGTCTC | 214 bp |
| *TEAD4* | 7004 | CACCTGTTCGTGCACATTGG | CCGAAAGAGCAGACCTTCGT | 281 bp |
| *GAPDH* | 2597 | TCTGCTCCTCCTGTTCGACA | AAAAGCAGCCCTGGTGACC | 141 bp |

**
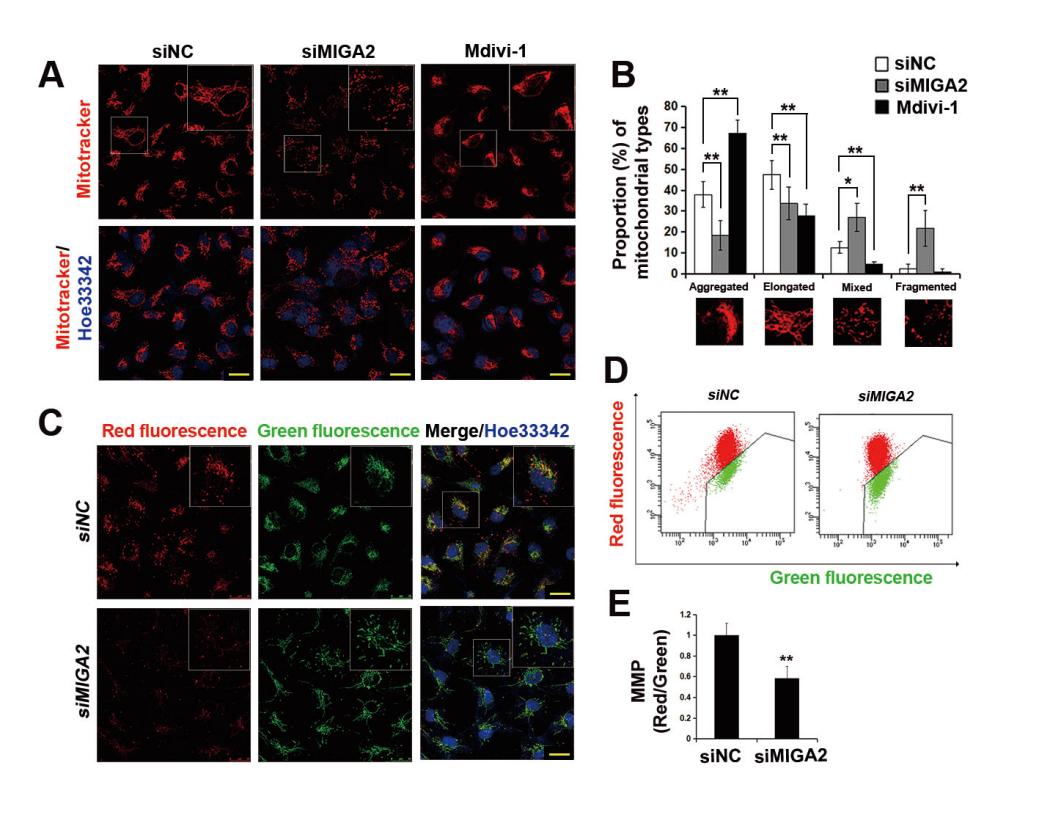
**

**Fig. S1: MIGA2 knockdown led to mitochondrial fragmentation and low MMP in KGN cells (related to Fig. 1).**

**A** Representative images of mitochondrial morphology stained by mitotracker Red (250 nM) after knockdown of MIGA2 or treatment of Mdivi-1 (25 μM) for 24 h. Hoechst33342 (Hoe33342) was used to label the nuclei. Scale bars, 25 μm. **B** Statistical analysis of the proportion of cells with the indicated mitochondrial morphology for data A. **C** Representative images of mitochondrial membrane potential (MMP) detection of cells stained with JC-1 and Hoe33342 after knockdown of MIGA2. Scale bars, 25 μM. **D** Representative flow cytometry images of MMP detection in MIGA2 knockdown cells stained with JC-1**. E** Statistical analysis of the ratio of MMP (red fluorescence/green fluorescence) for data in D. Data were presented as mean ± SD. **, *P*<*0.01*.

**
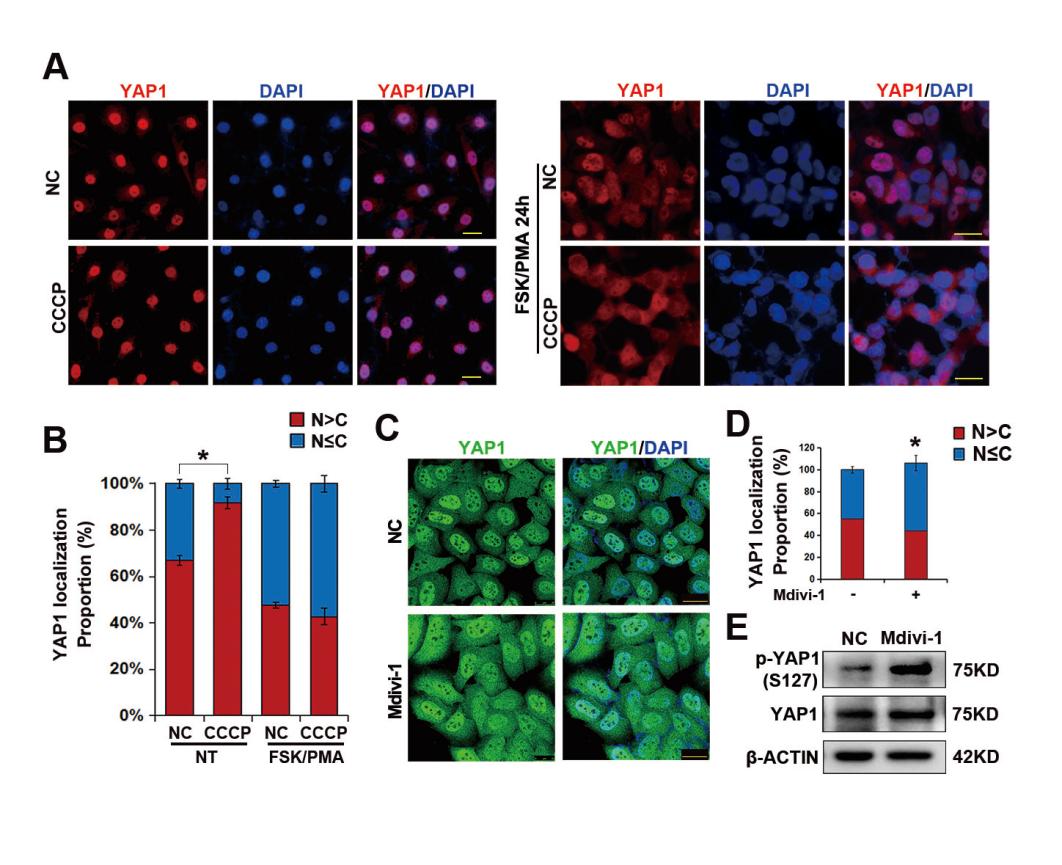
**

**Fig. S2: CCCP and Mdivi-1 regulate YAP1 phosphorylation and localization (related to main Fig. 2).**

**A** Representative immunofluorescence images of YAP1 in KGN cells treated with CCCP (10 μM) combined with FSK/PMA treatment or not for 24 h. NC, negative control. Scale bars, 25 μm. **B** Statistics analysis of the proportion of YAP1 localization. N>C, predominantly nuclear; N≤C, predominantly cytoplasm or even distribution in nucleus and cytoplasm. NT, no treatment. **C** Representative immunofluorescence images of YAP1 after Mdivi-1 (25 μM) treatment for 24 h. Scale bars, 25 μm. **D** Statistics analysis of the proportion of YAP1 localization. N>C, predominantly nuclear; N≤C, predominantly cytoplasm or even distribution in nucleus and cytoplasm. **E** Representative western blotting images of pYAP1 (S127), YAP1 and β-ACTIN proteins after 24 h of Mdivi-1 treatment. Data were presented as mean ± SD. *, *P<0.05*.


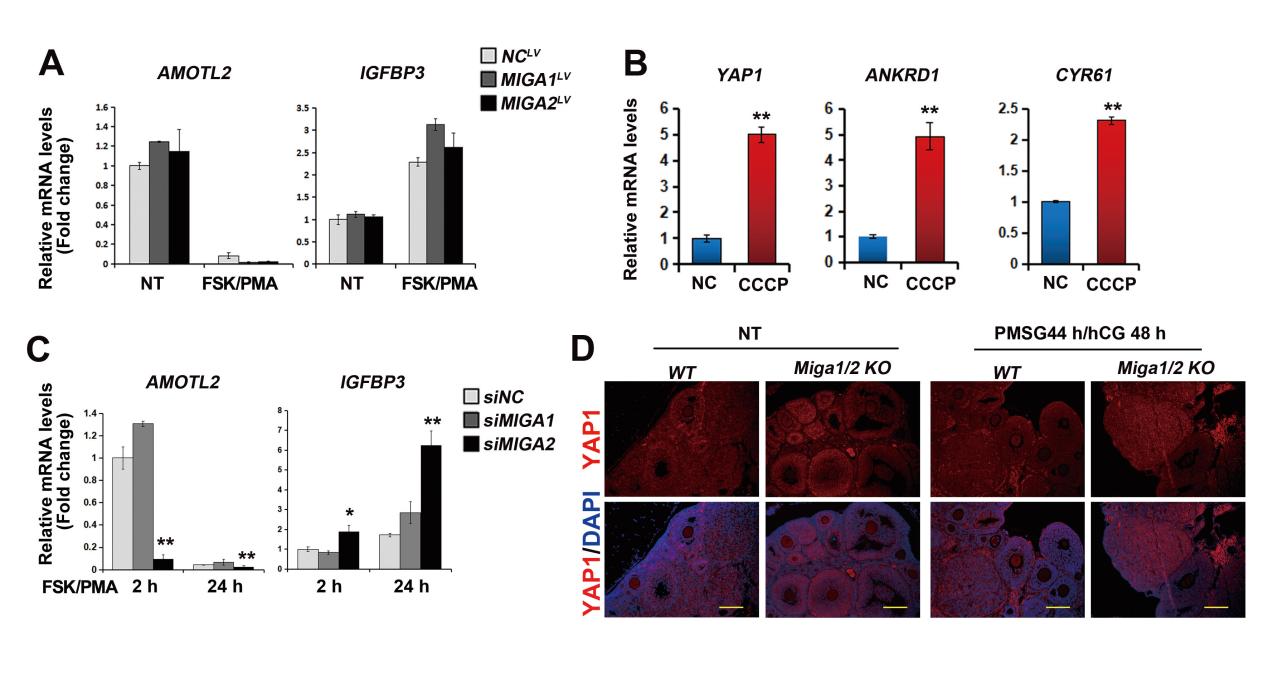


**Fig. S3: MIGA1/2 mediated mitochondrial functions regulate expression of YAP1 and YAP1 targeted genes (related to main Fig. 3).**

**A** Relative mRNA expression of *AMOTL2* and *IGFBP3* after overexpression of *MIGA1* or *MIGA2* in KGN cells with FSK/PMA treatment or not. **B** Relative mRNA expression of *YAP1*, *ANKRD1* and *CYR61* in KGN cells treated by CCCP for 24 h. **C** Relative mRNA expression of *AMOTL2* and *IGFBP3* in *MIGA1* or *MIGA2* knockdown KGN cells with FSK/PMA treatment or not. **D** Representative immunofluorescence staining images of YAP1 in *Miga1/2* knockout and wild-type (WT) mouse ovaries with no-treatment (NT) or treatment of PMSG44 h/hCG 48 h. Scale bars, 100 μm. Data were presented as mean ± SD. *, *P<0.05*, **, *P*<*0.01*.


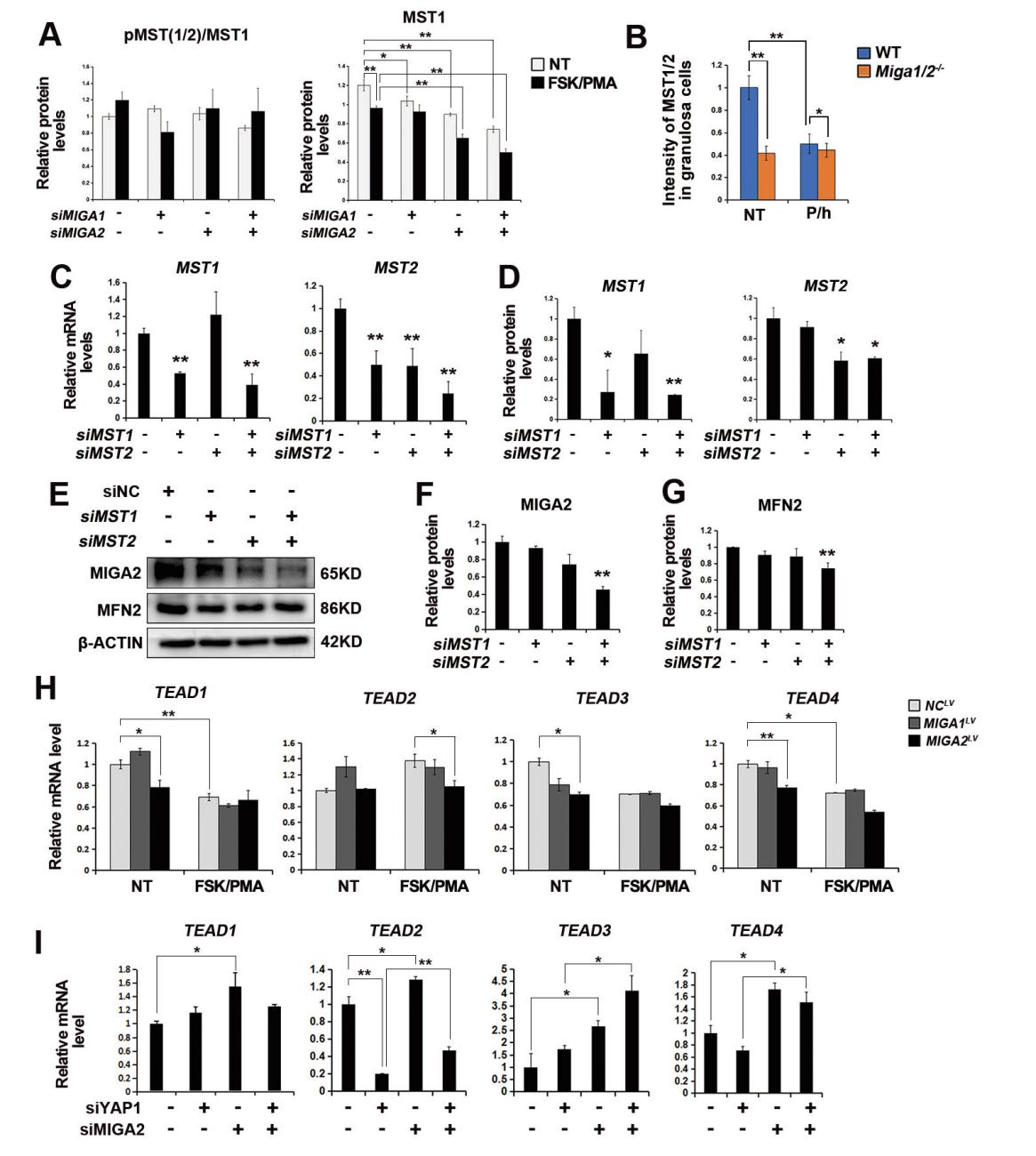


**Fig. S4: MIGA1/2 and MST1/2 modulate each other and regulate *TEAD1-4* gene expression (related to main Fig. 4).**

**A** The quantitative analysis of pMST(1/2)/MST1 ratio and MST1 protein expression for Figure 4D. NT, no treatment. **B** Relative intensity of immunohistochemical staining for MST1/2 in mouse ovarian granulosa cells for Figure 4E. WT, wildtype. **C** Relative mRNA expression of MST1 and MST2 after knockdown of MST1 or MST2 in KGN cells. **D** Relative protein expression of MST1 and MST2 after knockdown of MST1 or MST2 in KGN cells for Figure 4F. **E-G** Representative western blotting images of MIGA2 and MFN2 proteins after knockdown of *MST1* or *MST2* in KGN cells (E), and the relative quantitative analysis of the expression of MIGA2 (F) and MFN2 (G) proteins. **H&I** Relative mRNA expression of *TEAD1-4* genes in KGN cells after overexpression of *MIGA1* or *MIGA2* with FSK/PMA treatment or not. NC, negative control. (H), or after knockdown of *YAP1* or *MIGA2* (I). Data were presented as mean ± SD. *, *P<0.05*, **, *P*<*0.01*.

**
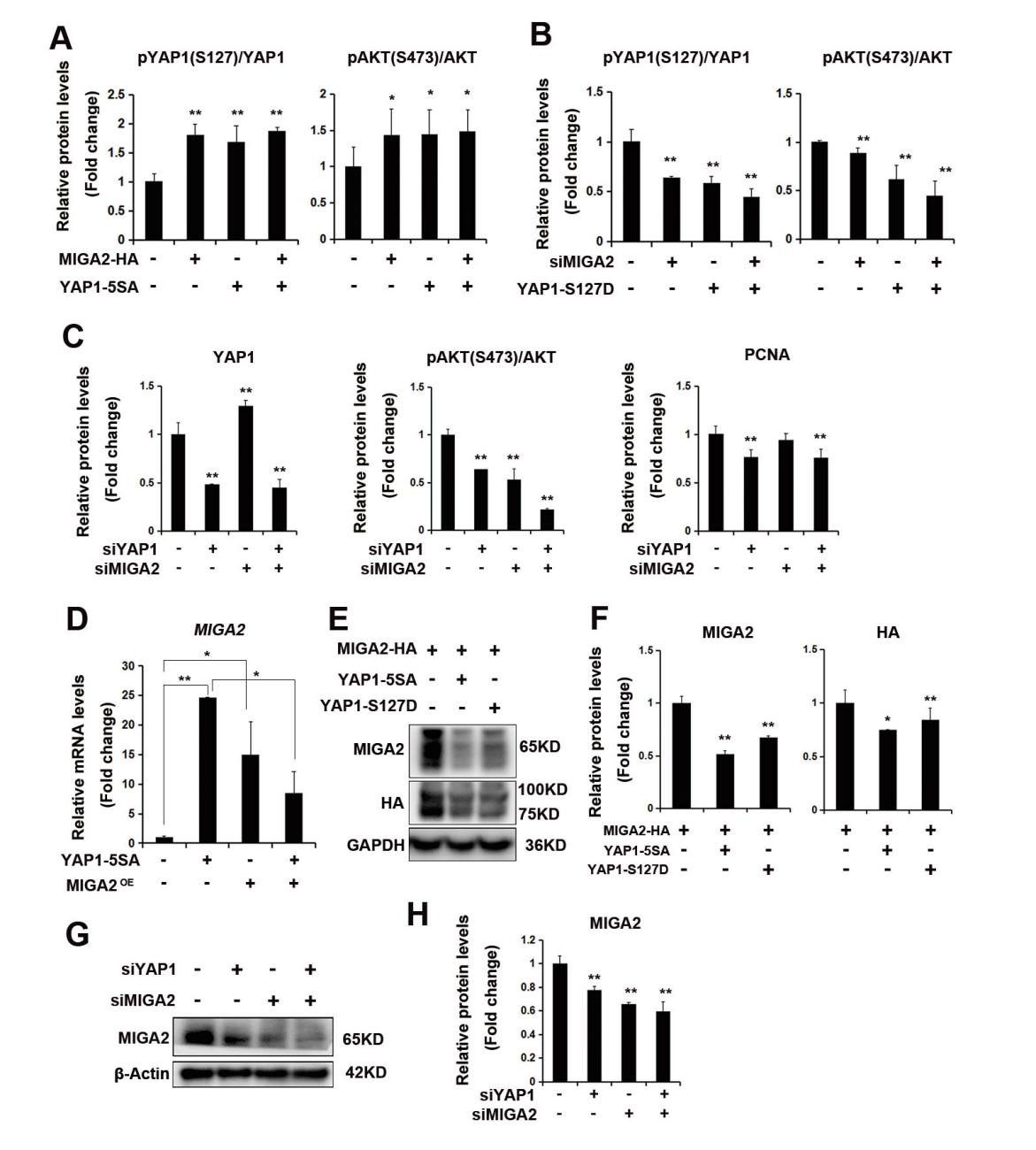
**

**Fig. S5: MIGA2 and YAP1 are regulated each other (related to main Fig. 5).**

**A&B** The pYAP1(S127)/YAP1 ratio and pAKT (S473)/AKT ratio were quantified and calculated for the levels of phosphorylated proteins for Figure 5A (A), and Figure 5B (B). **C** Quantitative analysis of YAP1 and pAKT (S473)/AKT ratio for protein levels for Figure 5C. **D** Relative mRNA expression of *MIGA2* after overexpression of *YAP1-5SA* or *MIGA2* in HeLa cells. **E&F** Representative western blotting images of MIGA2 and HA tagged MIGA2 proteins after transfection of the indicated plasmids in HeLa cells (E), and the quantitative analysis for MIGA2 and HA (F). **G&H** Representative western blotting images of MIGA2 protein after knockdown of *YAP1* or *MIGA2* in KGN cells, and the quantitative analysis for MIGA2 (H). Data were presented as mean ± SD. *, *P<0.05*, **, *P*<*0.01*.


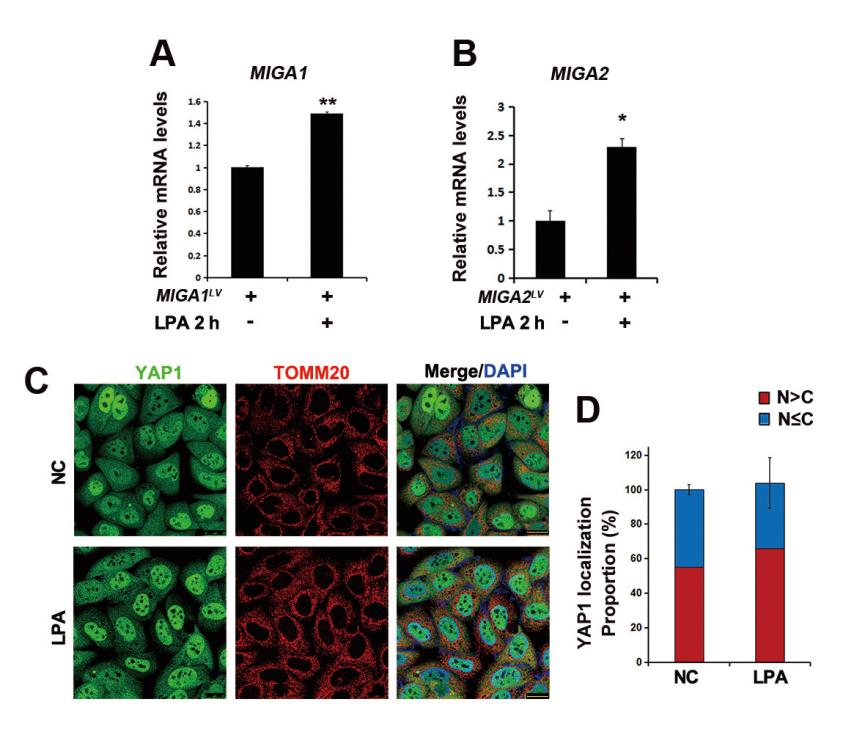


**Fig. S6: LPA regulates MIGA1 and MIGA2 expression (related to main Fig. 6).**

**A-B** Relative mRNA expression of *MIGA1* (A) and *MIGA2* (B) treated with LPA for 2 h in *MIGA1-* or *MIGA2-*overexpressing KGN cells. **C-D** Representative immunofluorescence images of YAP1 and TOMM20 after treatment of LPA for 24 h. Scale bars, 25 μm. (C), and the statistics analysis of the proportion of YAP1 localization (D). NC, negative control. N>C, predominantly nuclear; N≤C, predominantly cytoplasm or even distribution in nucleus and cytoplasm. Data were presented as mean ± SD. *, *P<0.05*, **, *P*<*0.01*.
