# Supplementary material for: Mitoguardin 1 and 2 promote granulosa cell proliferation by activating AKT and regulating the Hippo-YAP1 signaling pathway
Source: Cell Death Dis. 2023 Nov 27;14(11):779. doi: 10.1038/s41419-023-06312-y (PMC10682431; doi:10.1038/s41419-023-06312-y)

**Fig1B**

**Anti-FLAG**

**
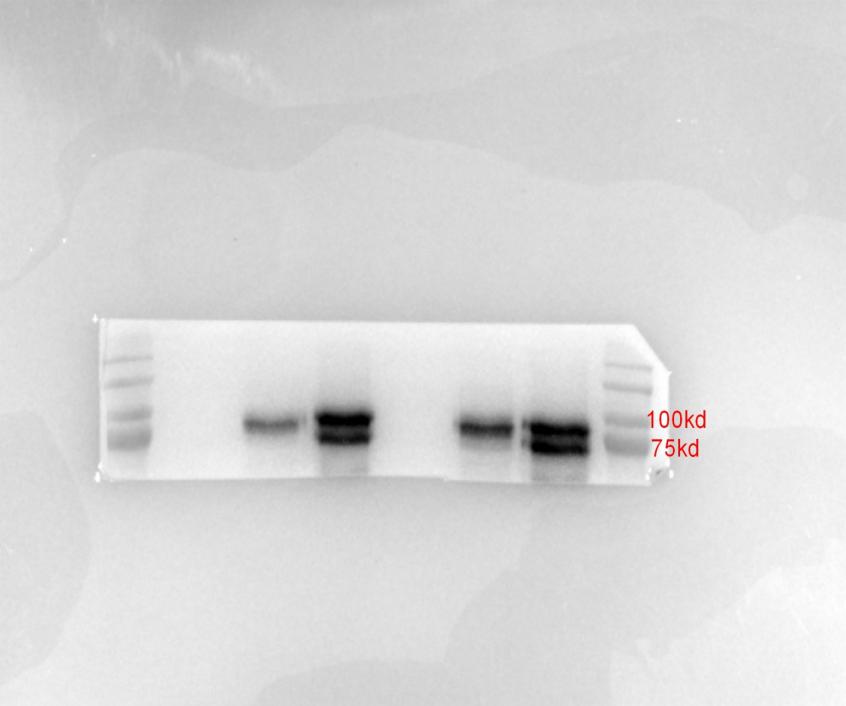
**

**pAKT (S473)**

**
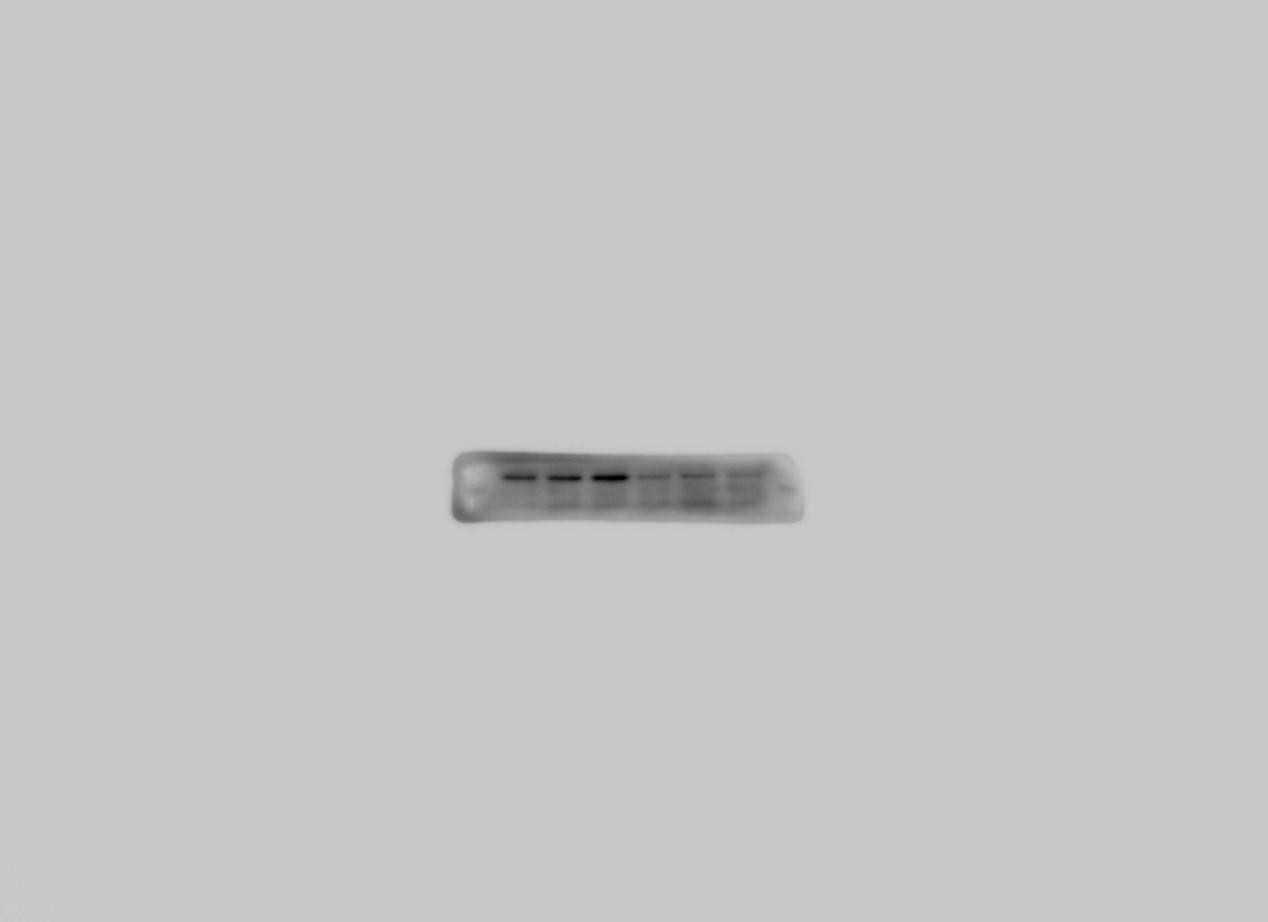
**

**AKT**

**
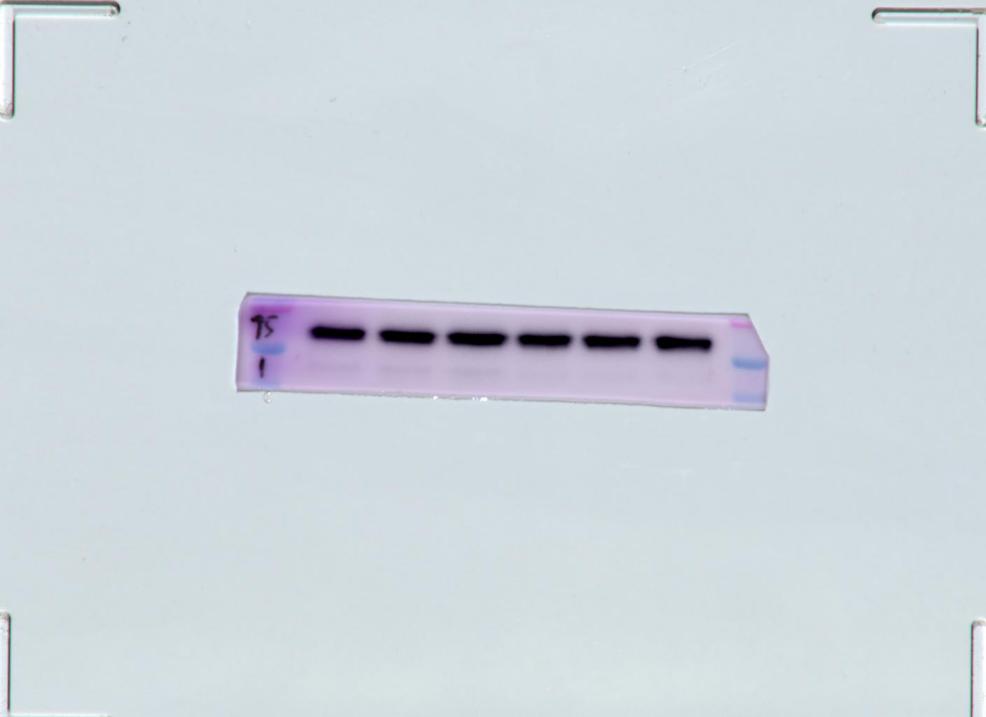
**

**PCNA**

**
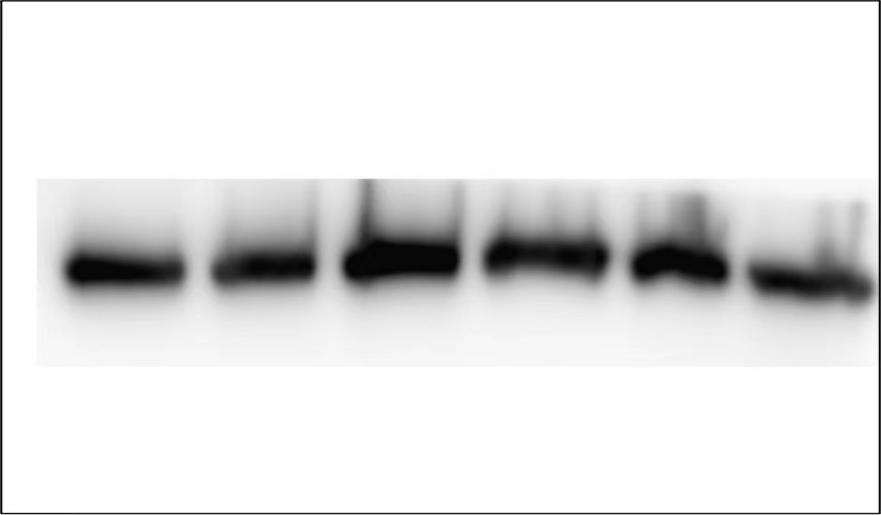
**

**ACTIN**


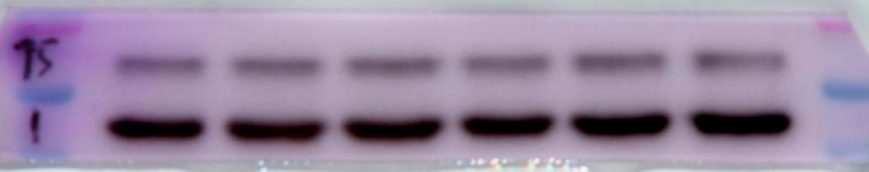


**Fig 2A**

**pYAP1(S127)**


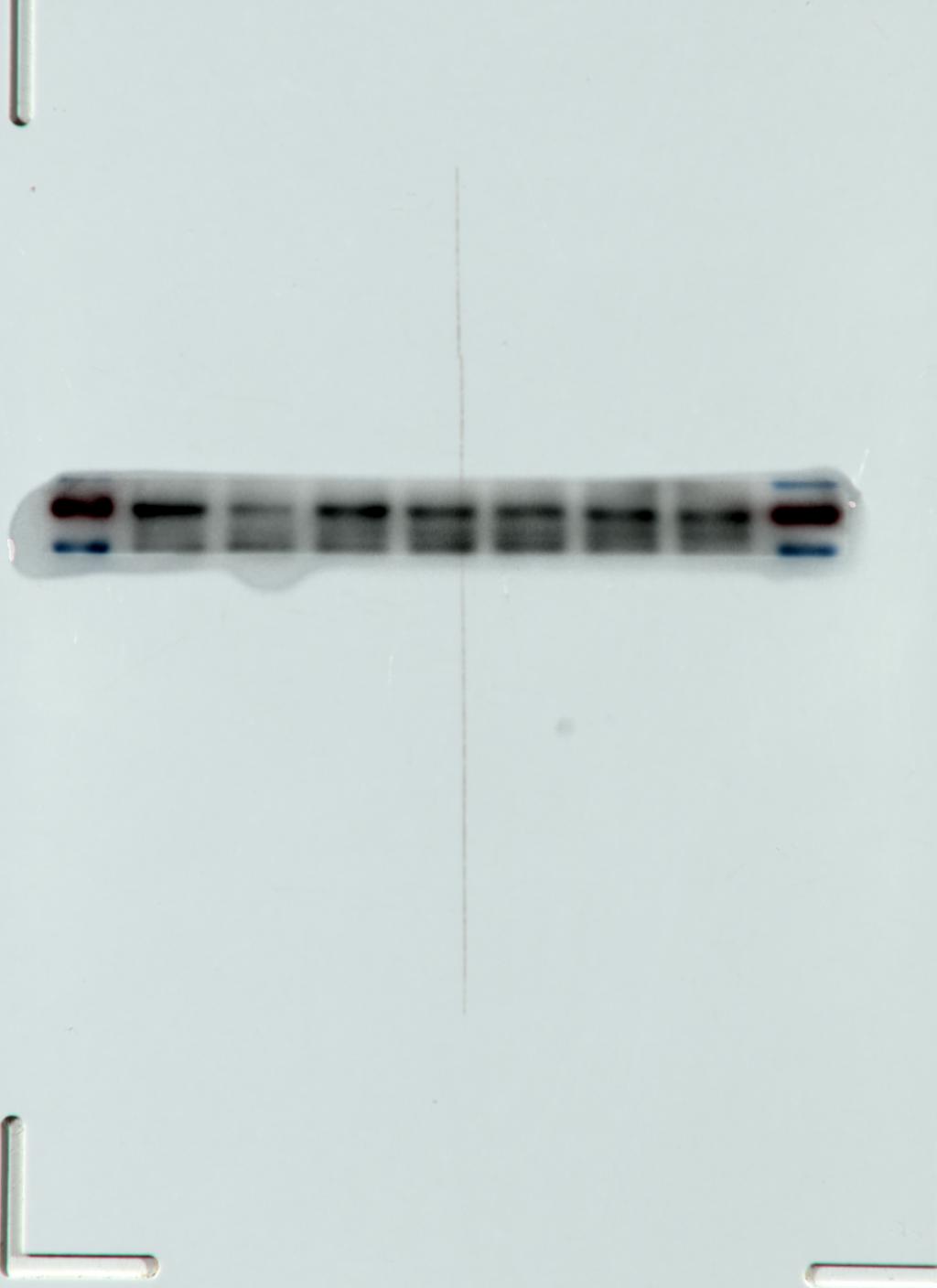


**YAP1**


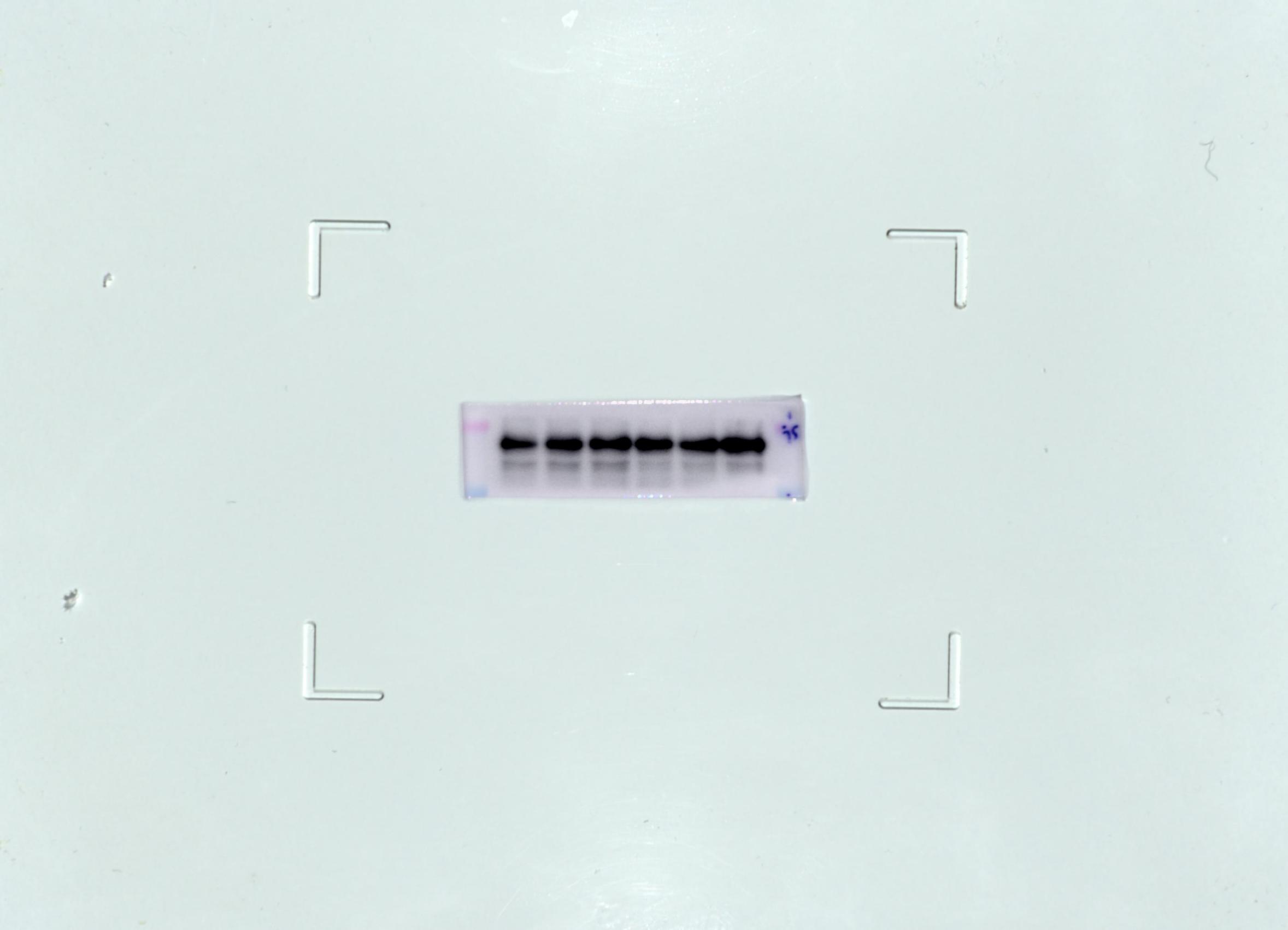


**ACTIN**

**
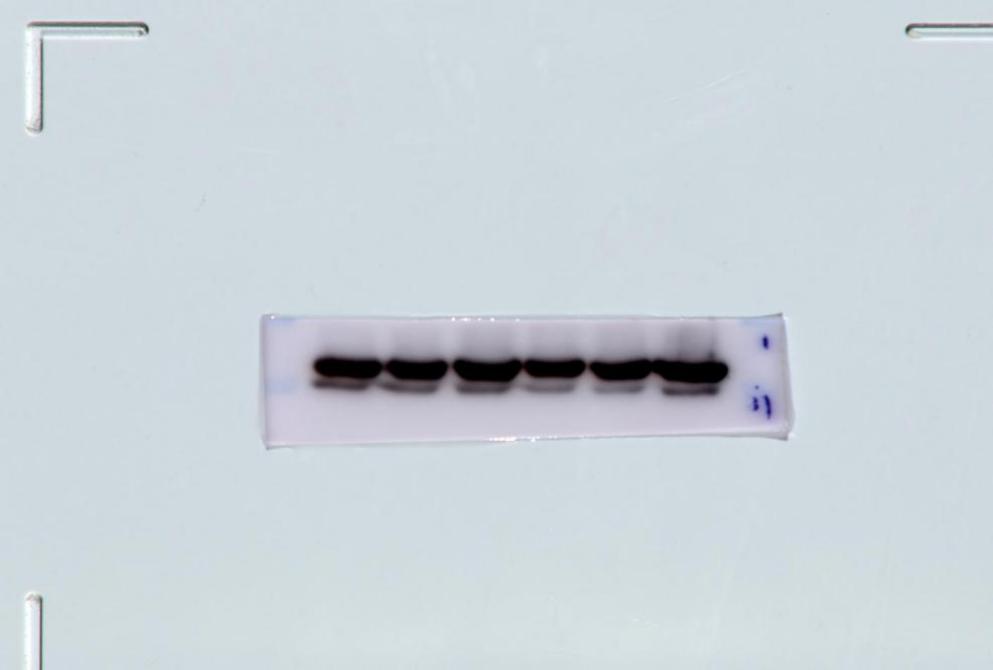
**

**Fig2F**

**pYAP1 (S127)**


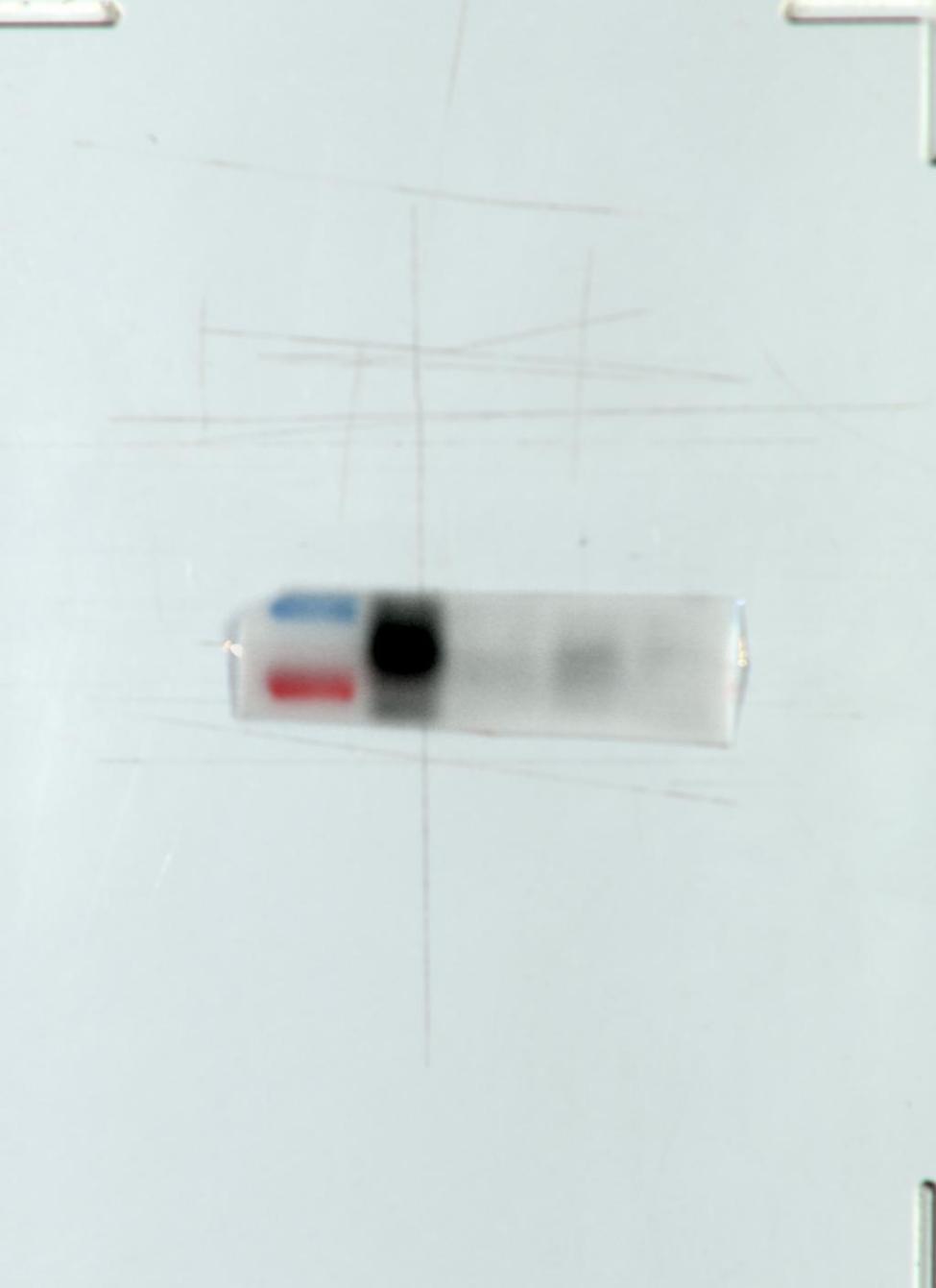


**YAP1**


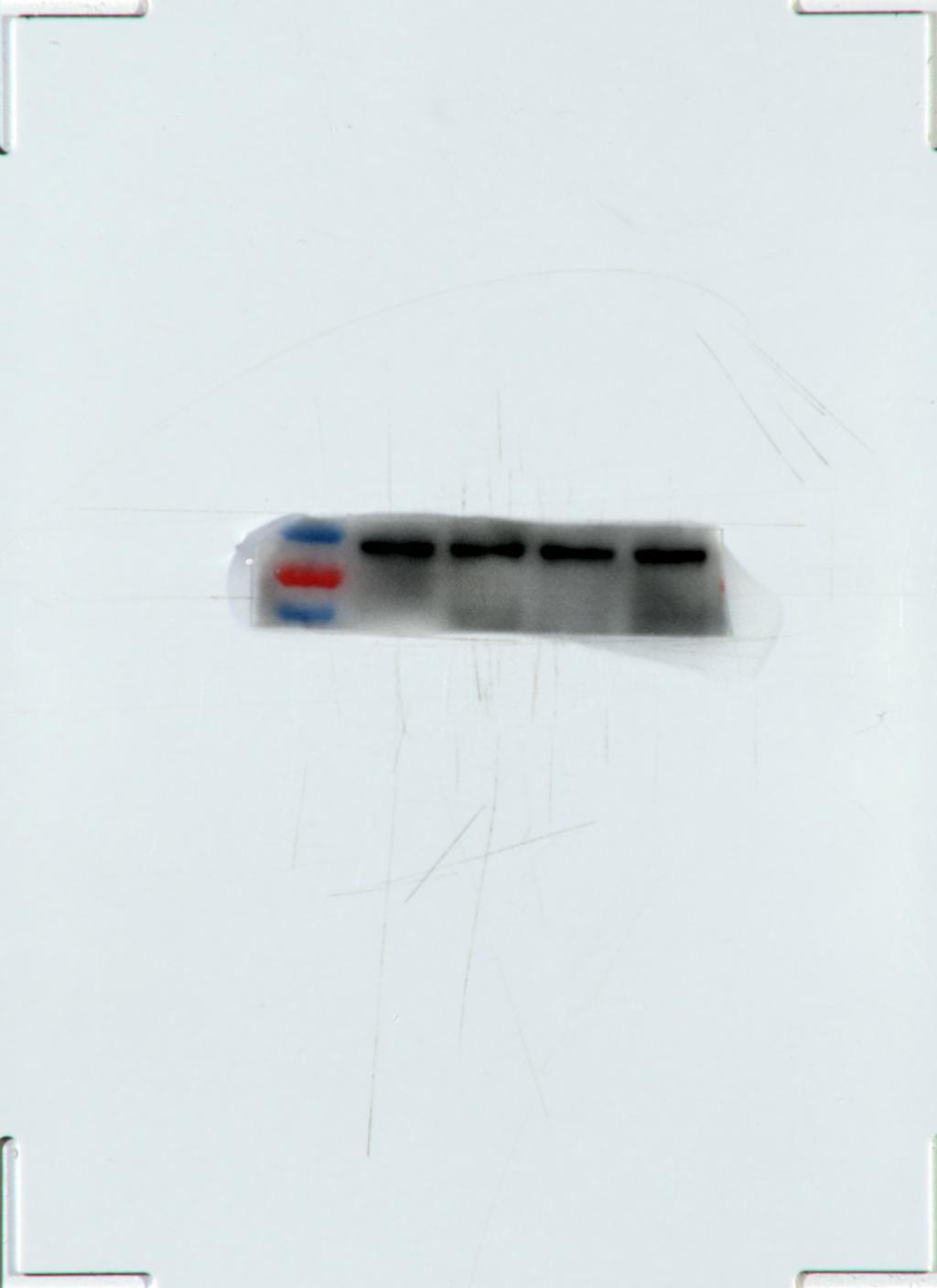


**ACTIN**


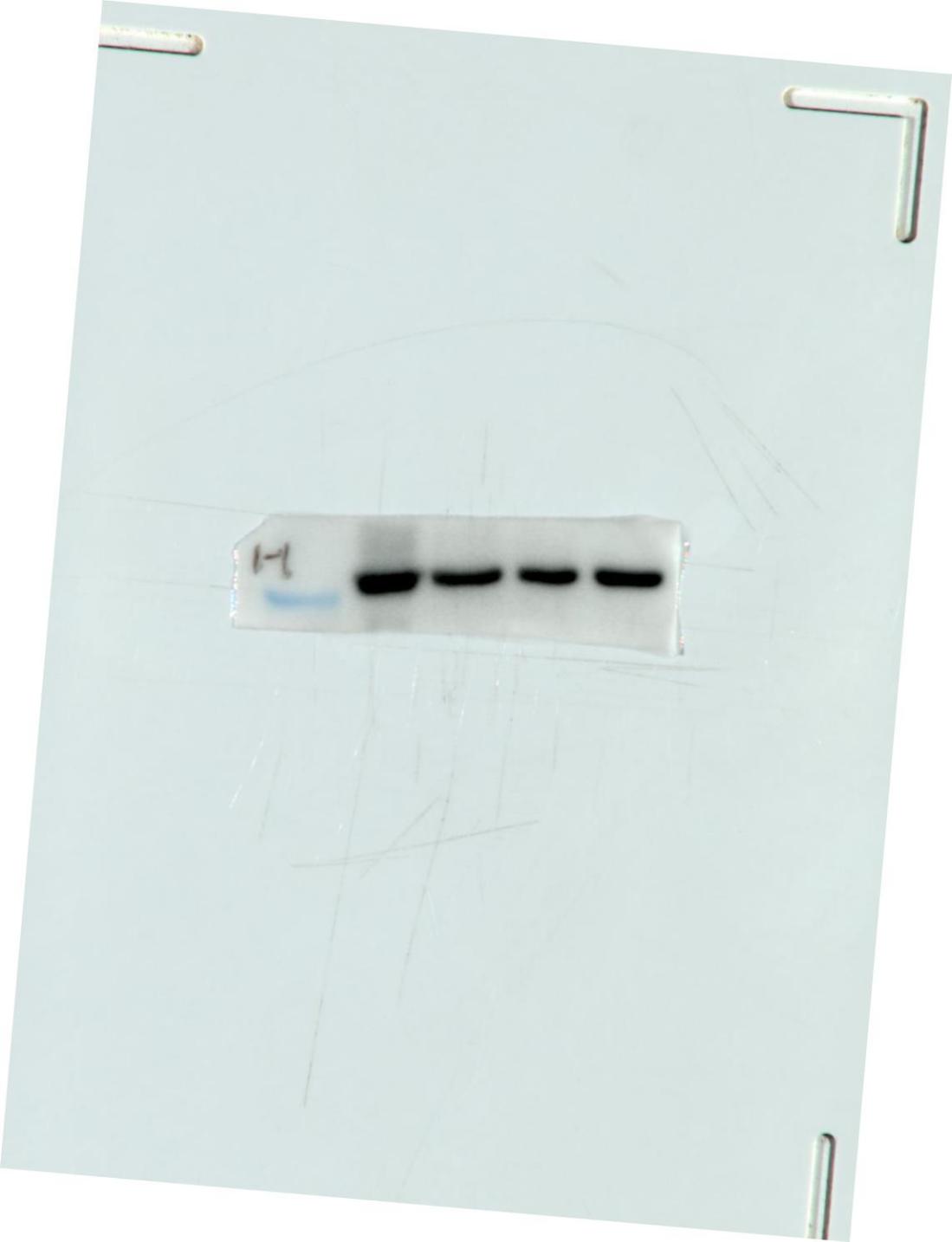


**Fig4A**

**pMST1/2**

**
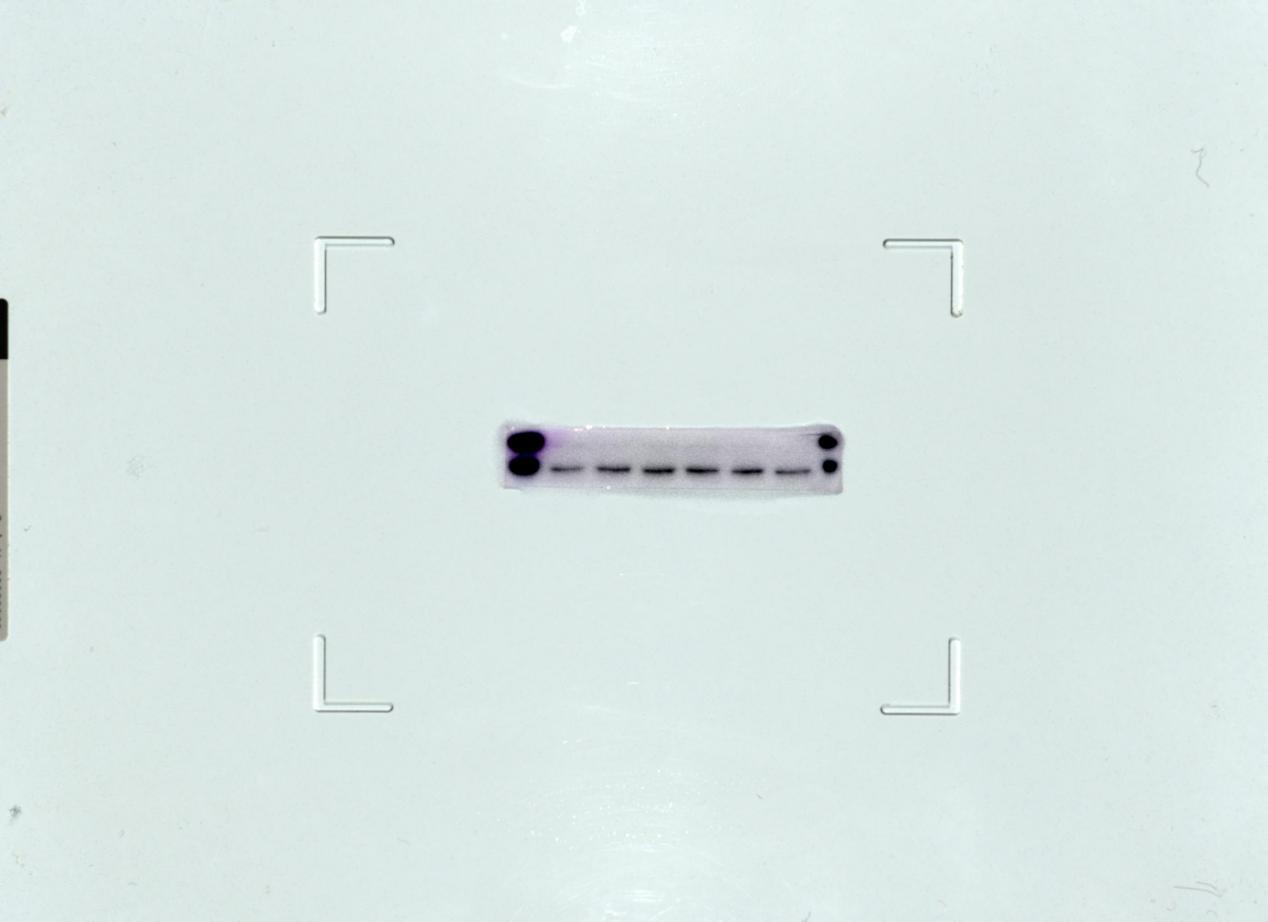
**

**MST1**

**
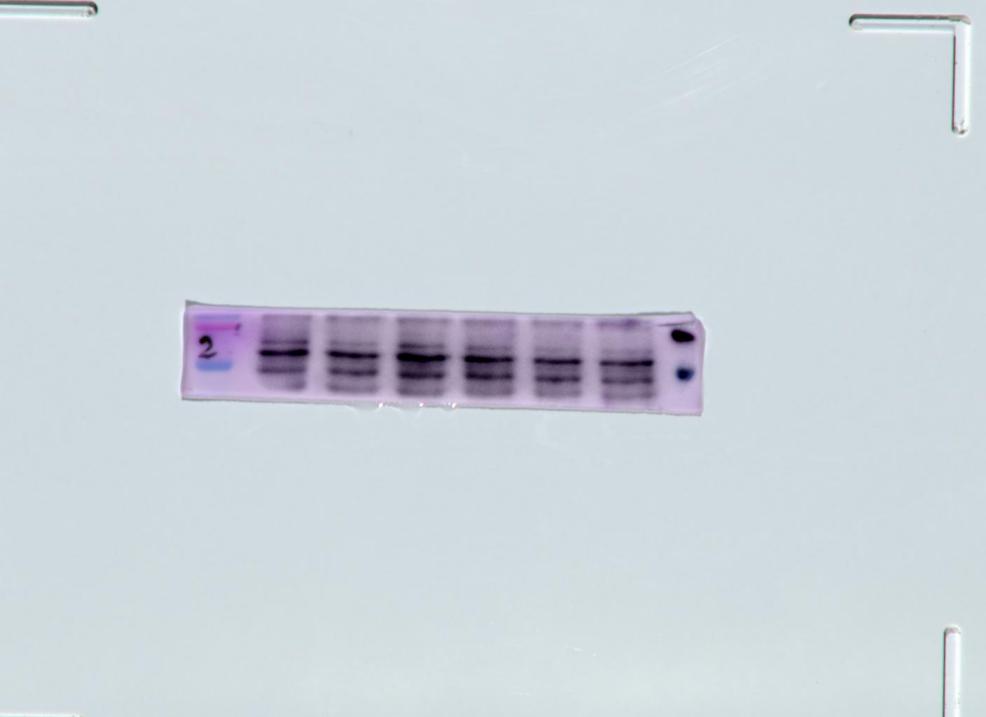
**

**pLATS1**

**
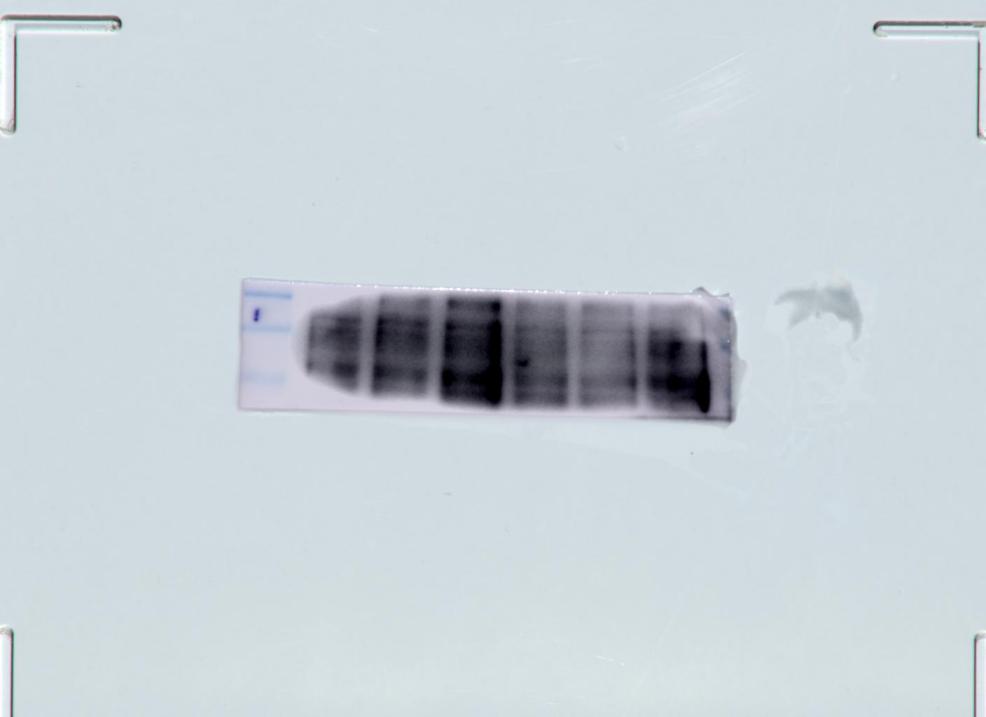
**

**LATS1**

**
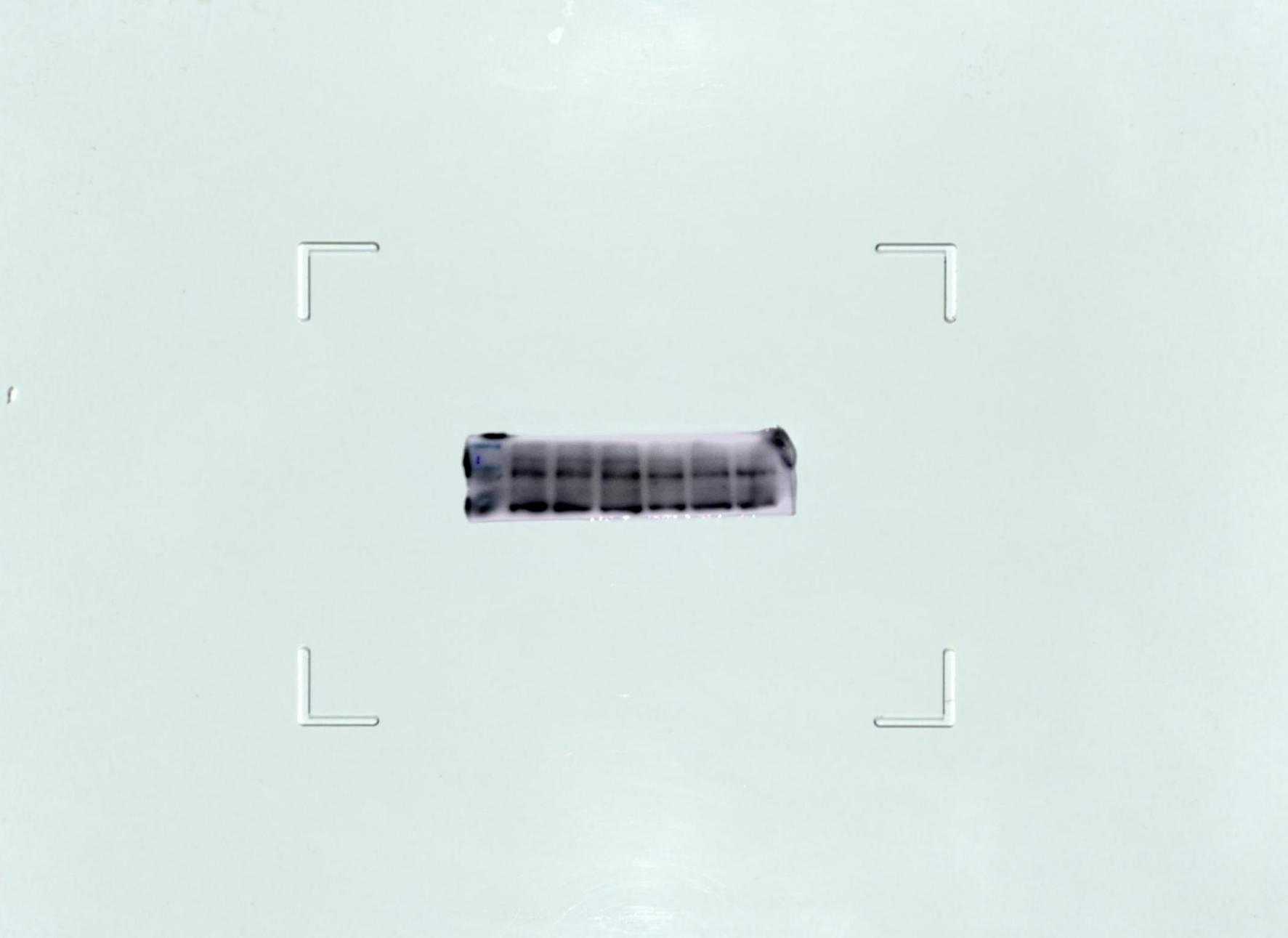
**

**LATS2**

**
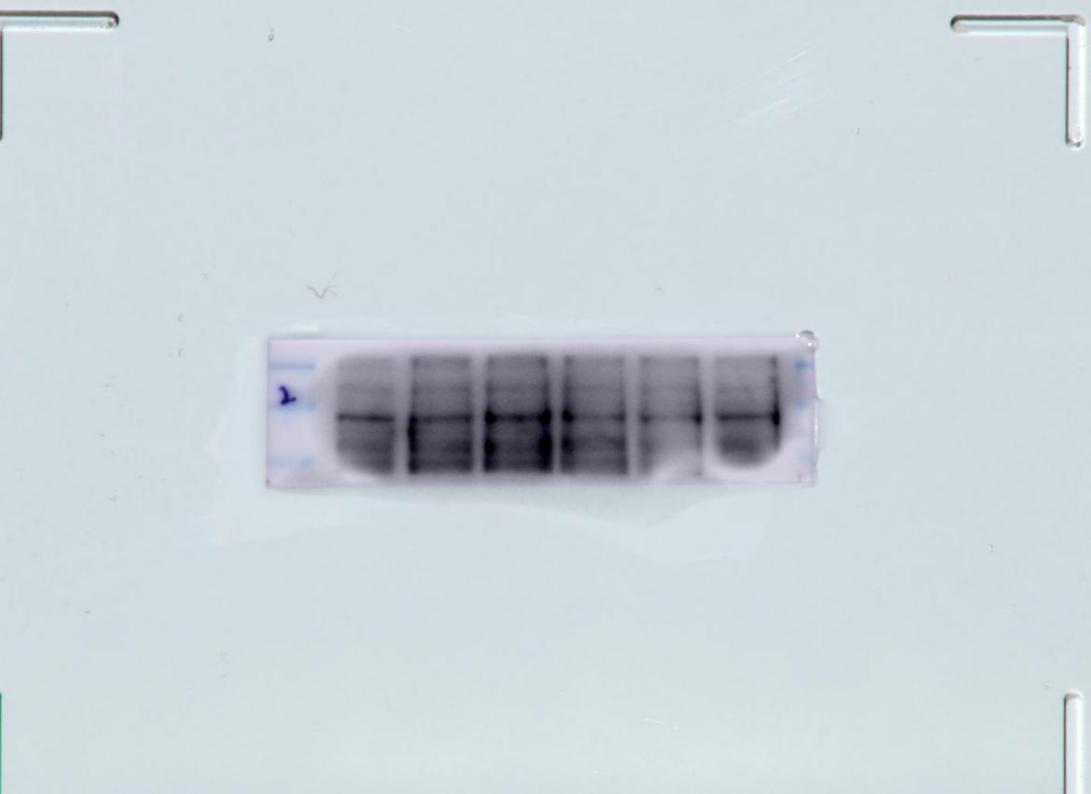
**

**Actin**

**
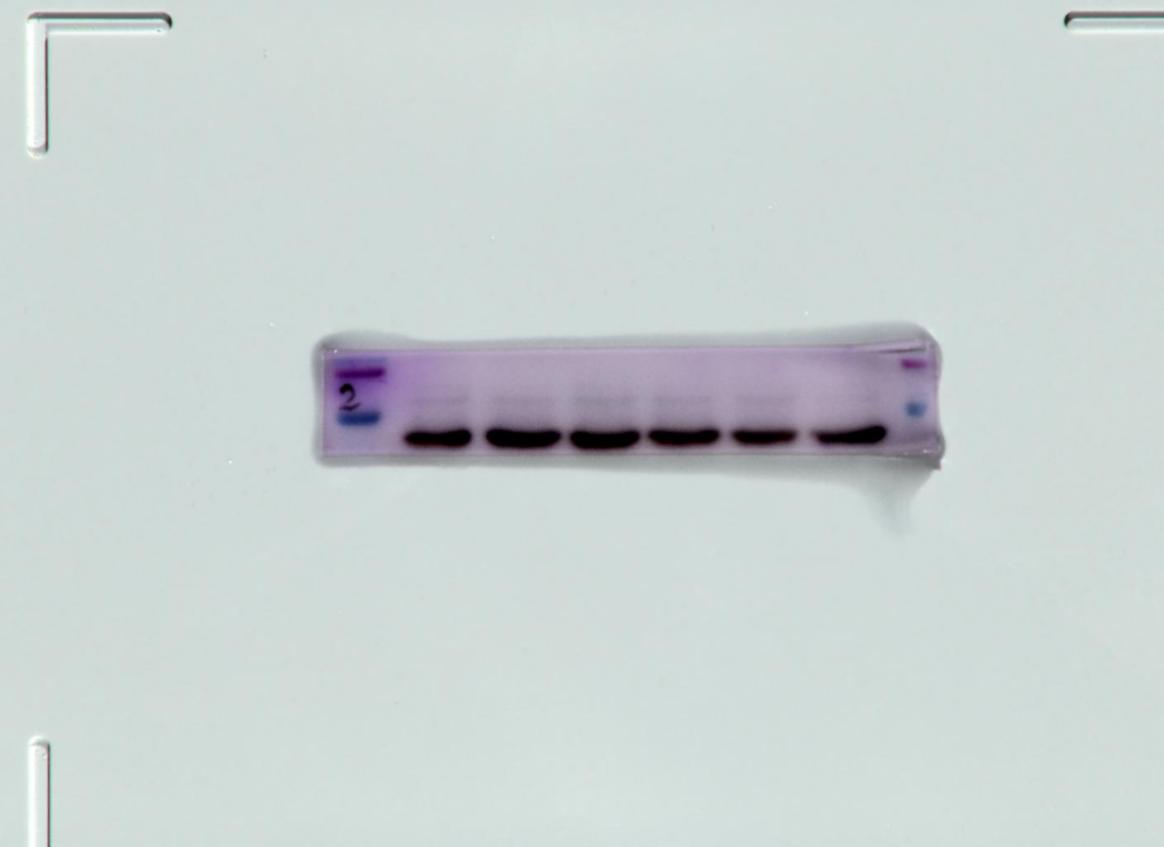
**

**Fig4E**

**pMST1/2**

**
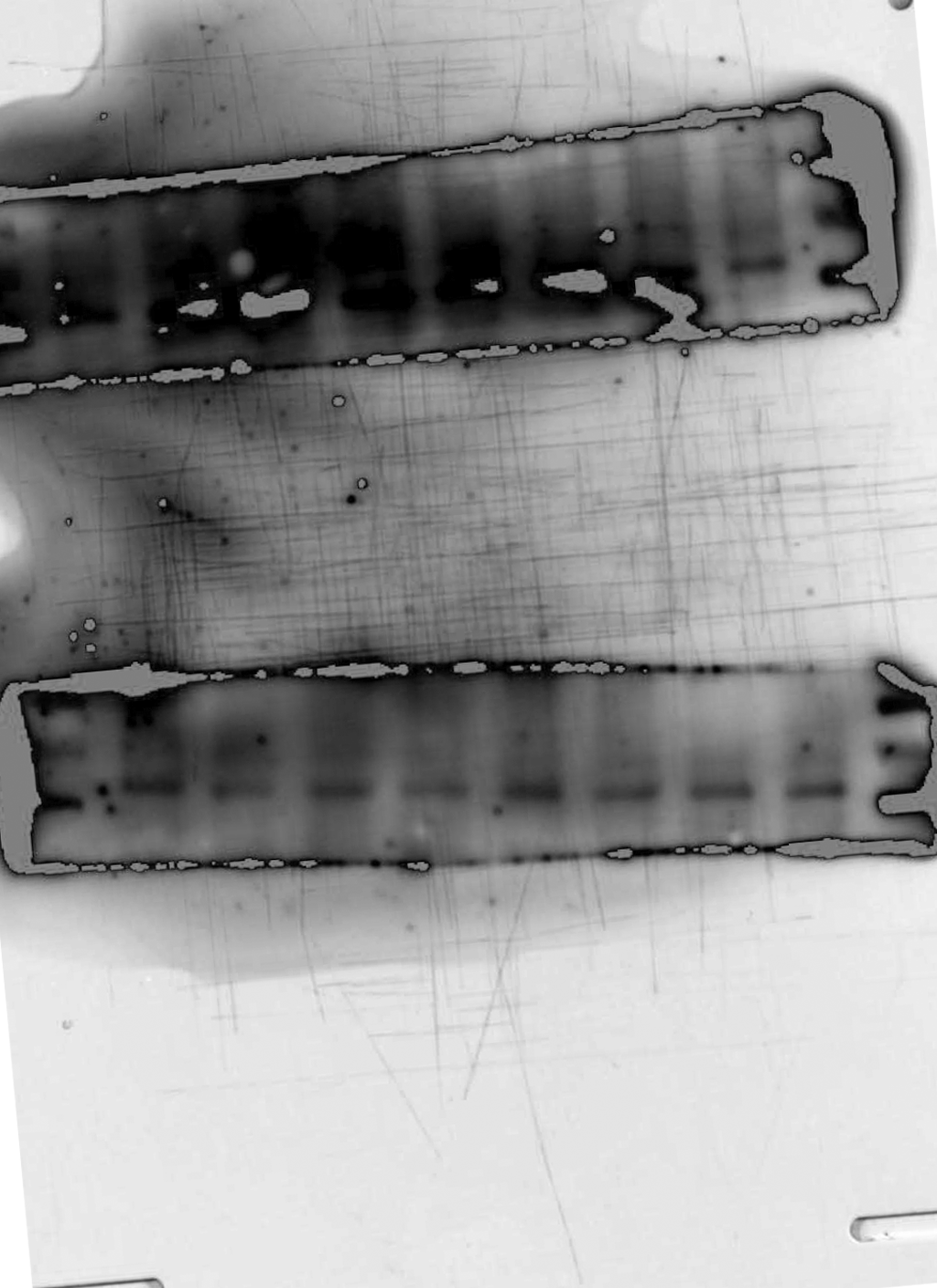
**

**MST1**

**
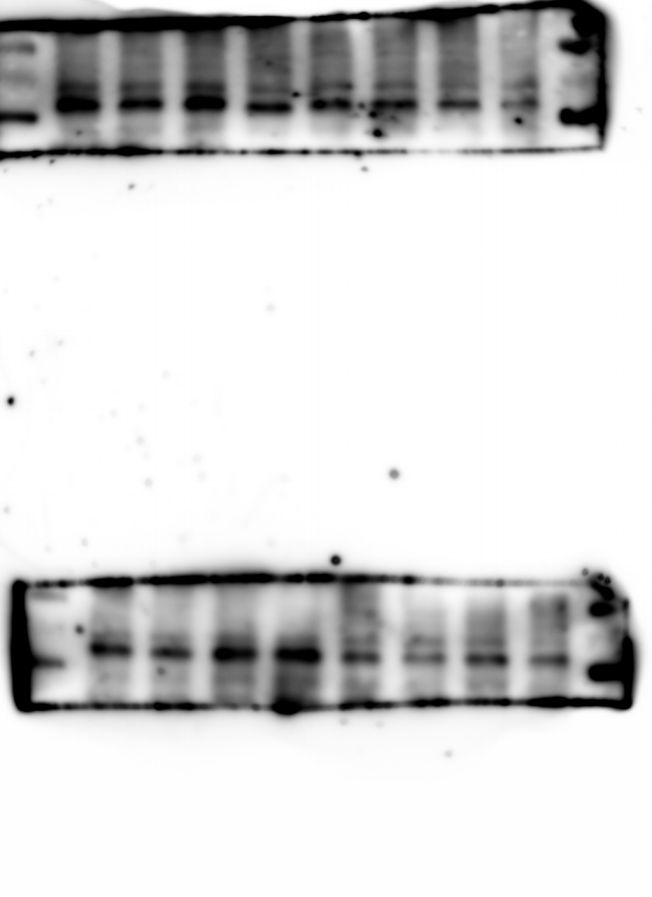
**

**GAPDH**


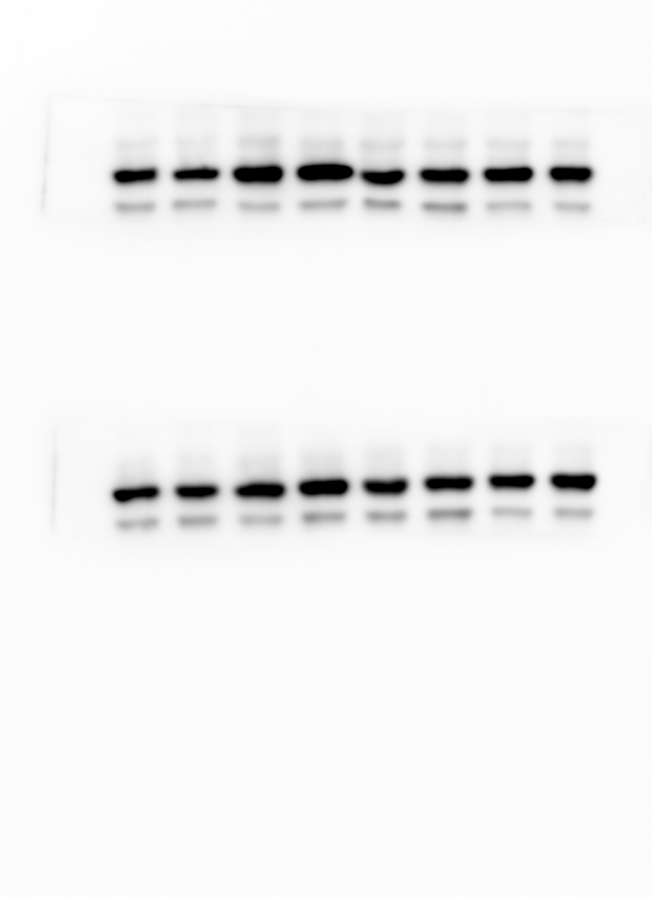


**Fig4F**

**MST1**


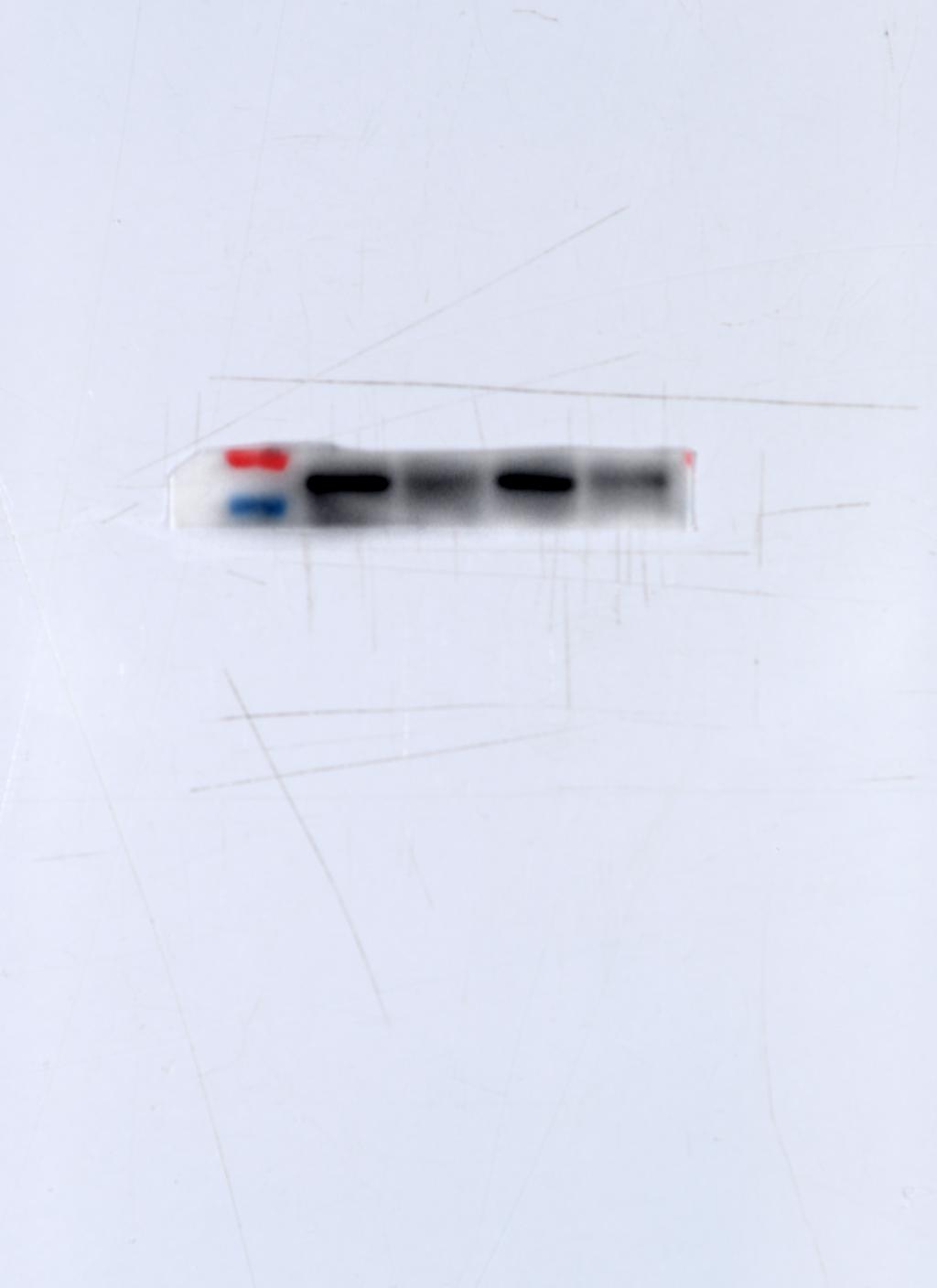


**MST2**

**
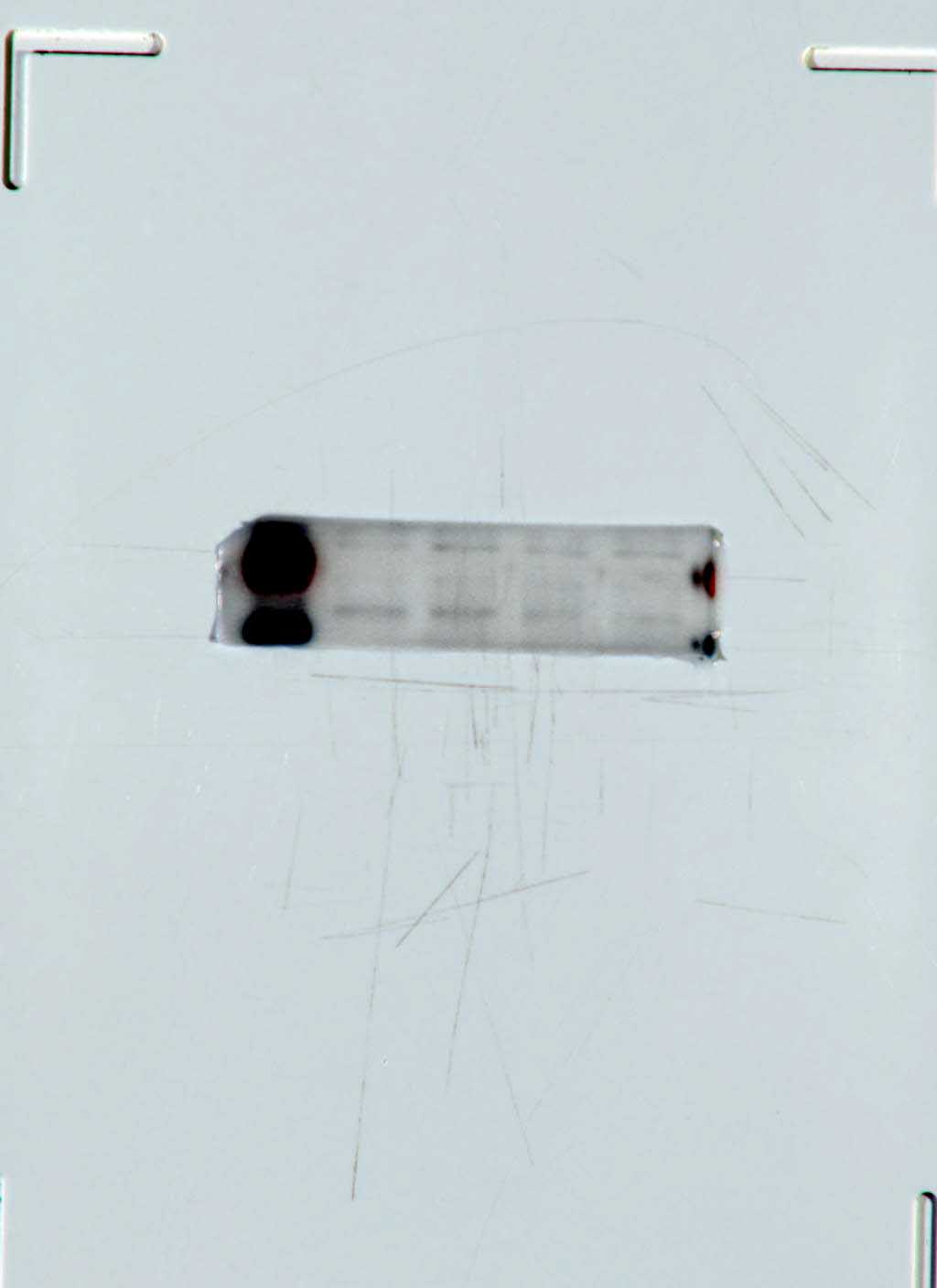
**

**pYAP1(S127)**


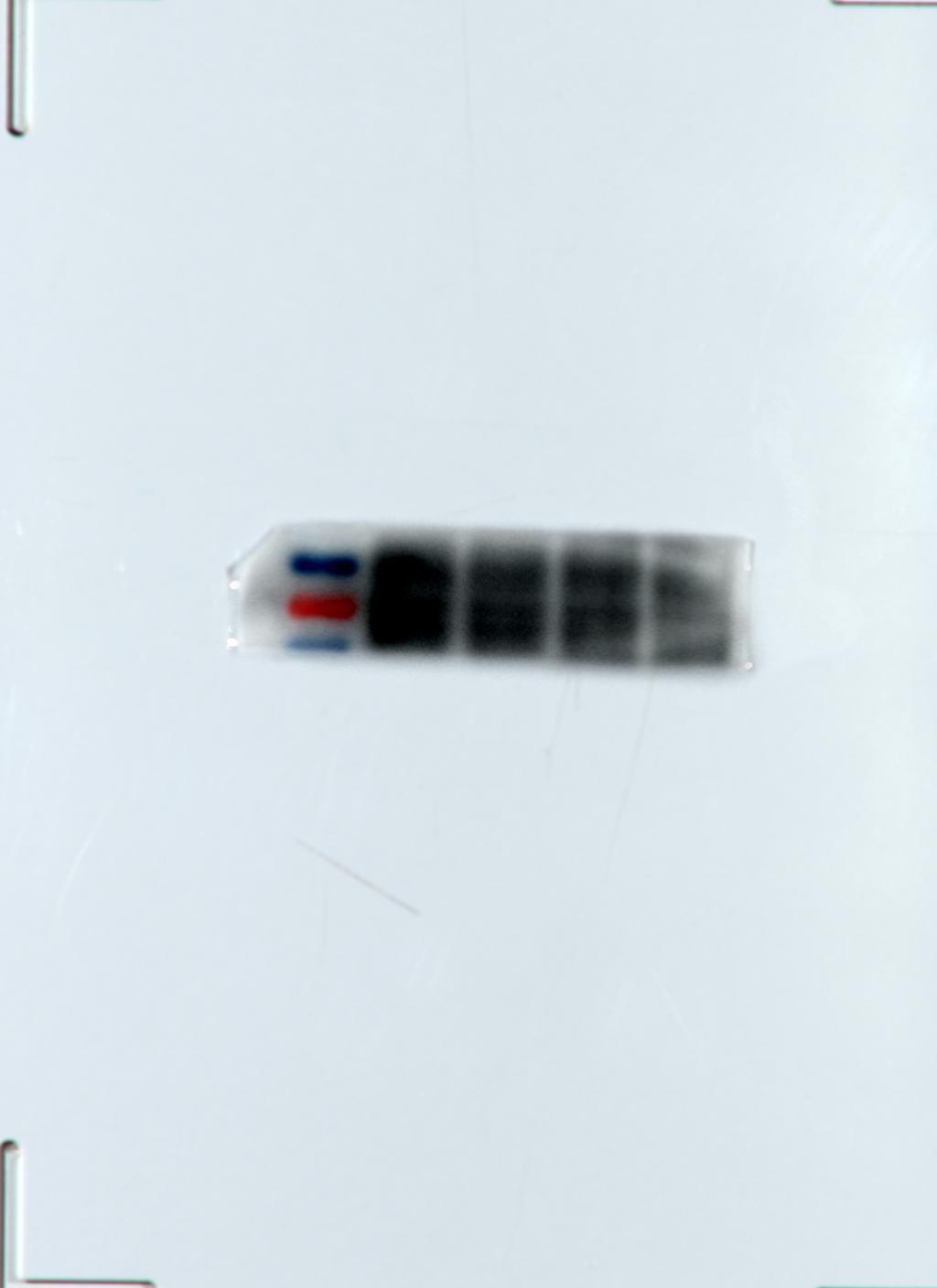


**YAP1**


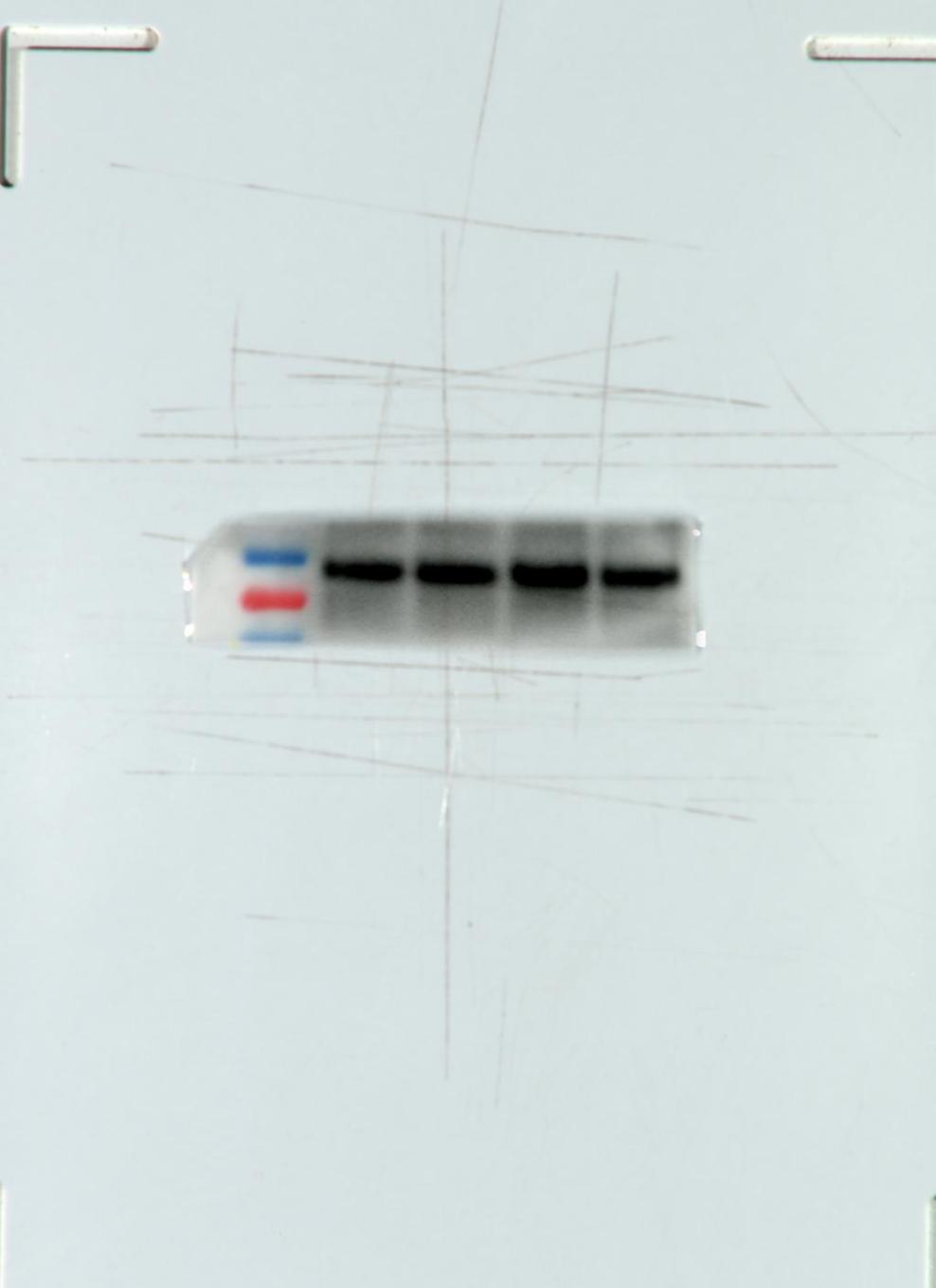


**ACTIN**


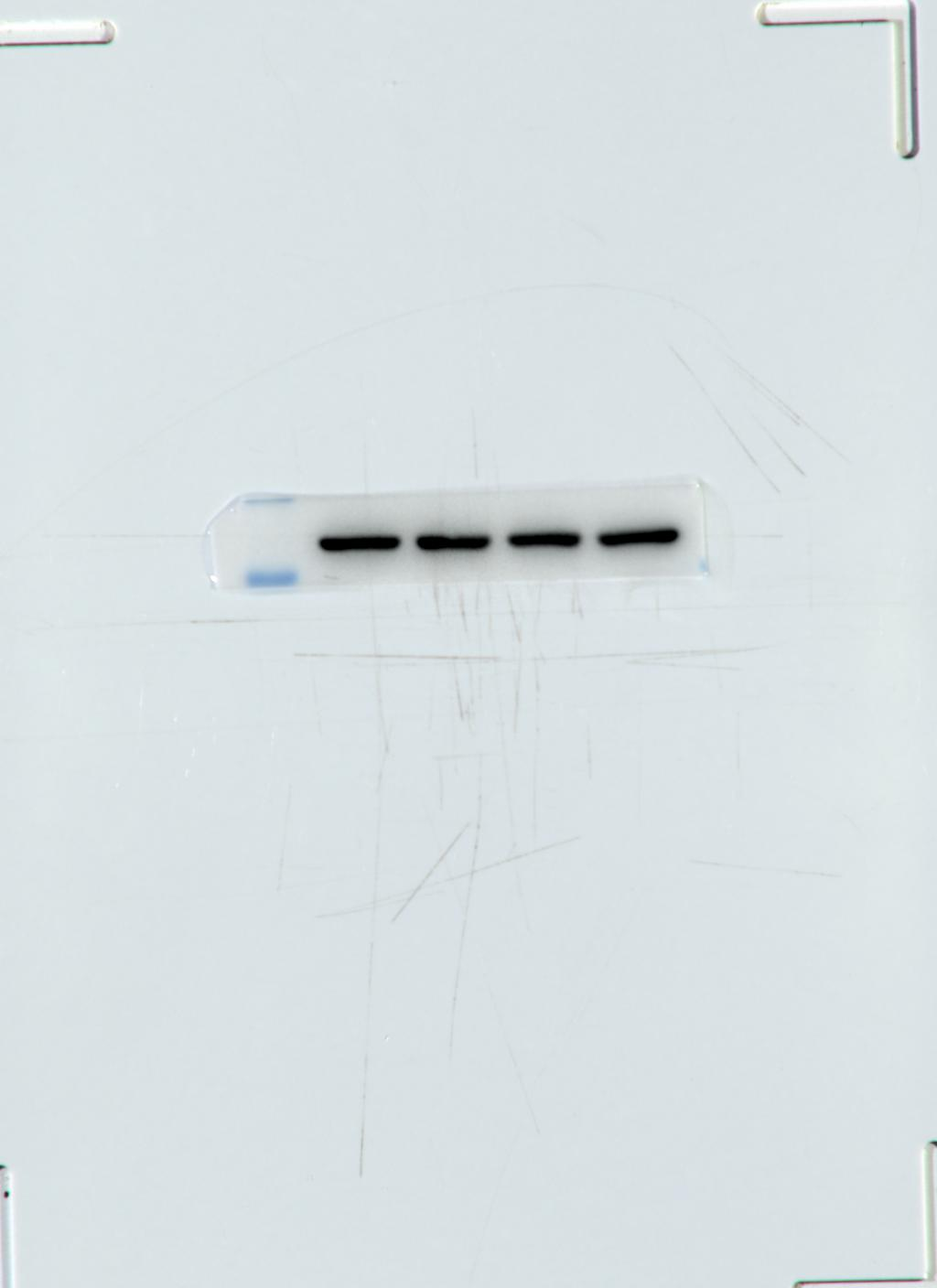


**Fig4I**

**pAKT (S473)**


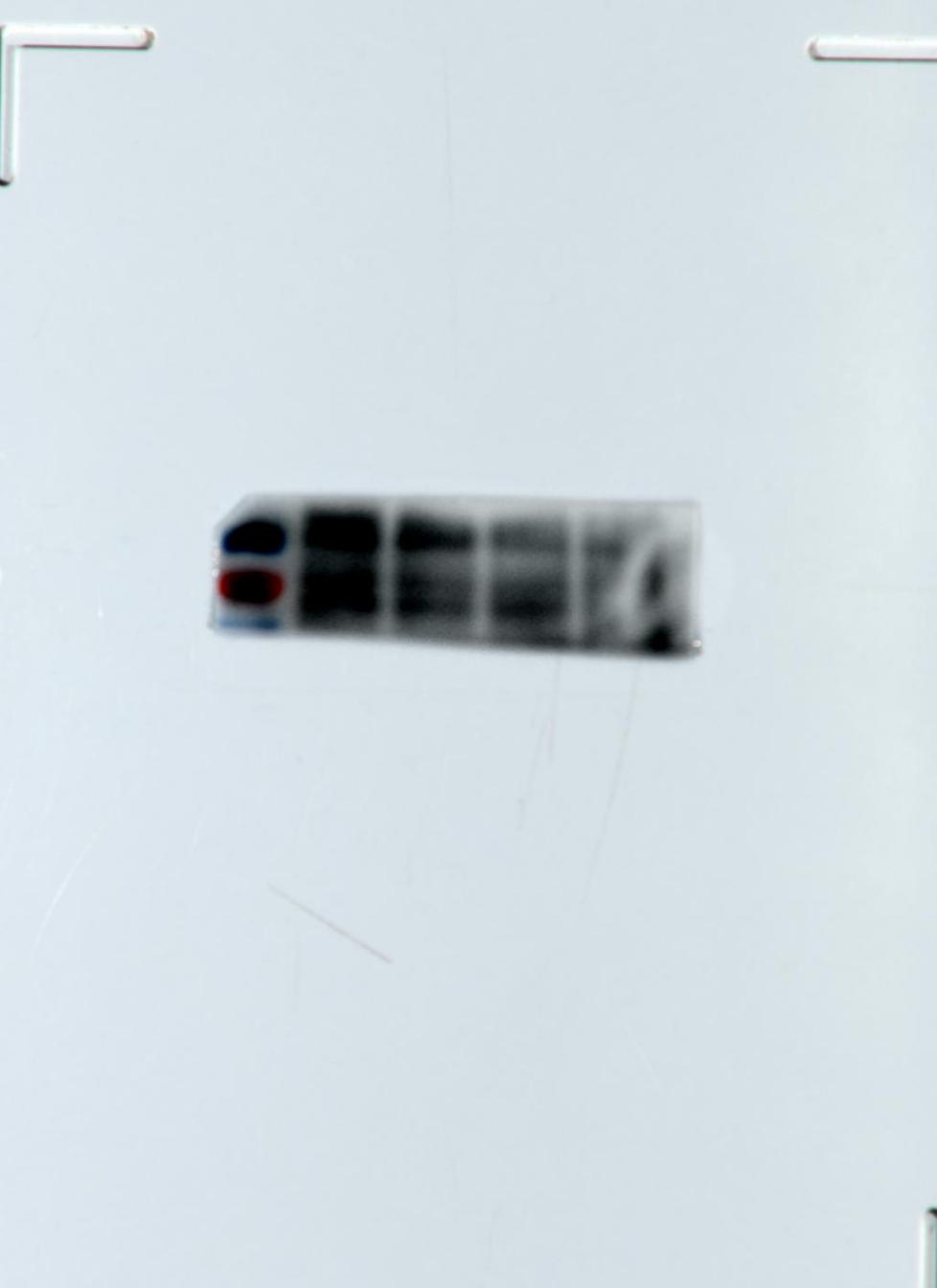


**AKT**


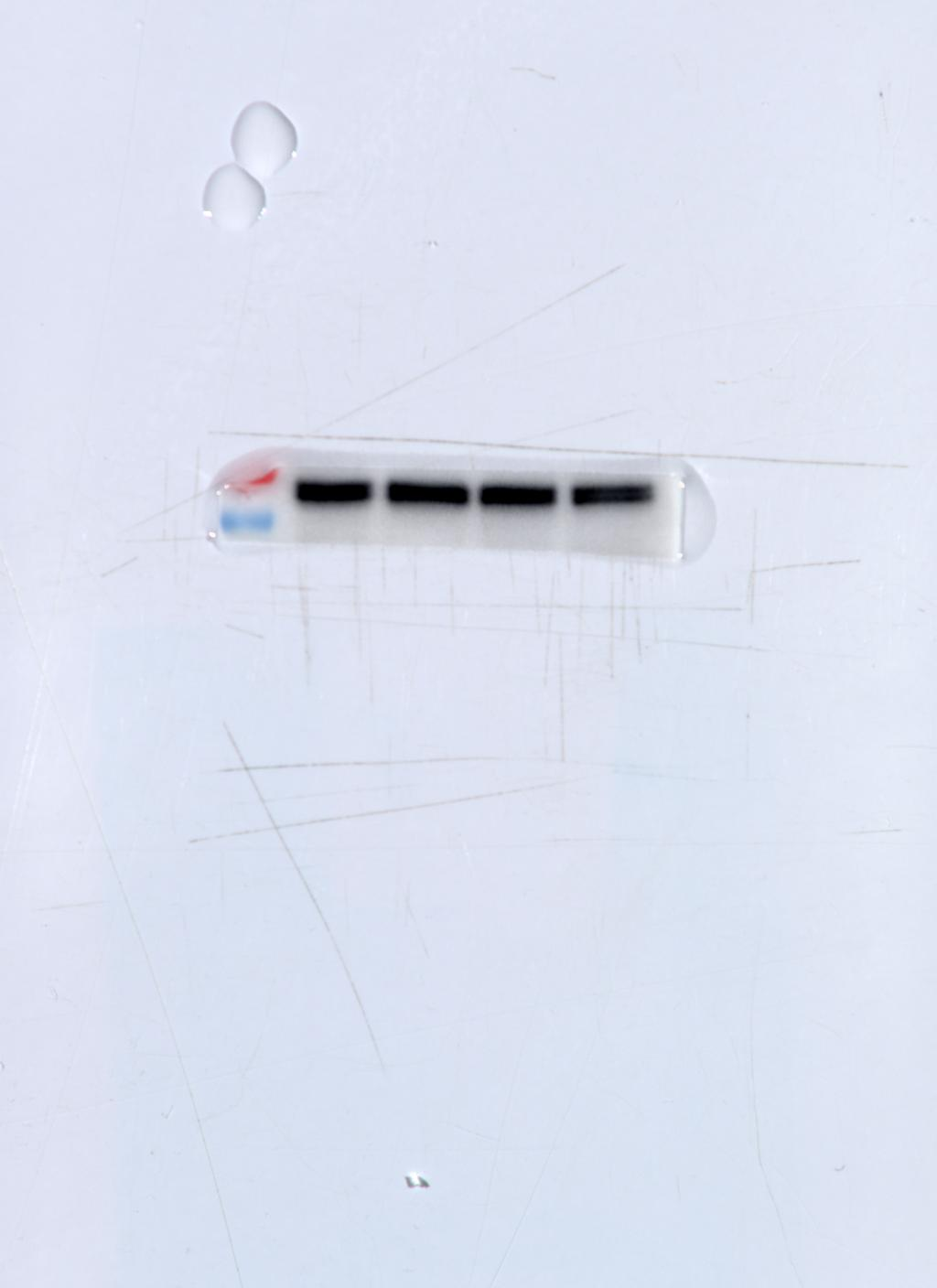


**ACTIN**

**
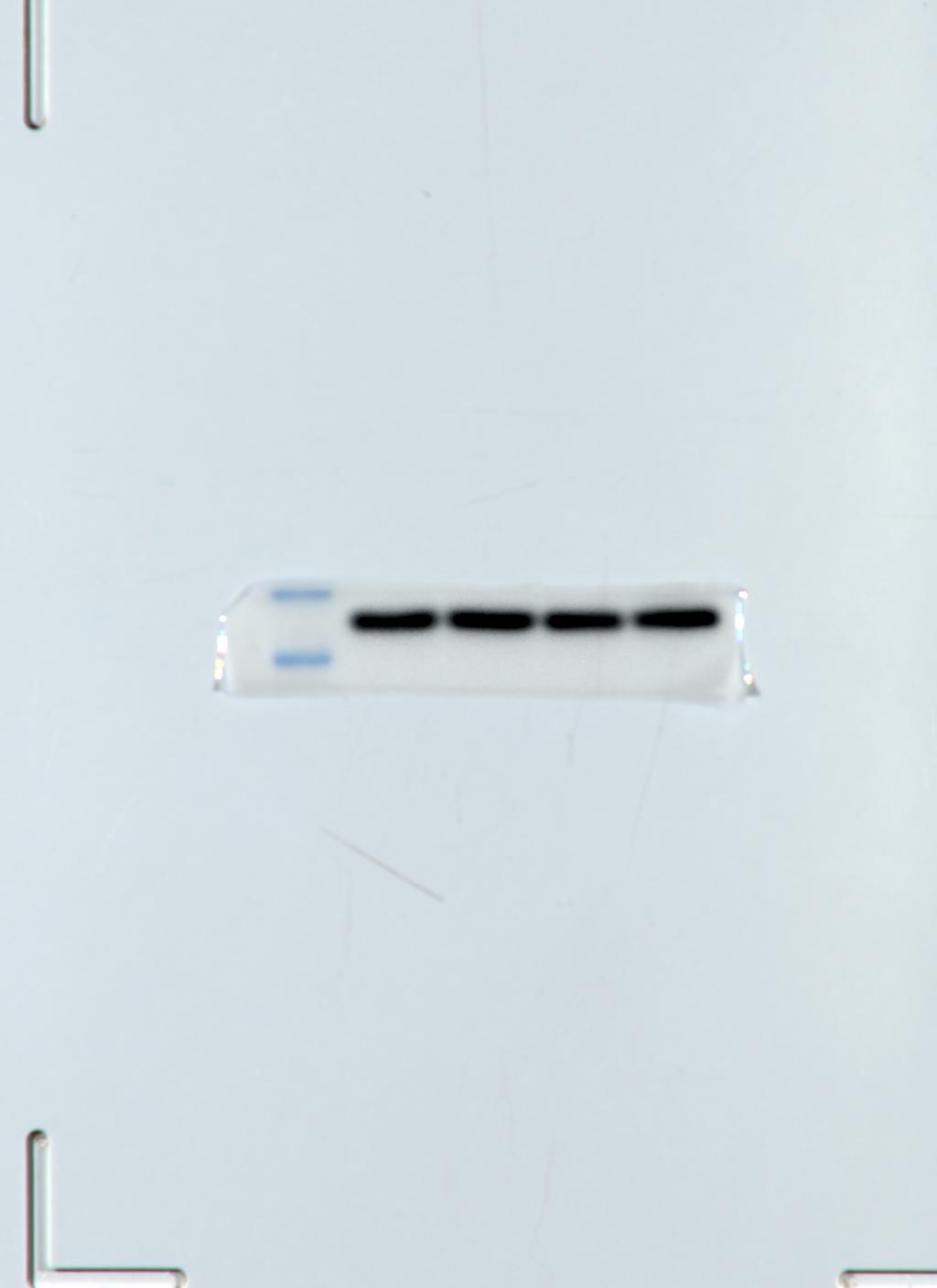
**

**Fig 5A**

**pYAP1(S127)**


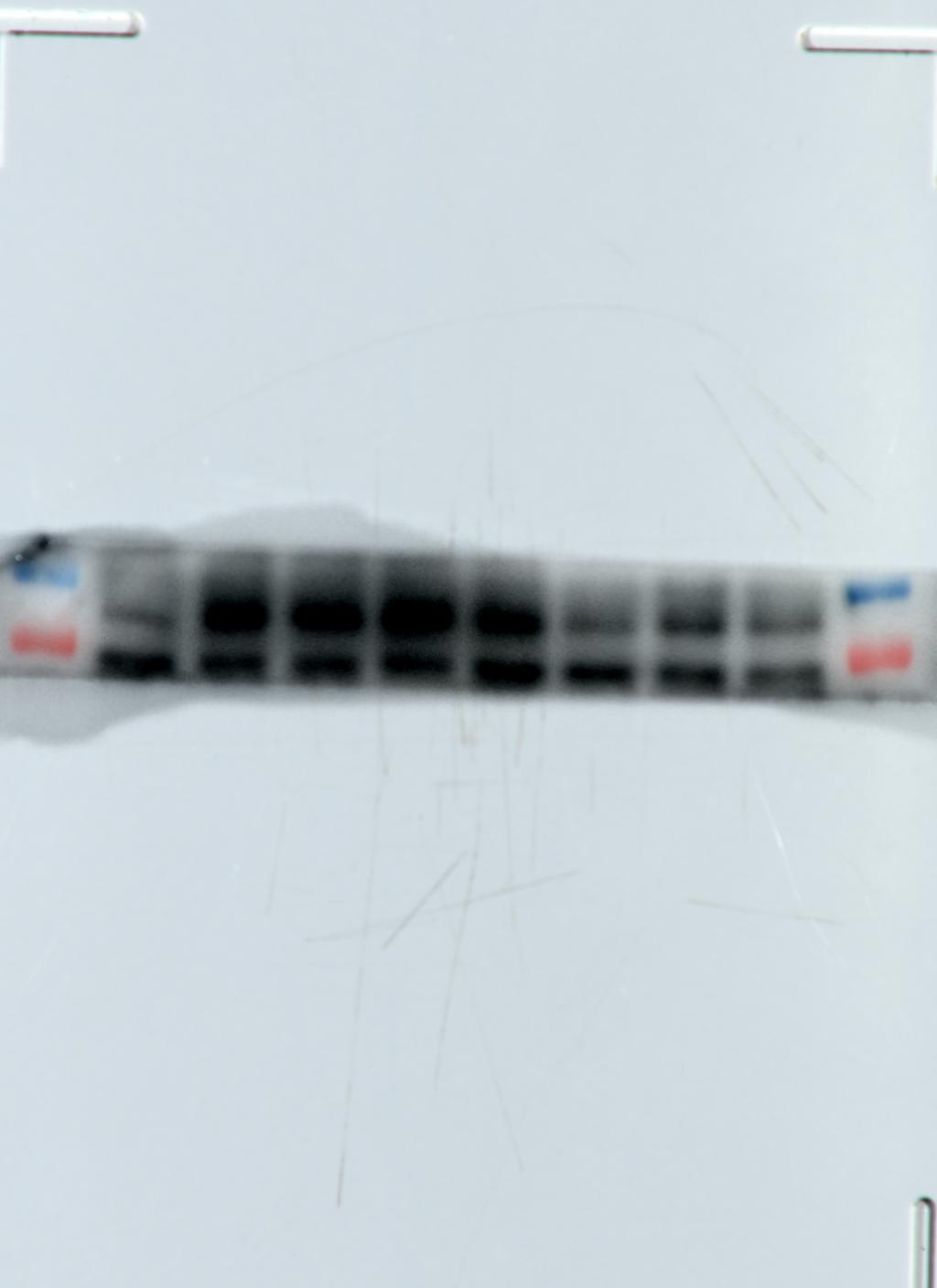


**YAP1**


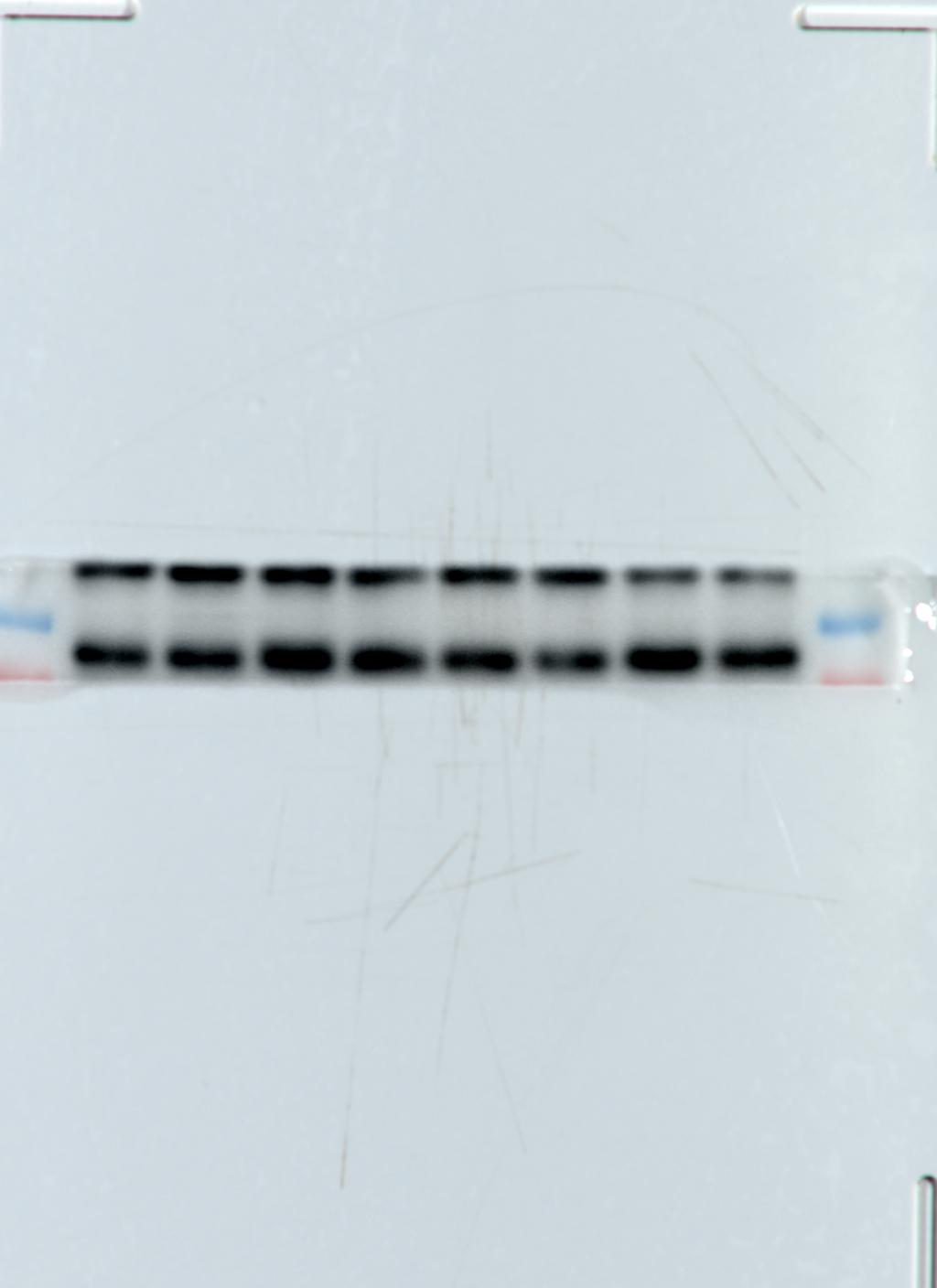


**HA**


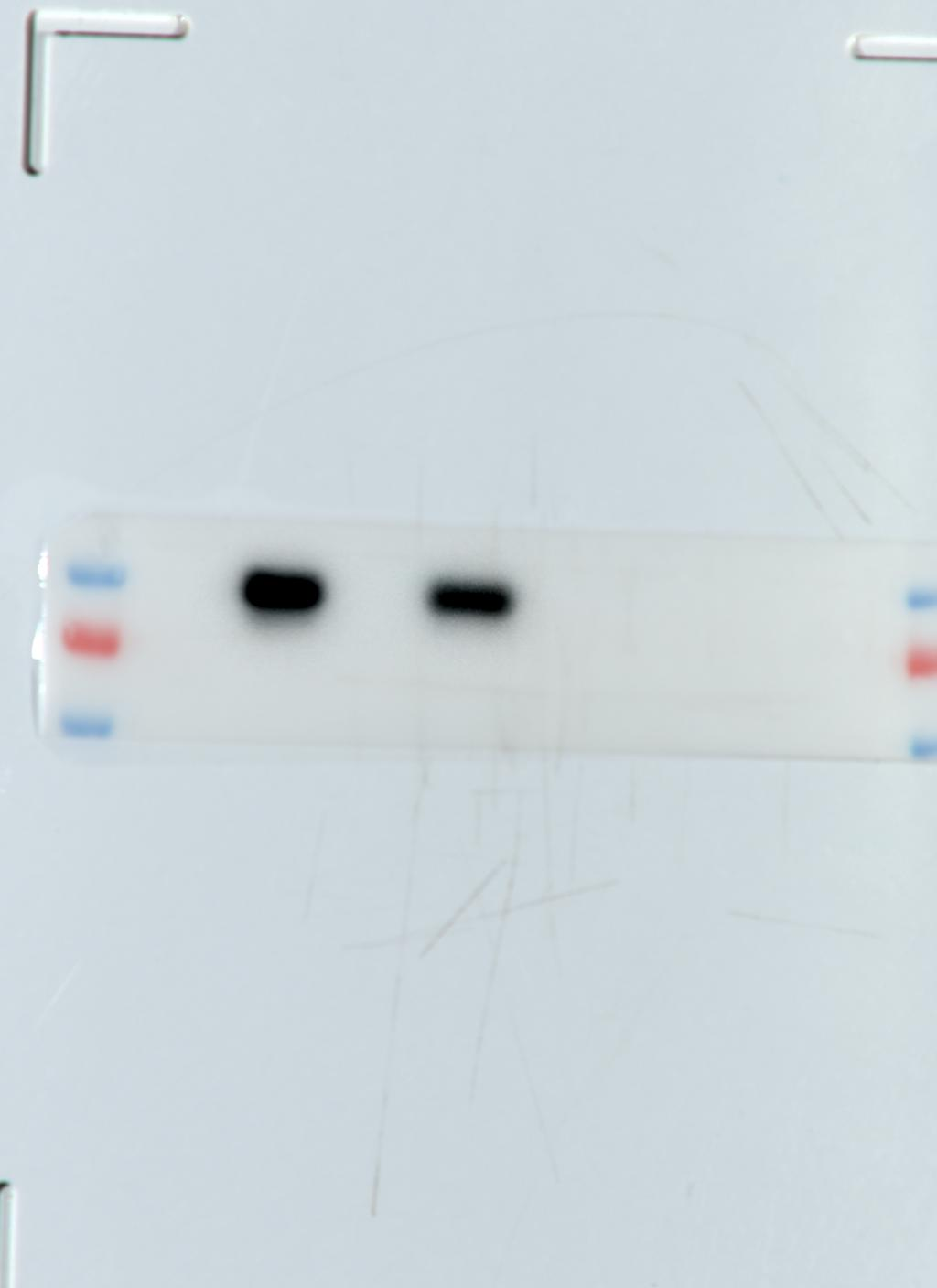


**pAKT**

**
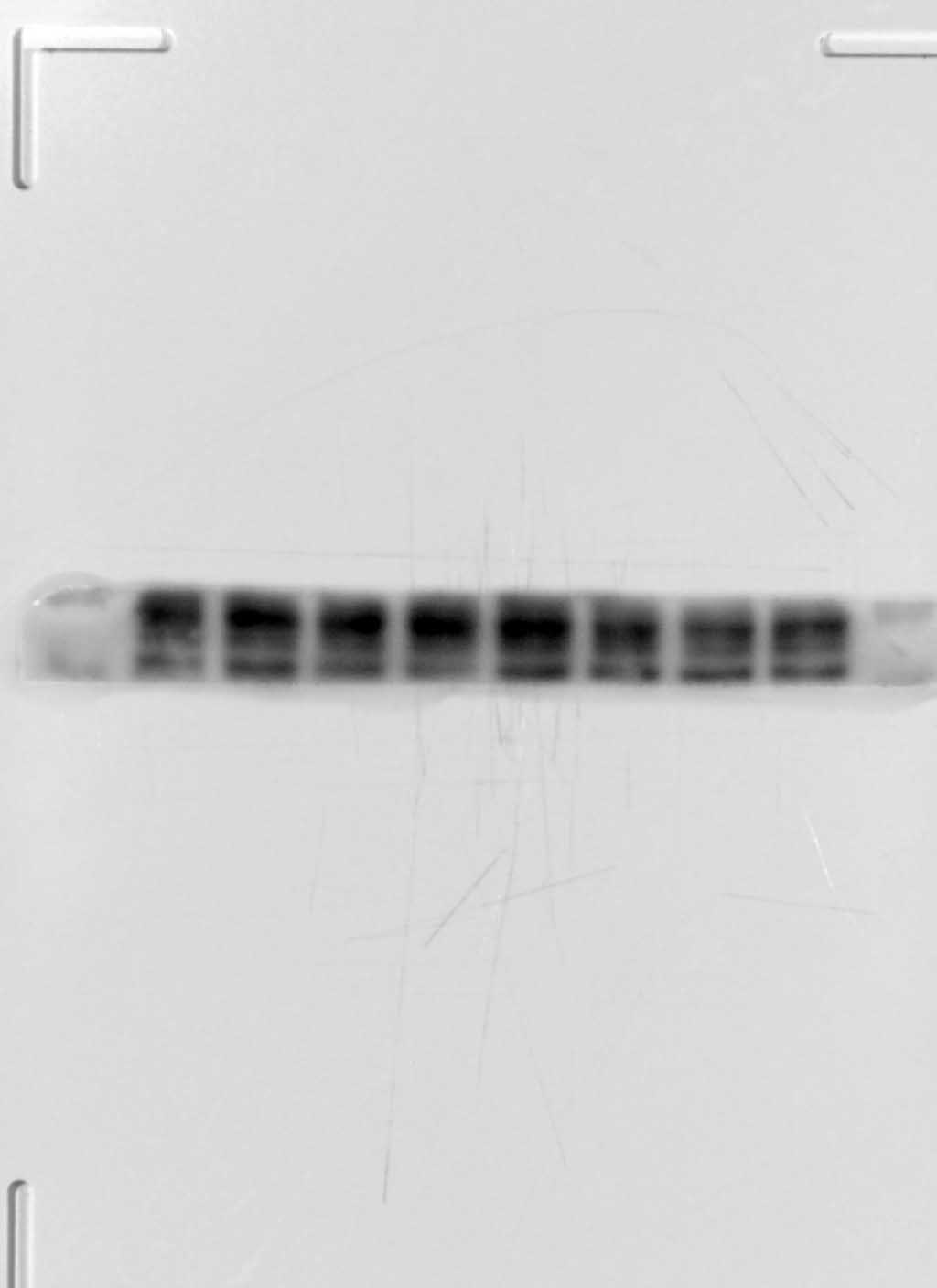
**

**AKT**


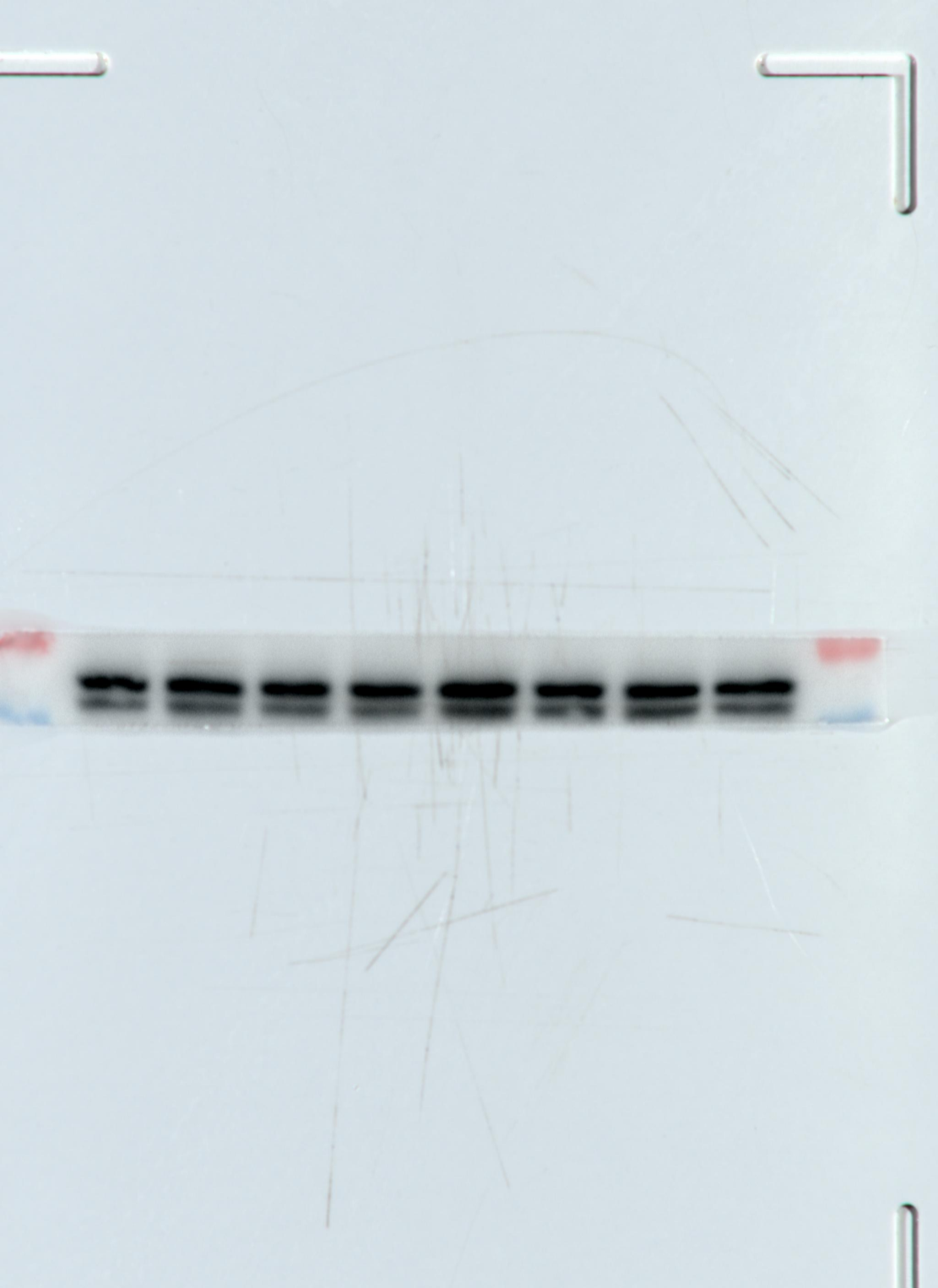


**β-ACTIN**


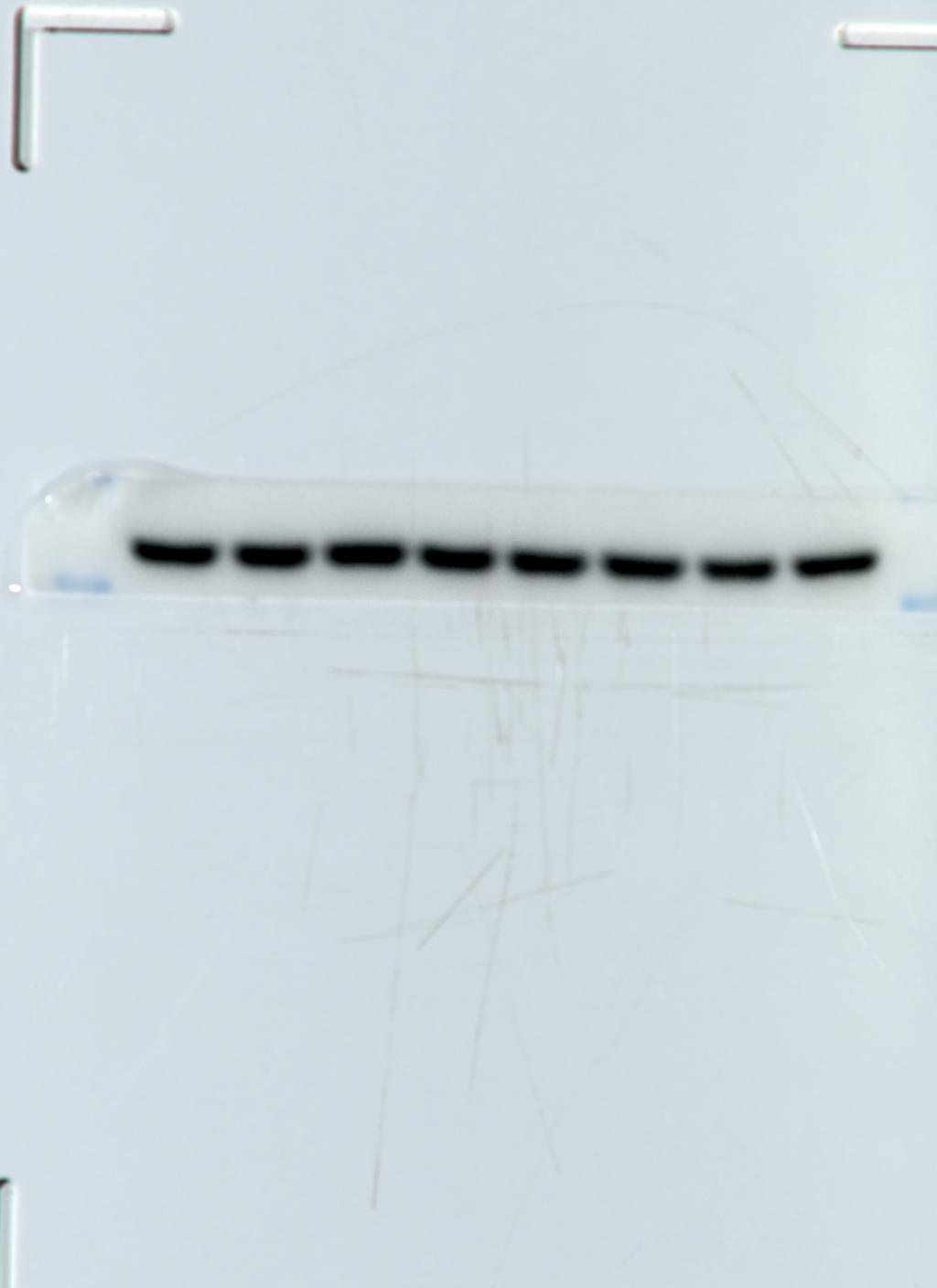


**Fig 5B**

**pYAP1**


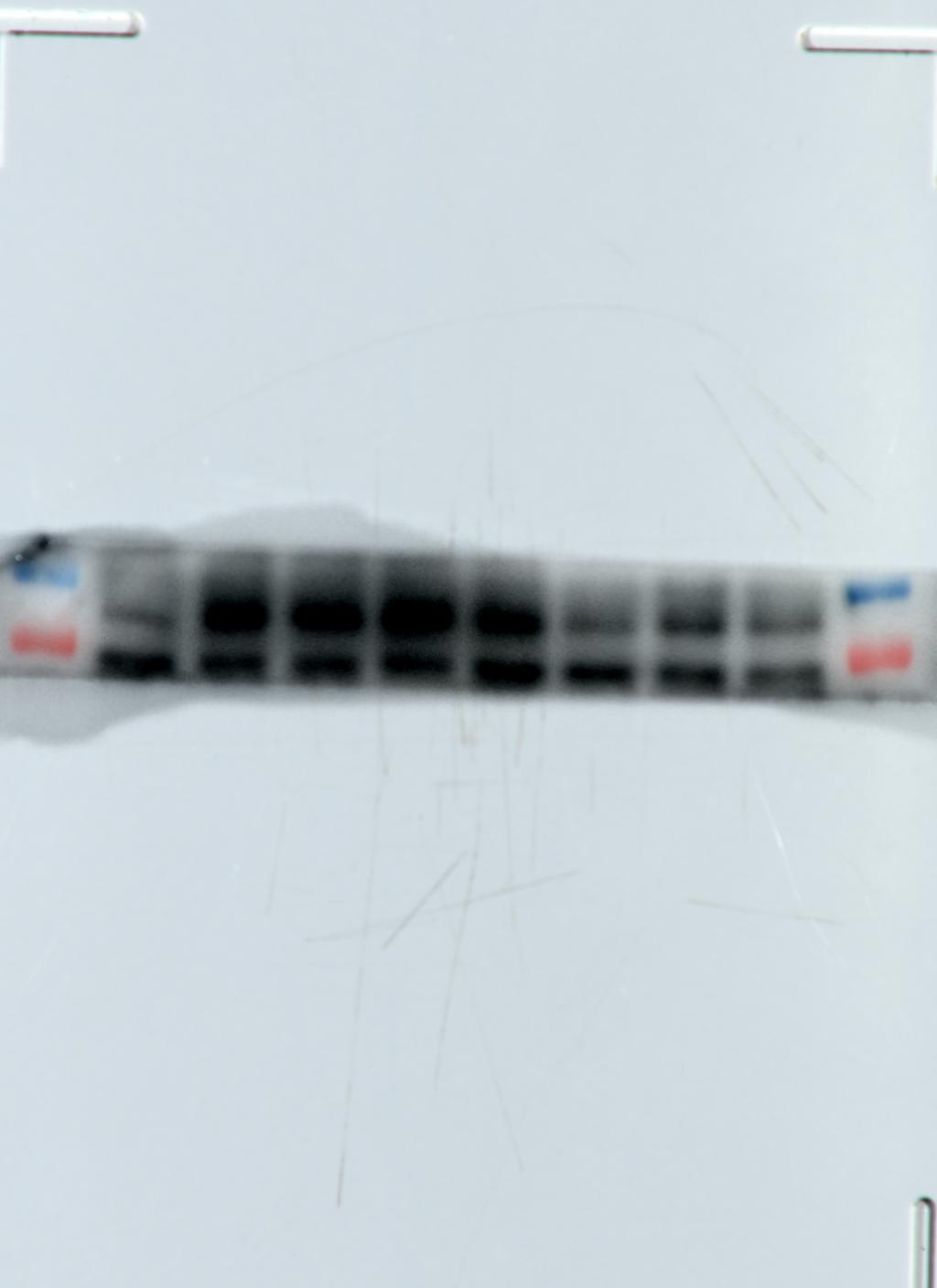


**YAP1**


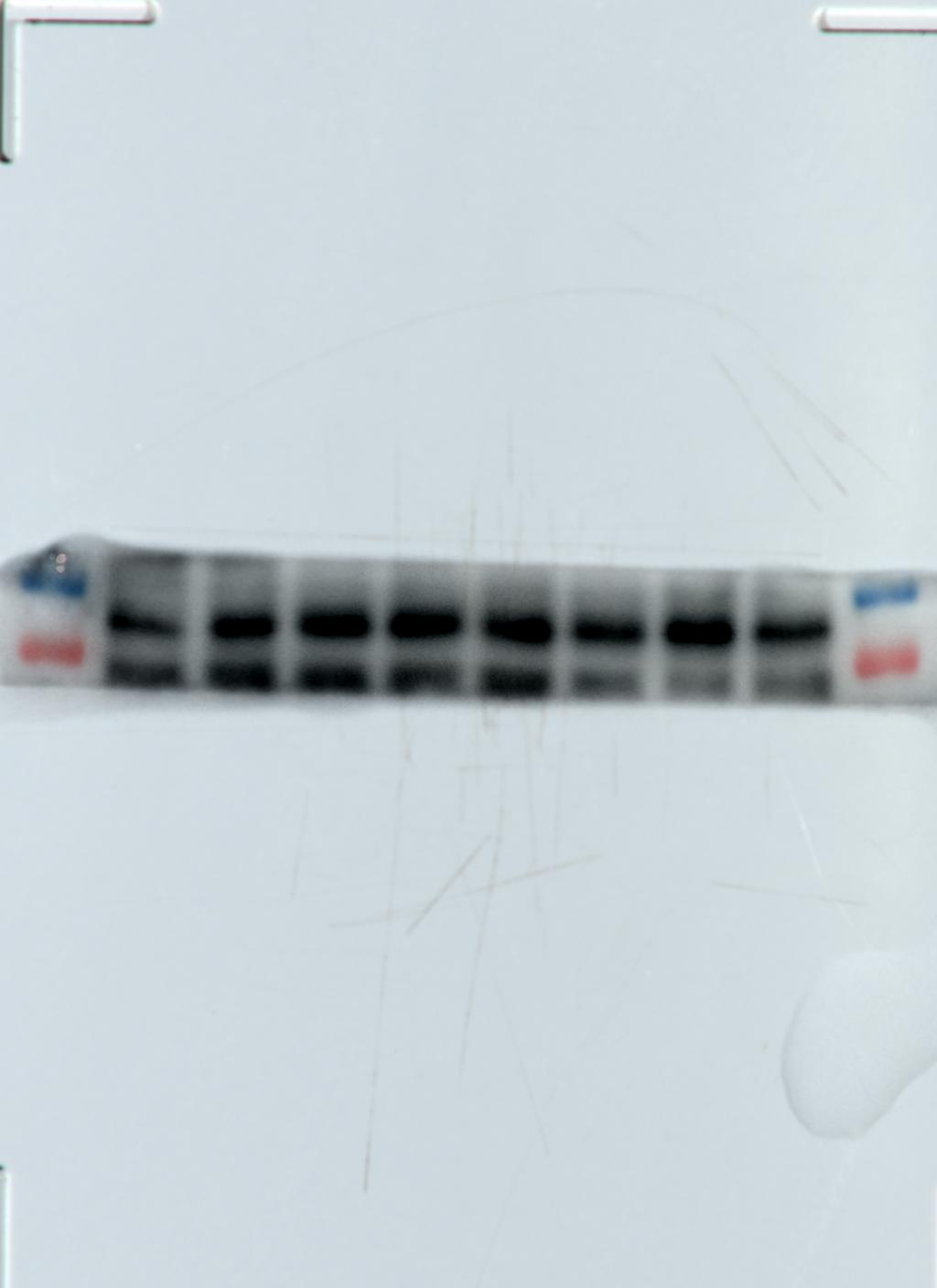


**pAKT**

**
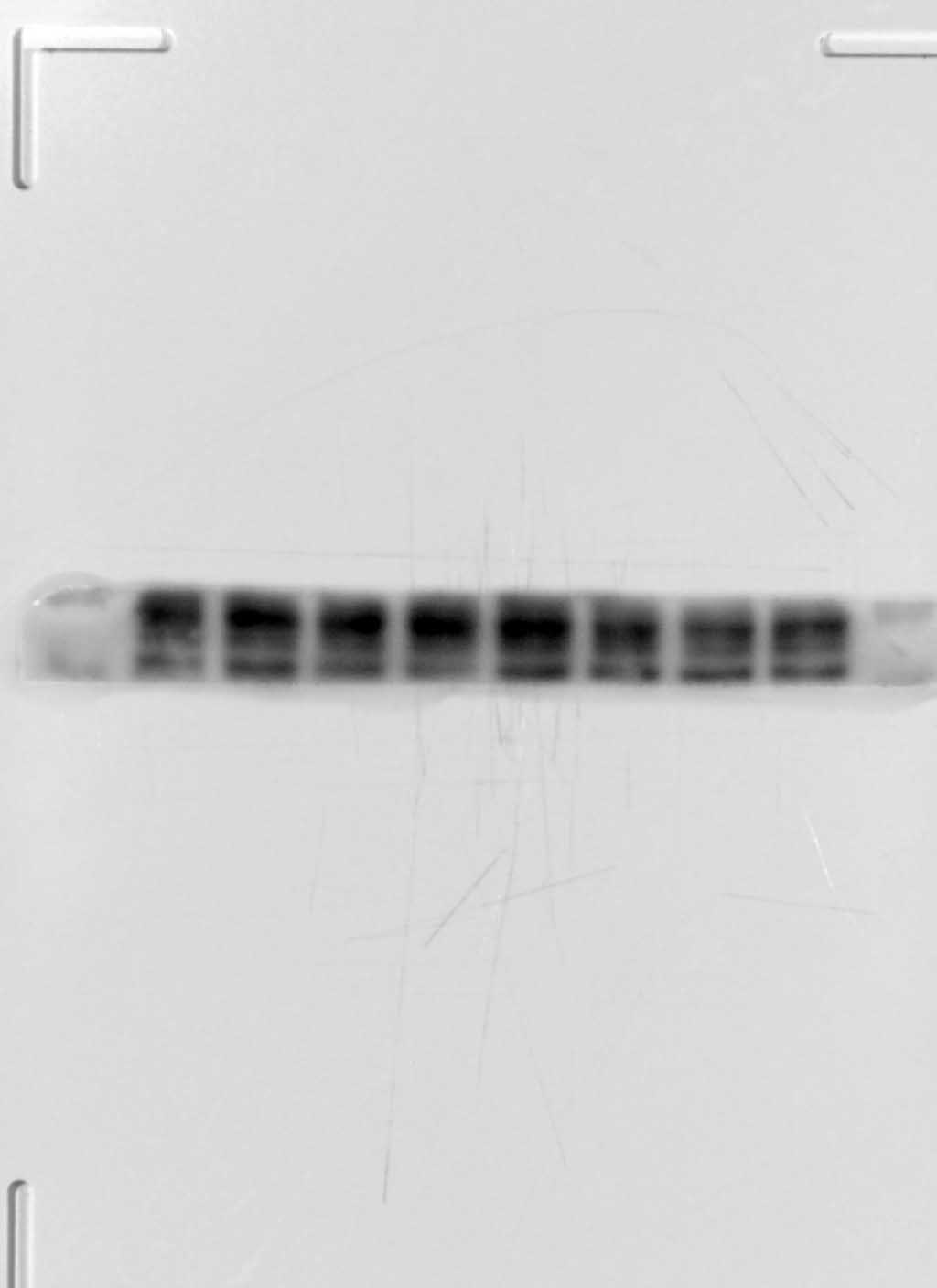
**

**AKT**


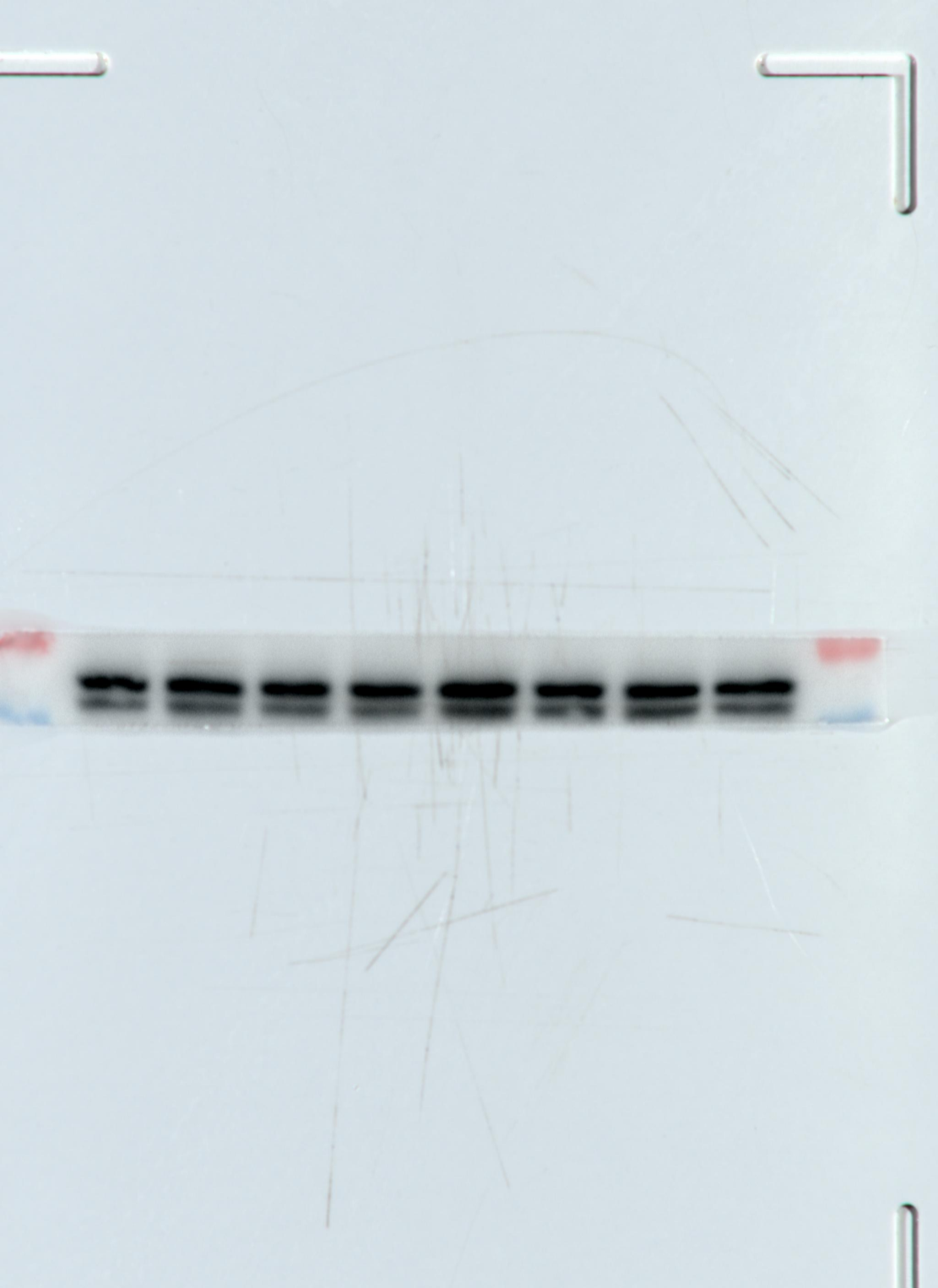


**β-ACTIN**


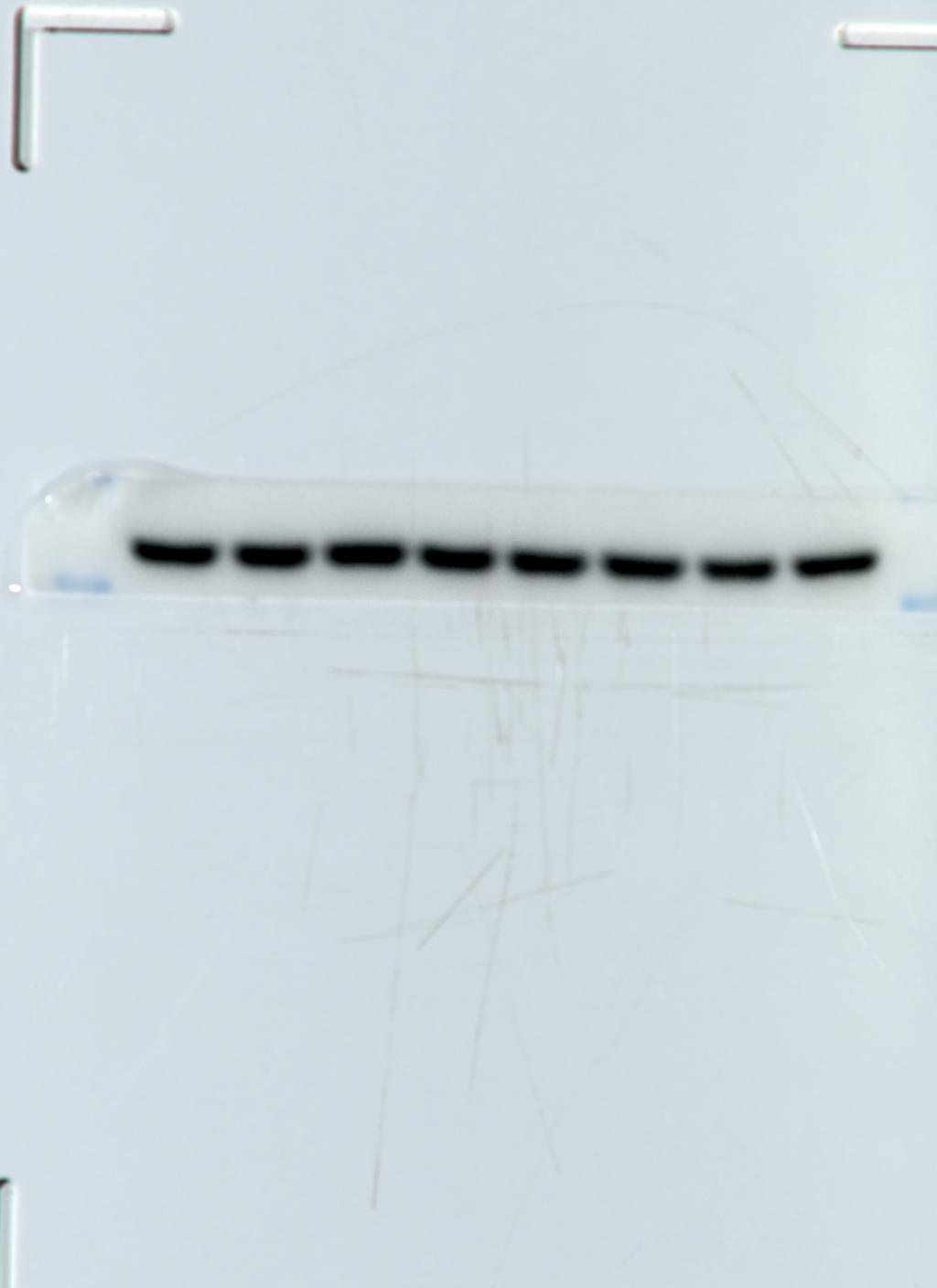


**Fig 5C**

**pYAP1**


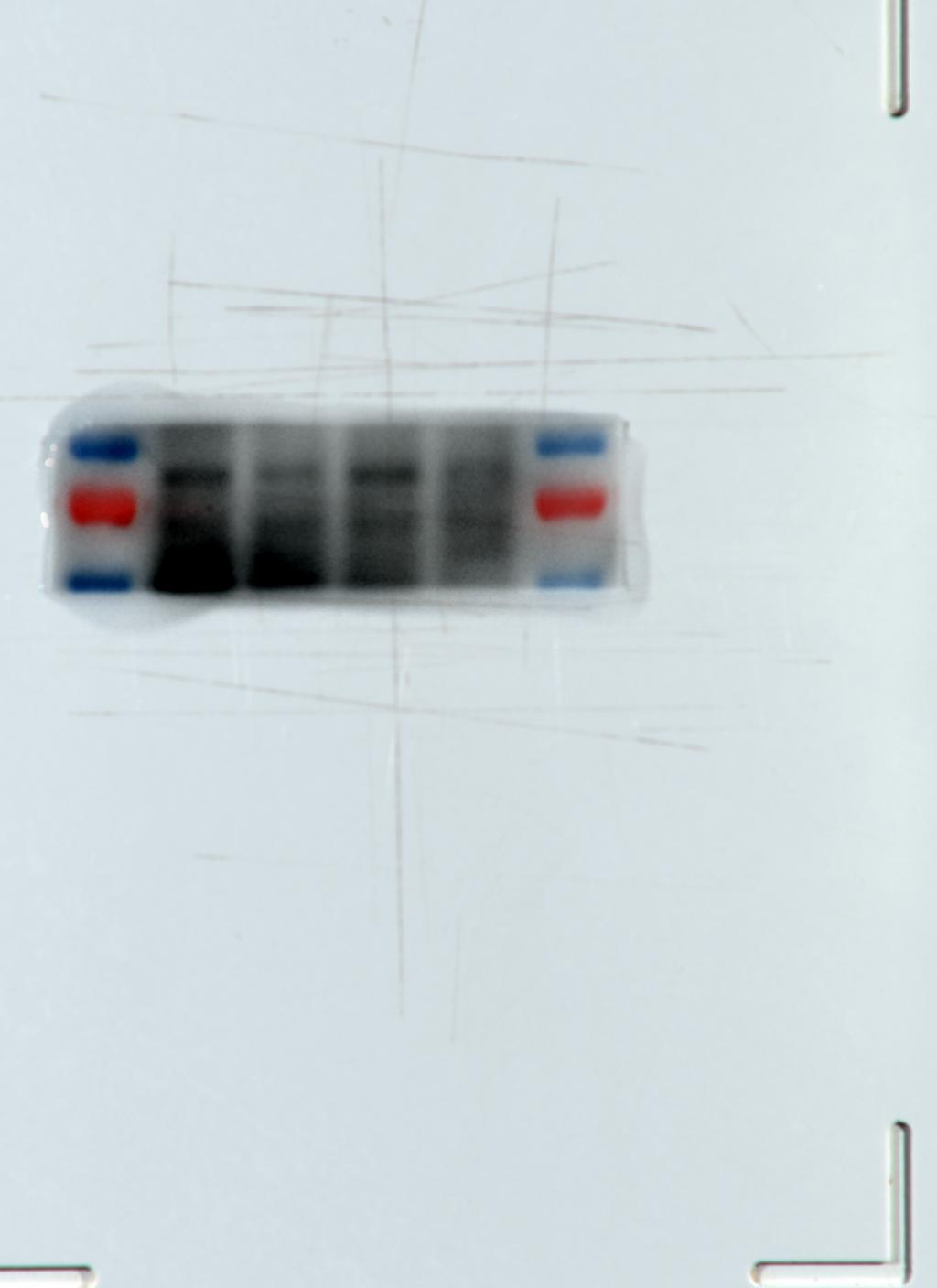


**YAP1**


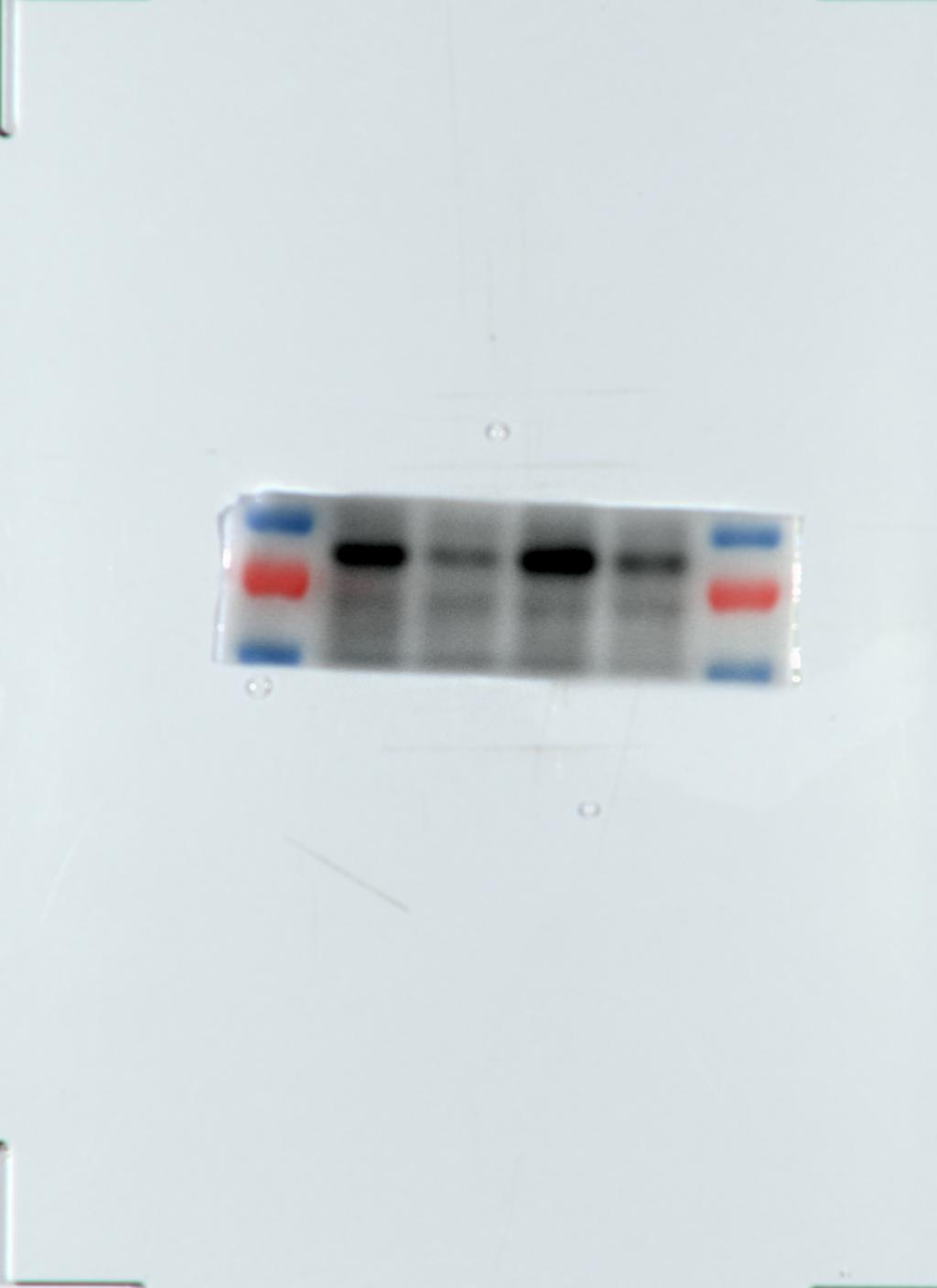


**pAKT**

**
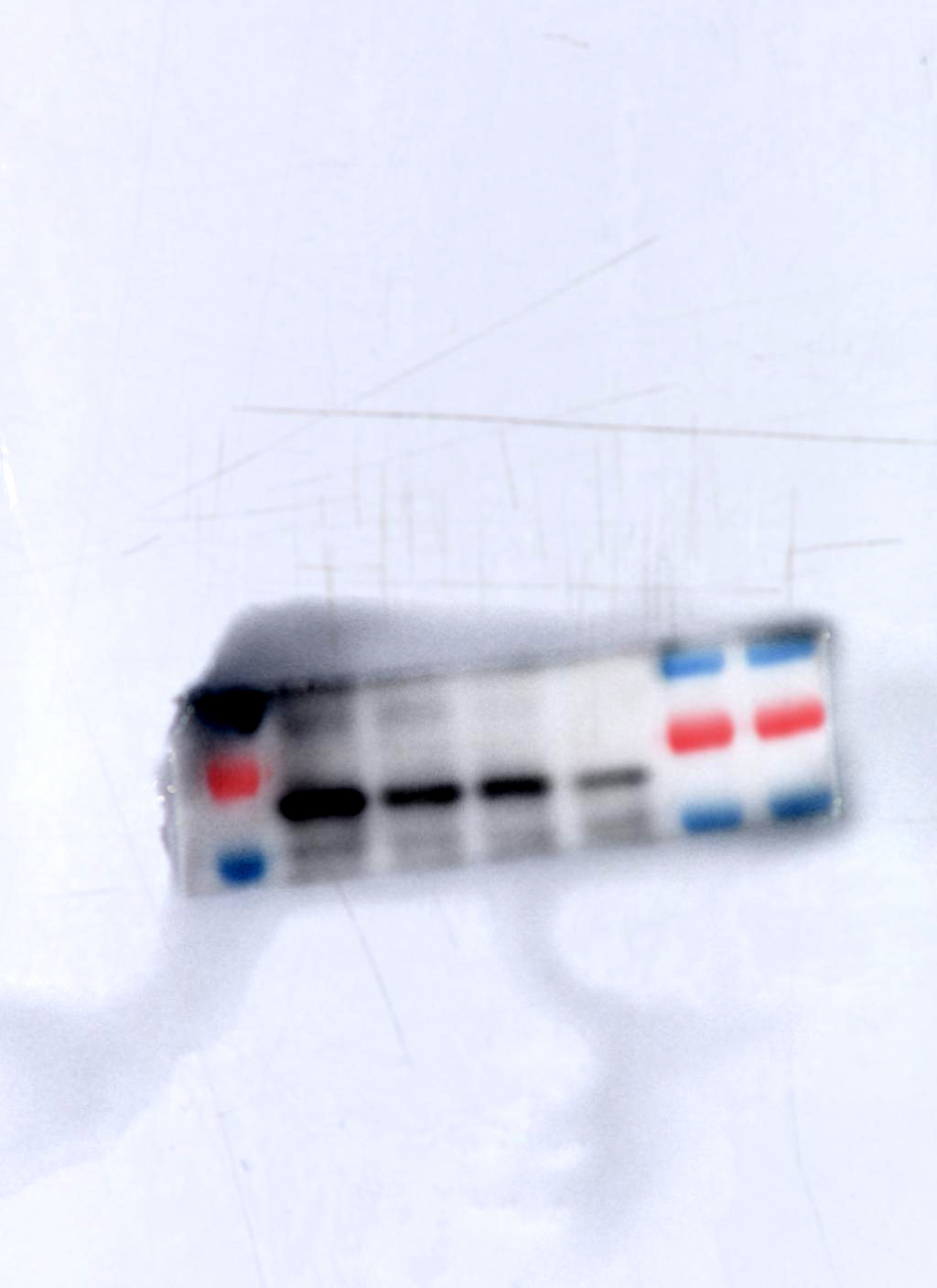
**

**AKT**


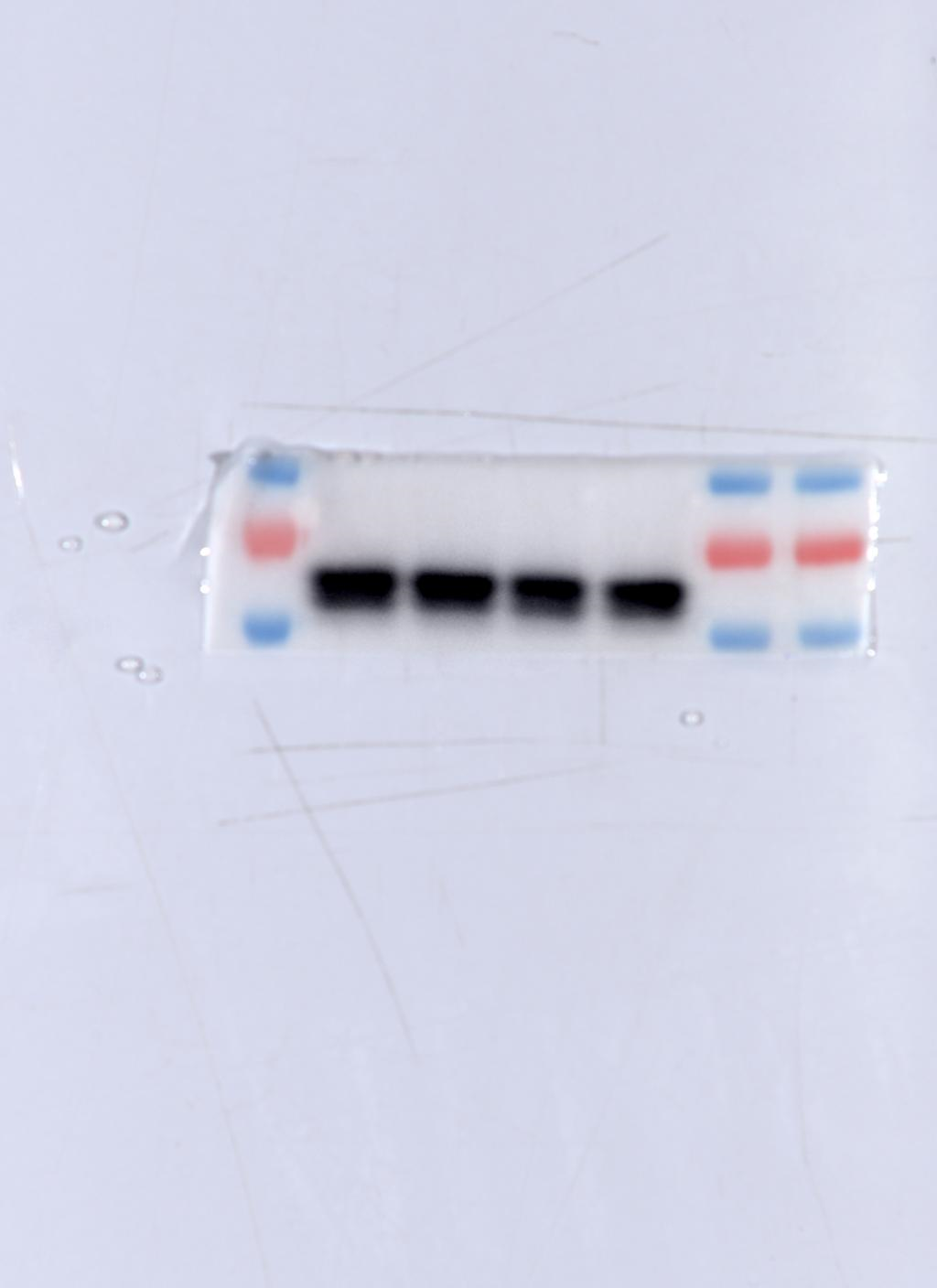


**PCNA**


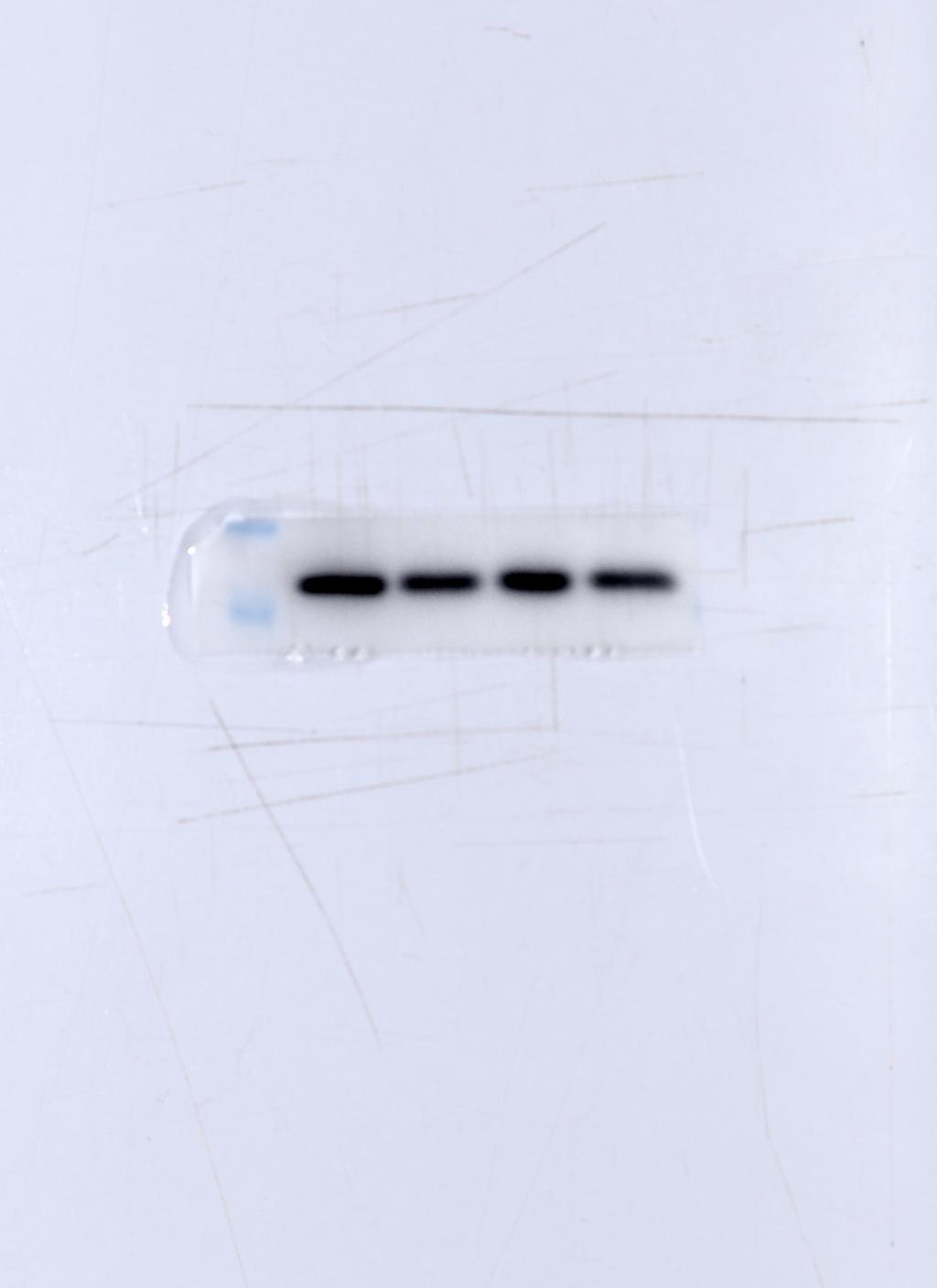


**GAPDH**


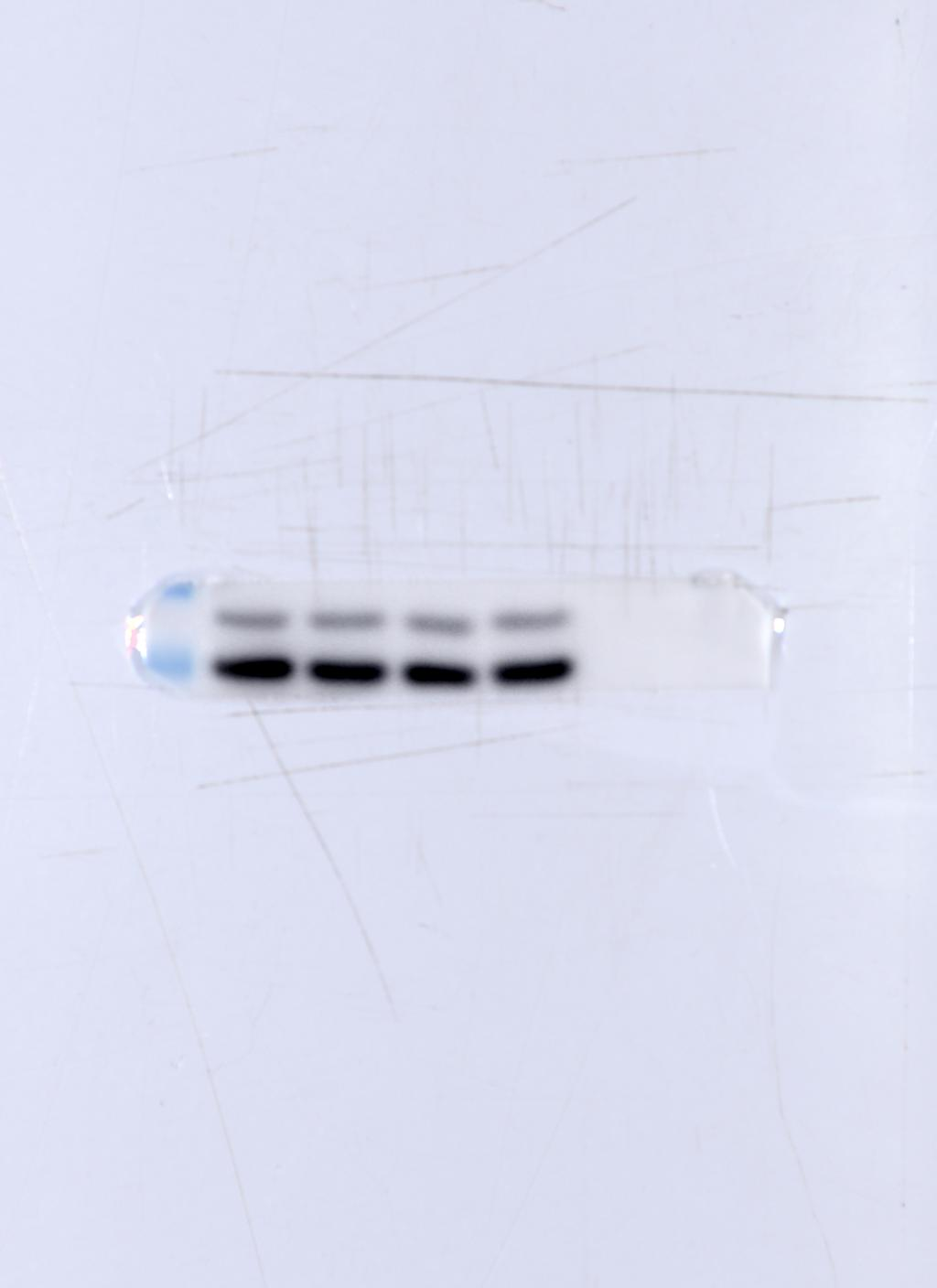


**Fig6A**

**pYAP1**

**
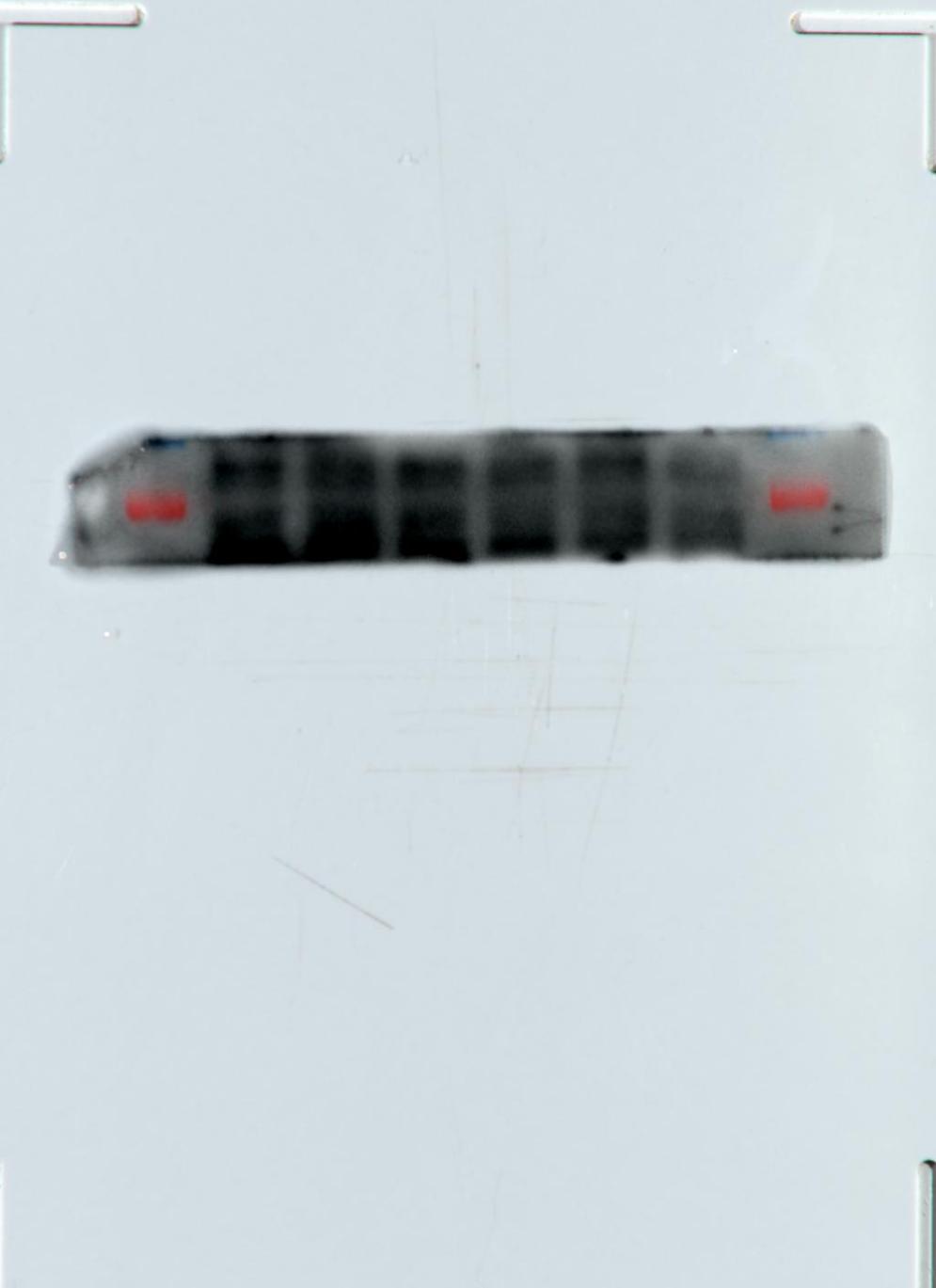
**

**YAP1**


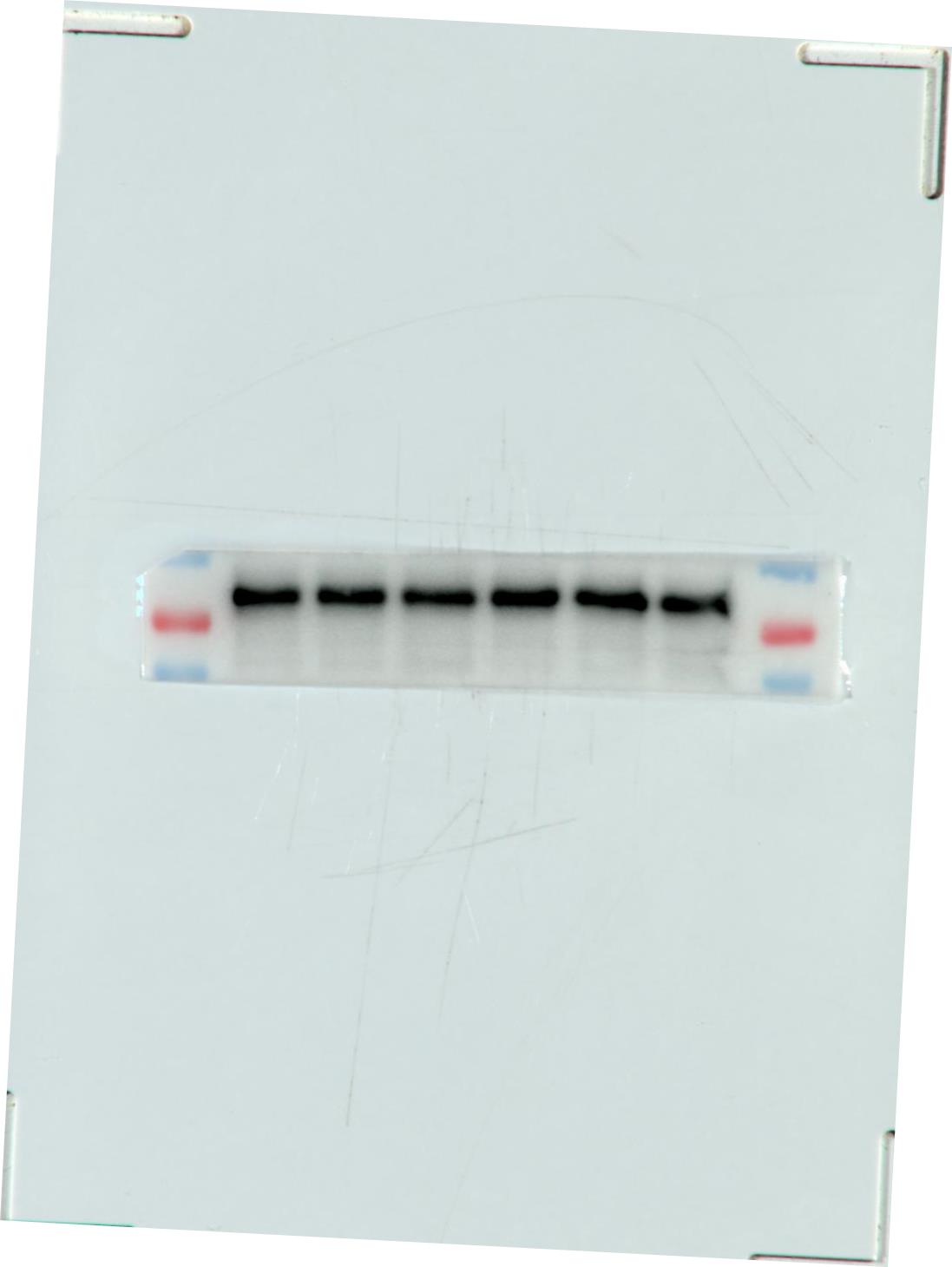


**pAKT**


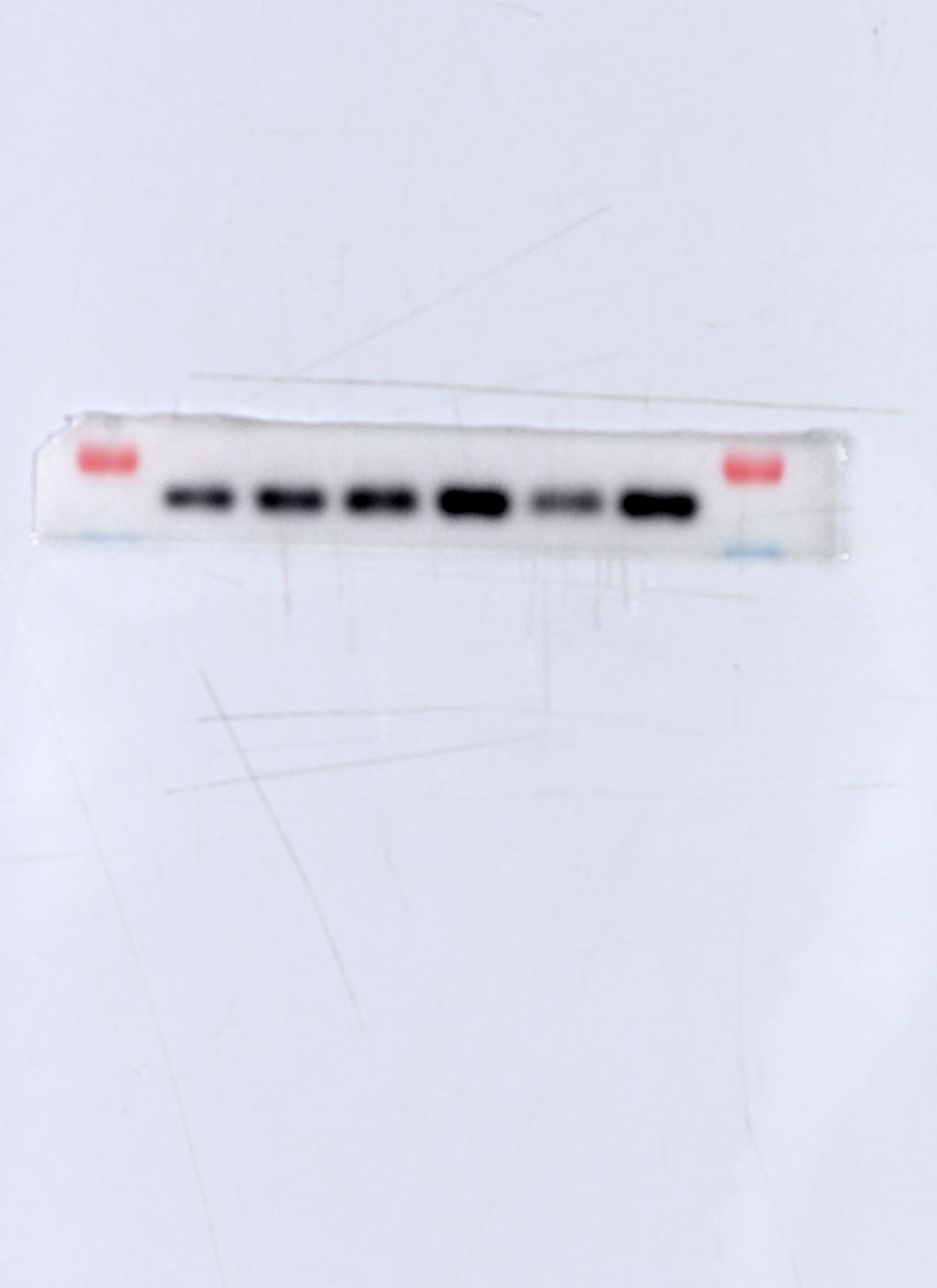


**AKT**


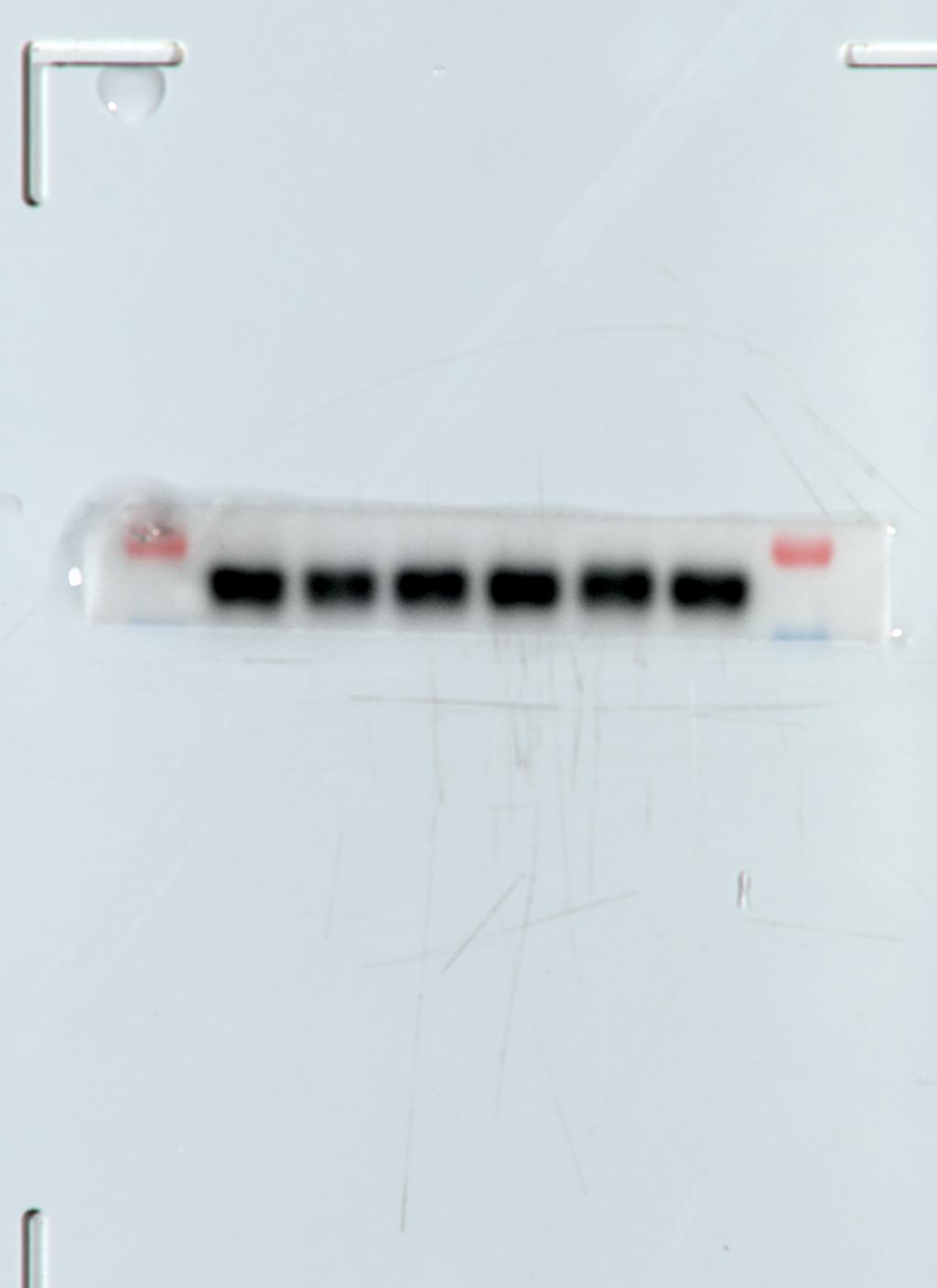


**ACTIN**


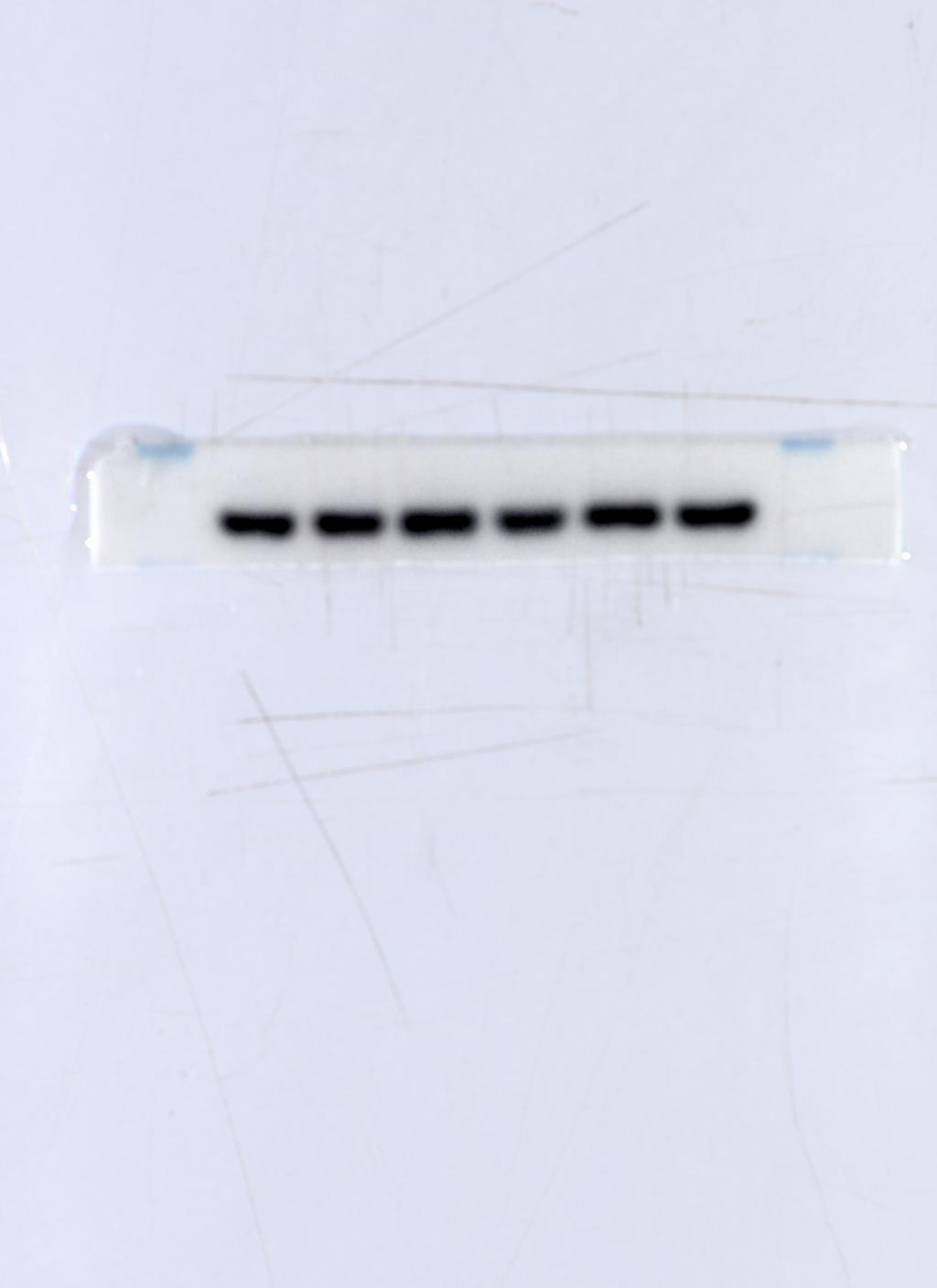


**FigS2E**

**pYAP1**


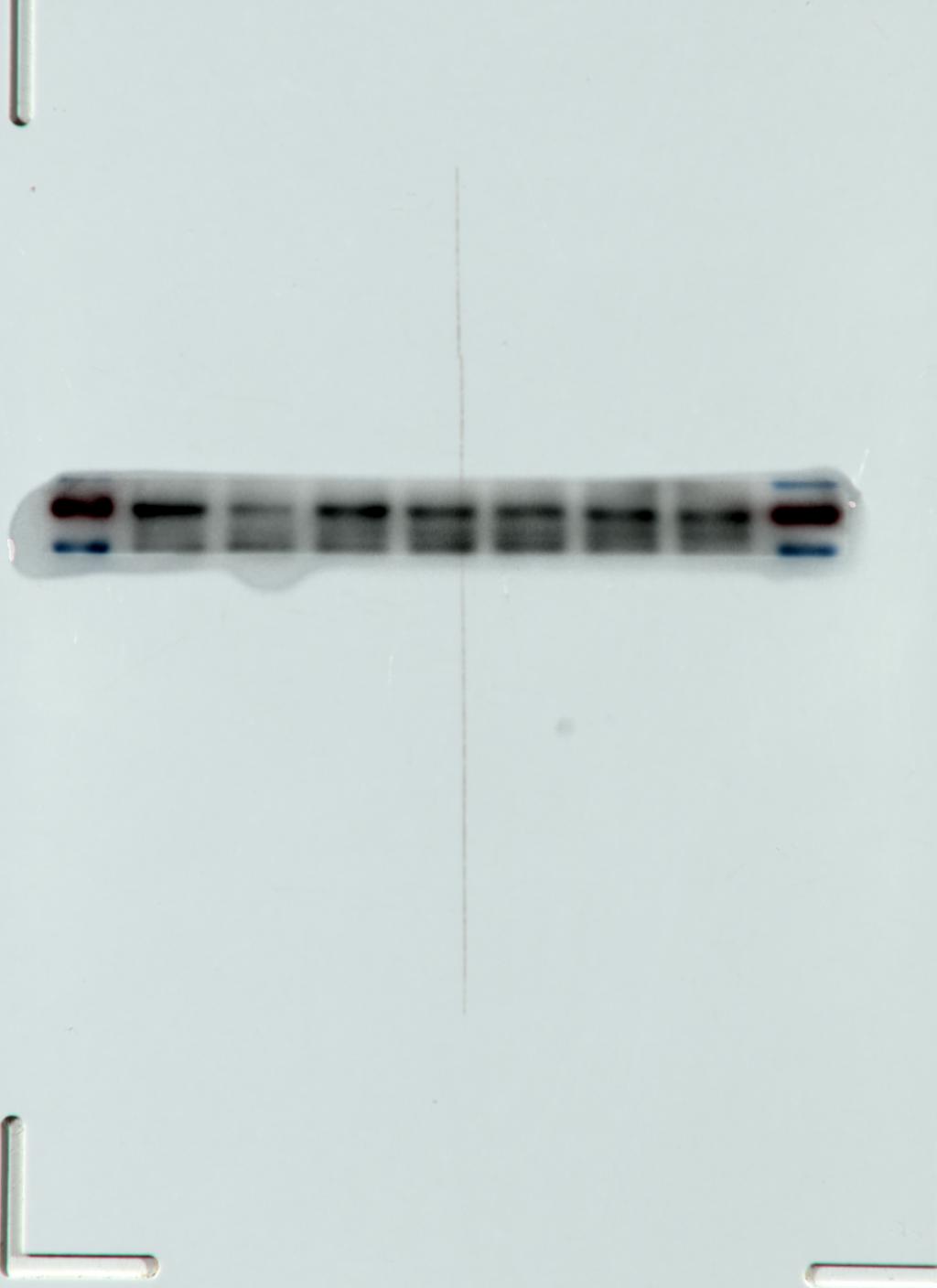


**YAP1**

**
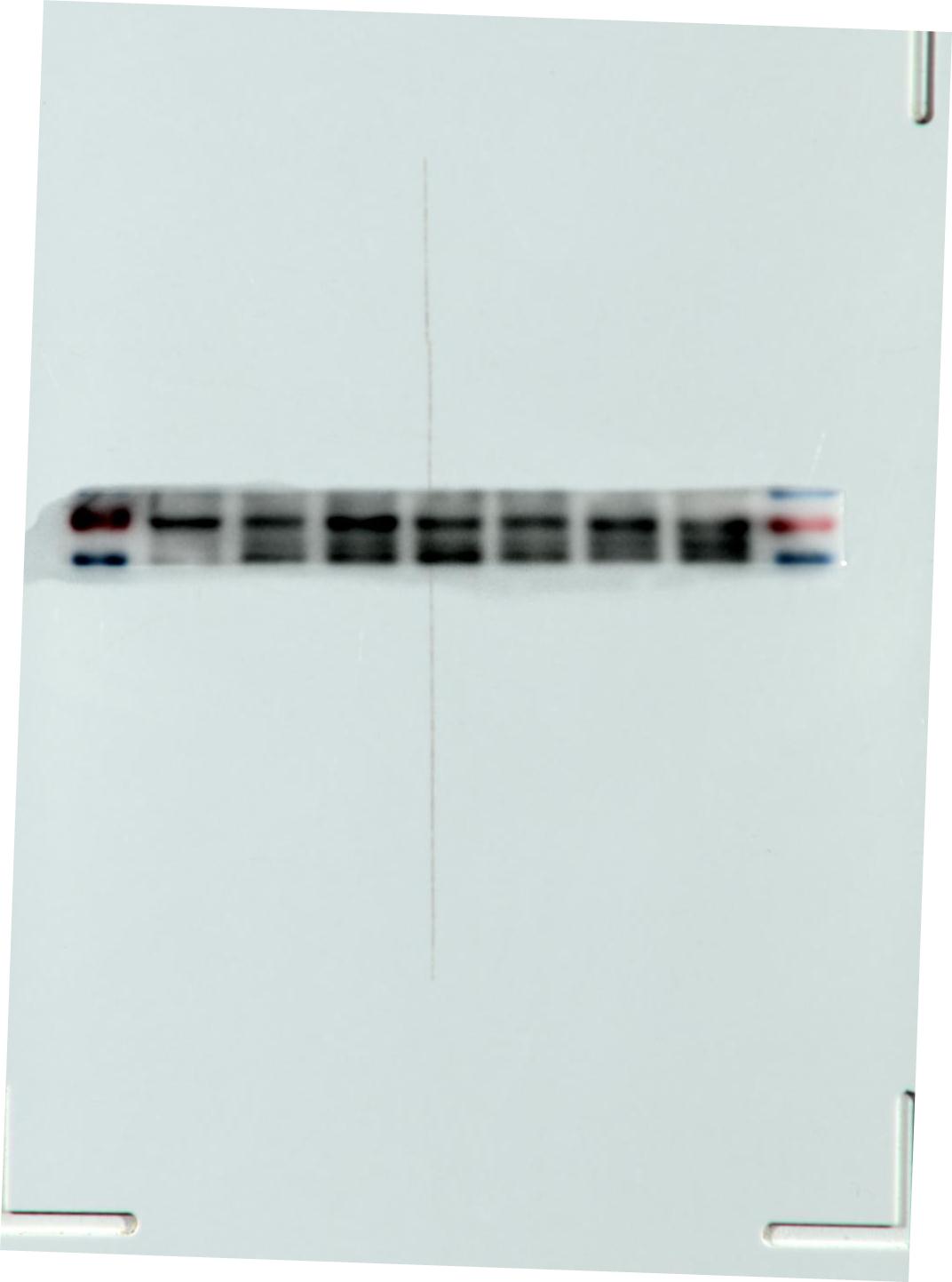
**

**ACTIN**

**
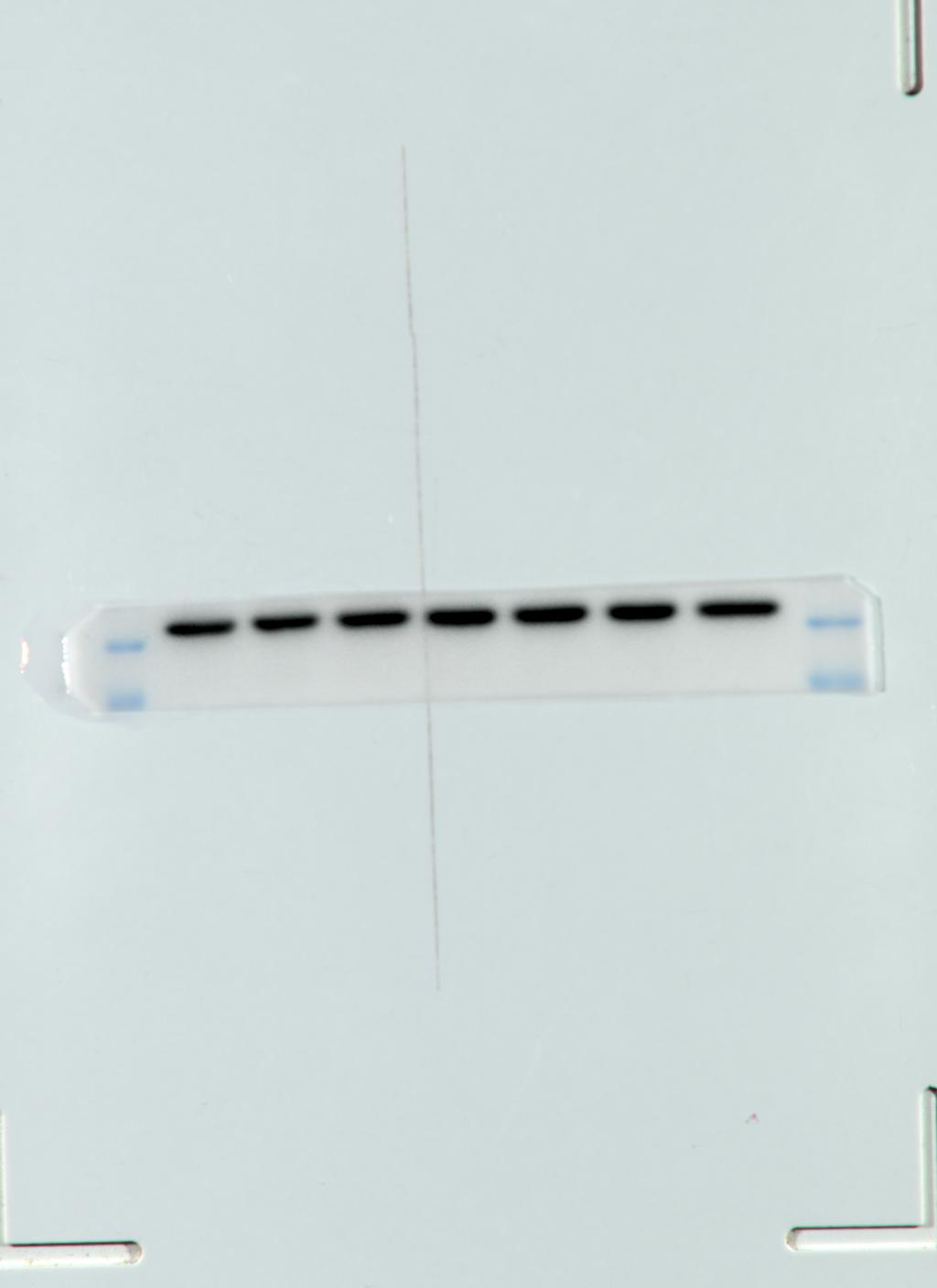
**

**FigS4E**

**MIGA2**


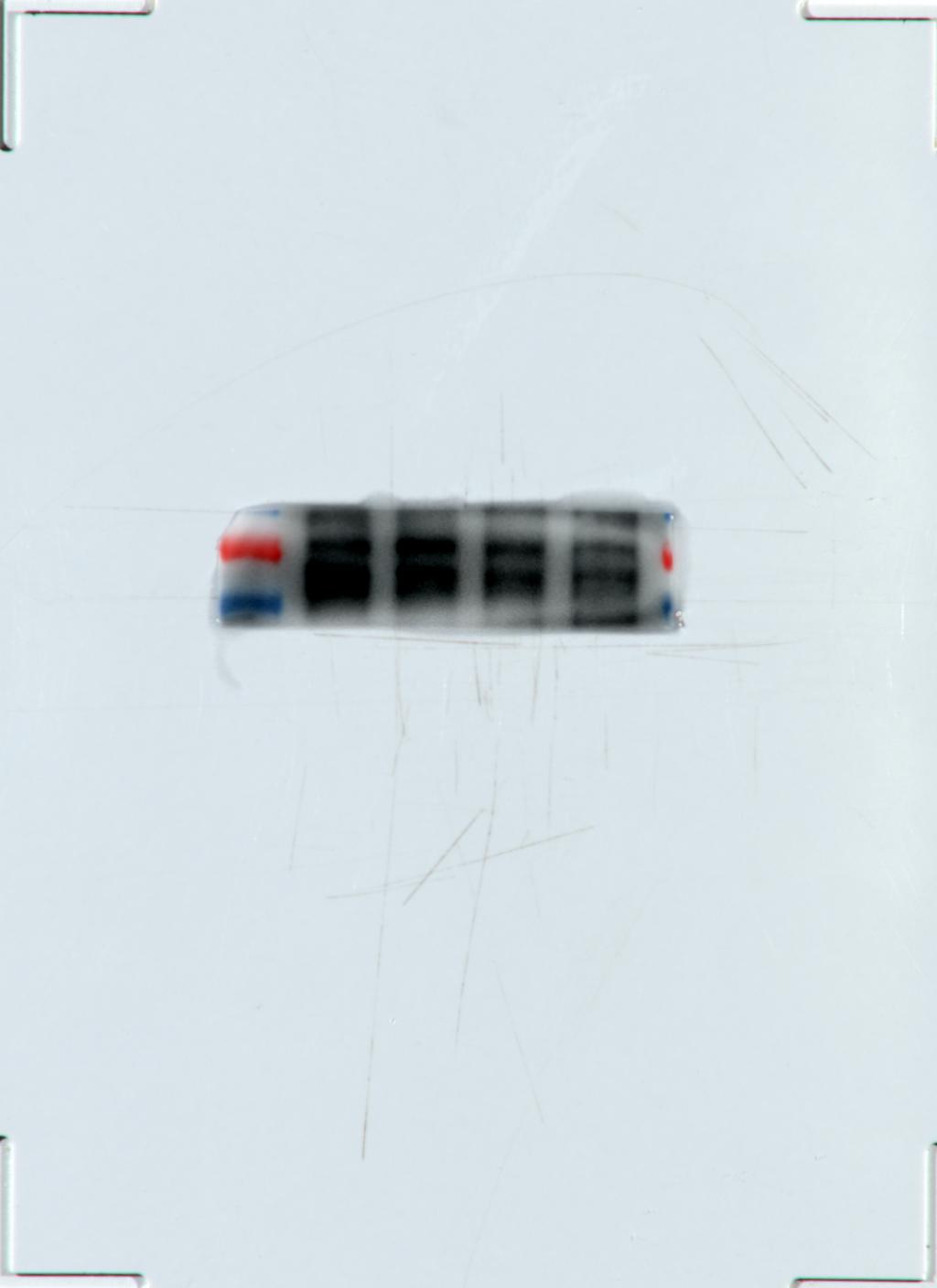


**MFN2**


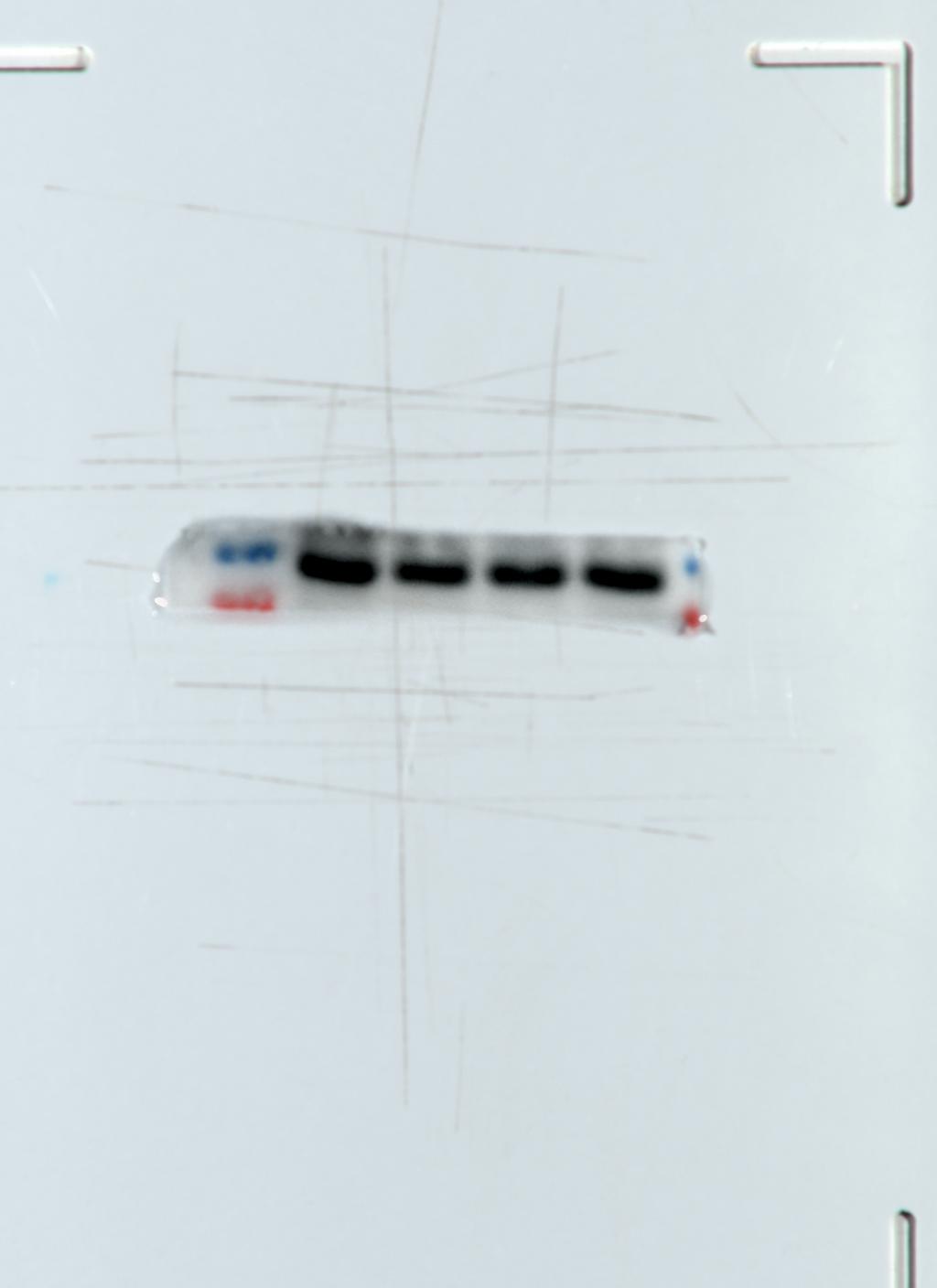


**ACTIN**


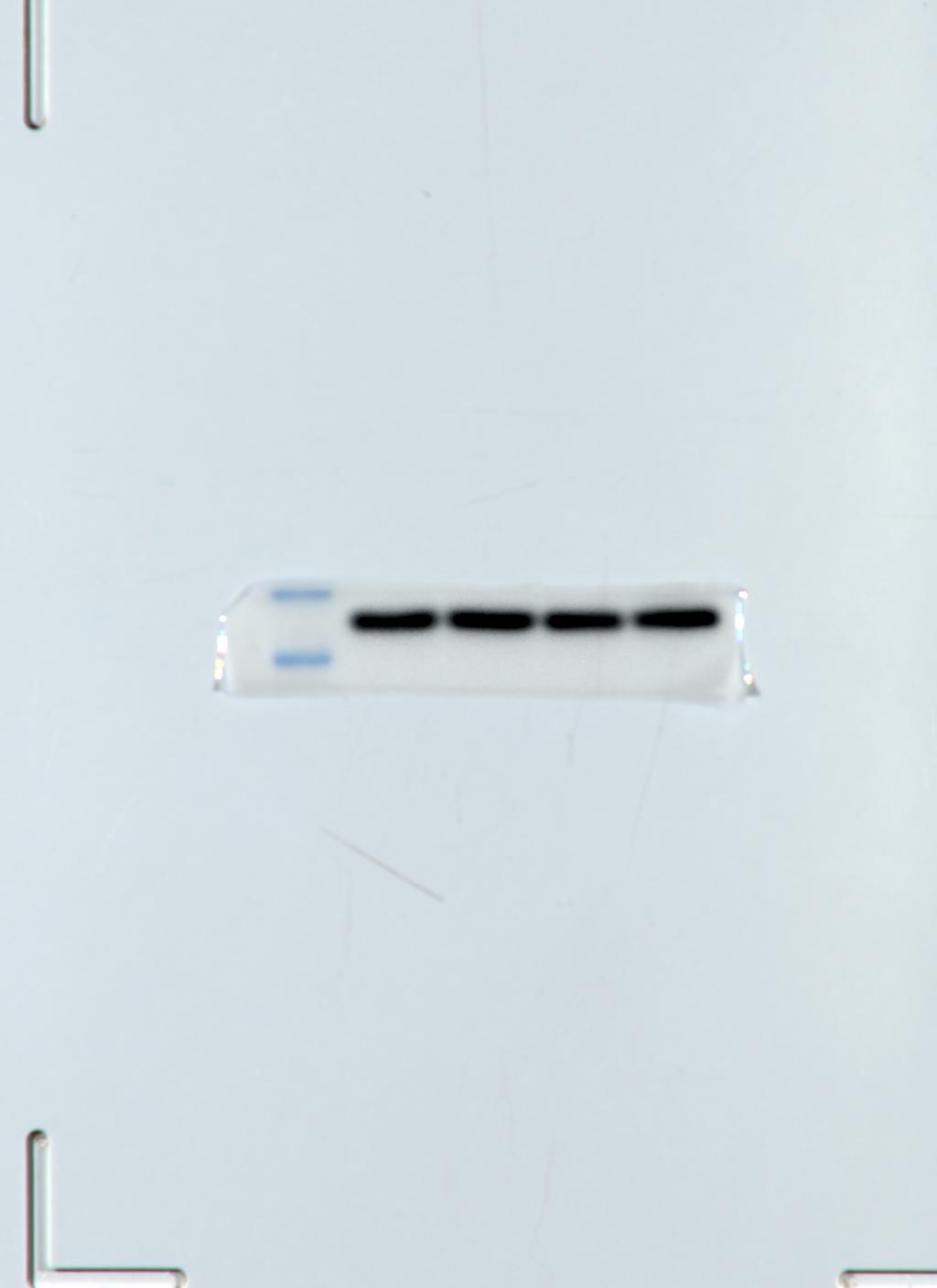


**FigS5B**

**MIGA2**


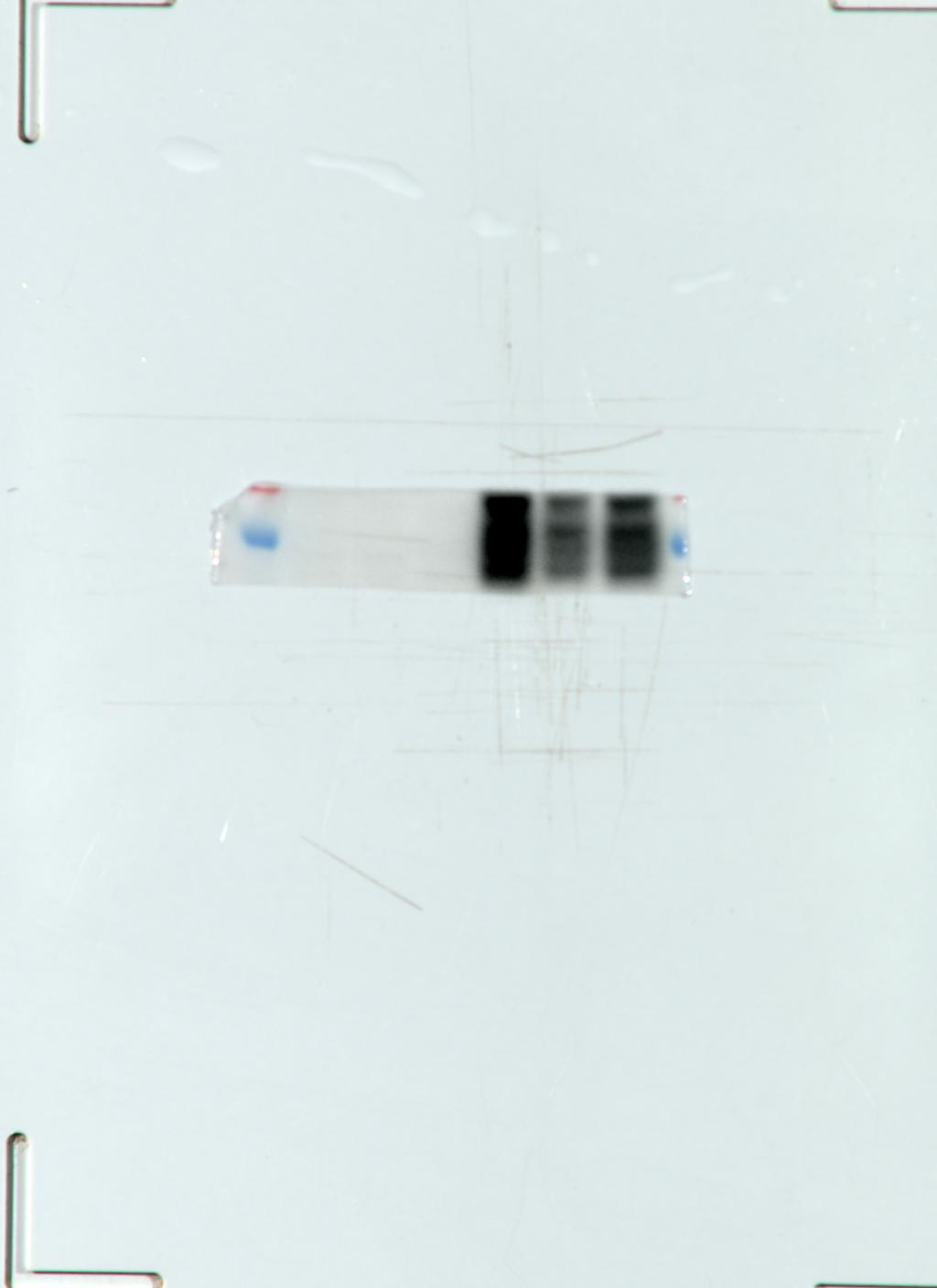


**HA**

**
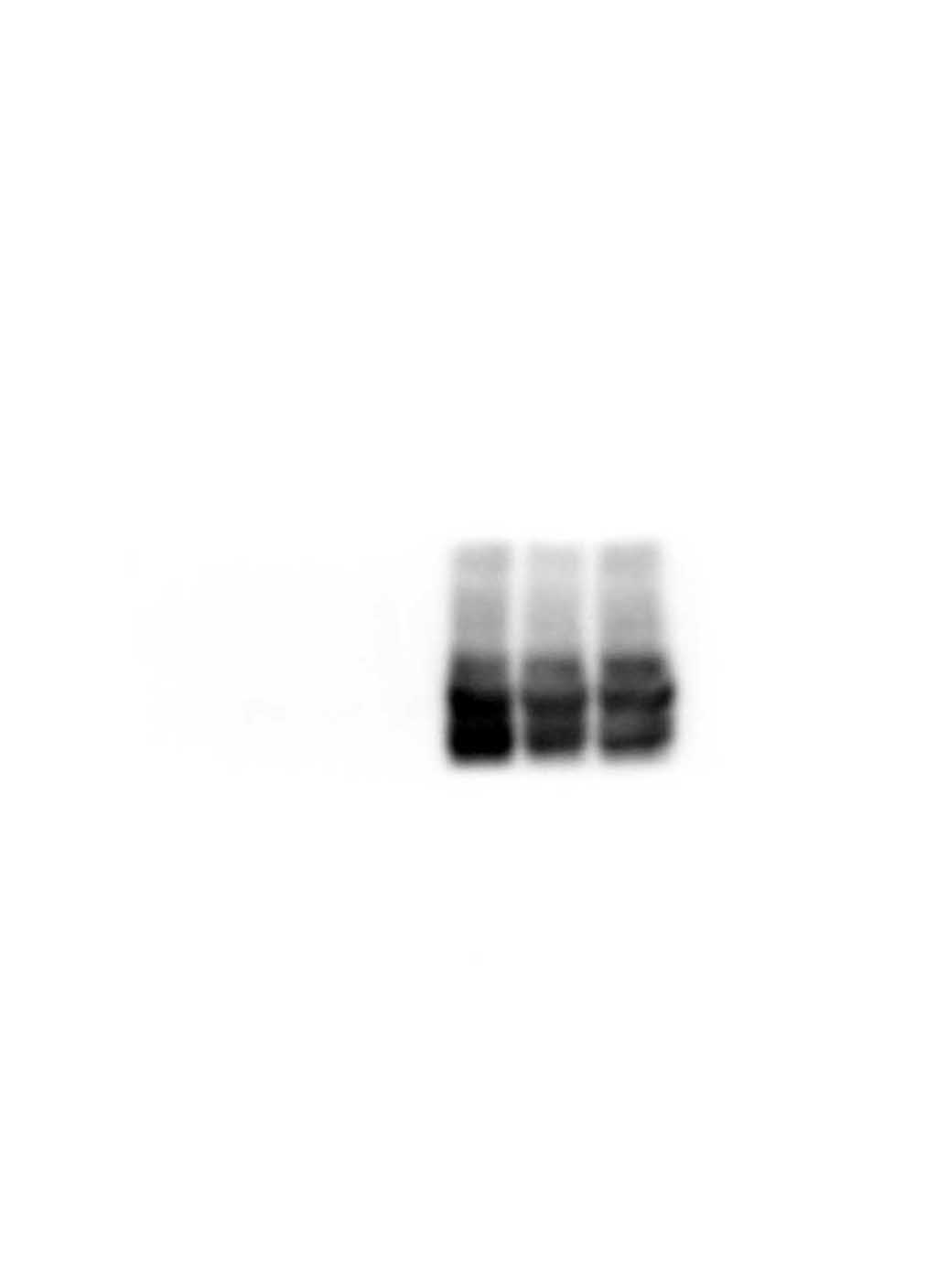
**

**GAPDH**


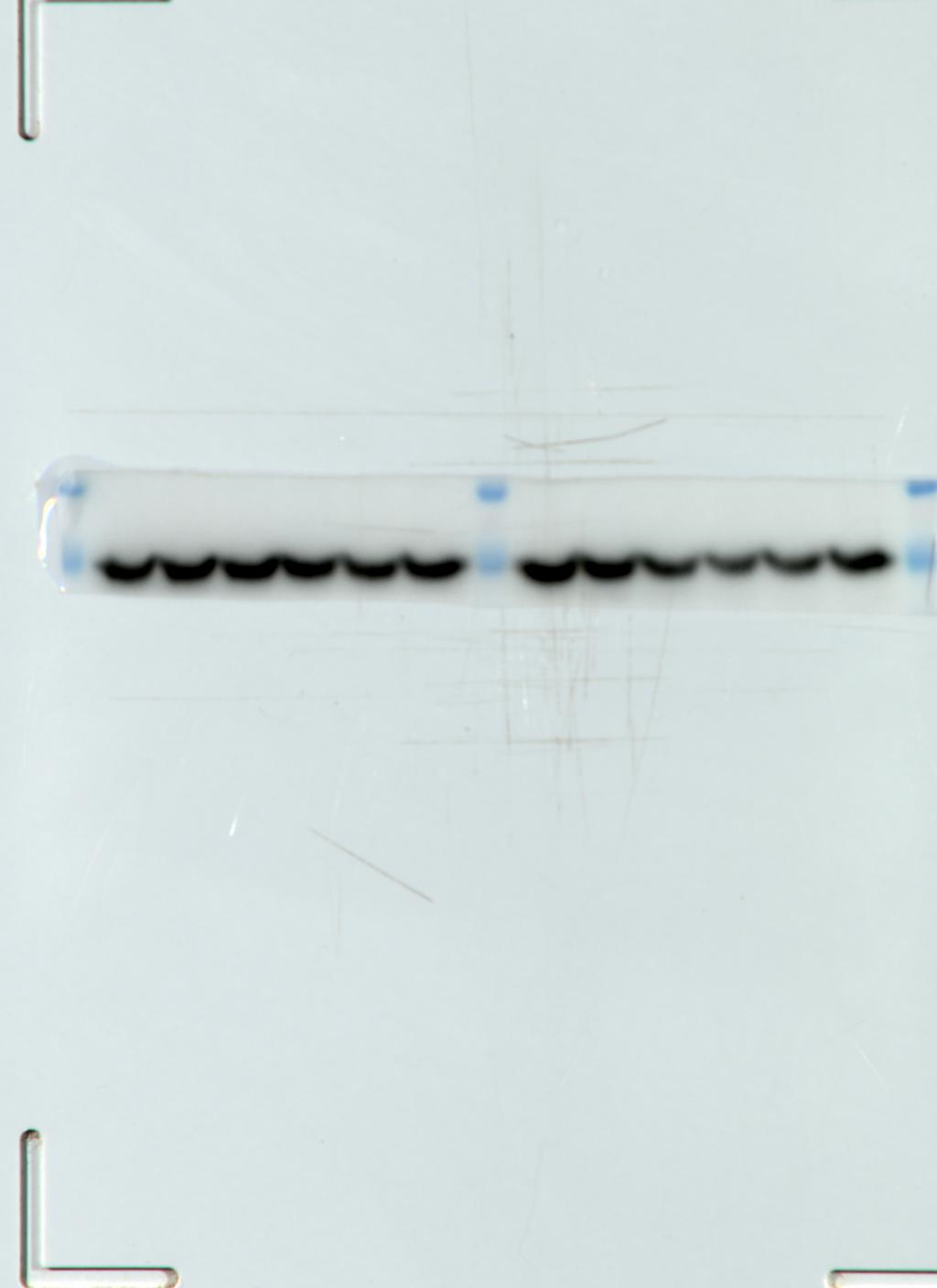


**FigS5C**

**MIGA2**


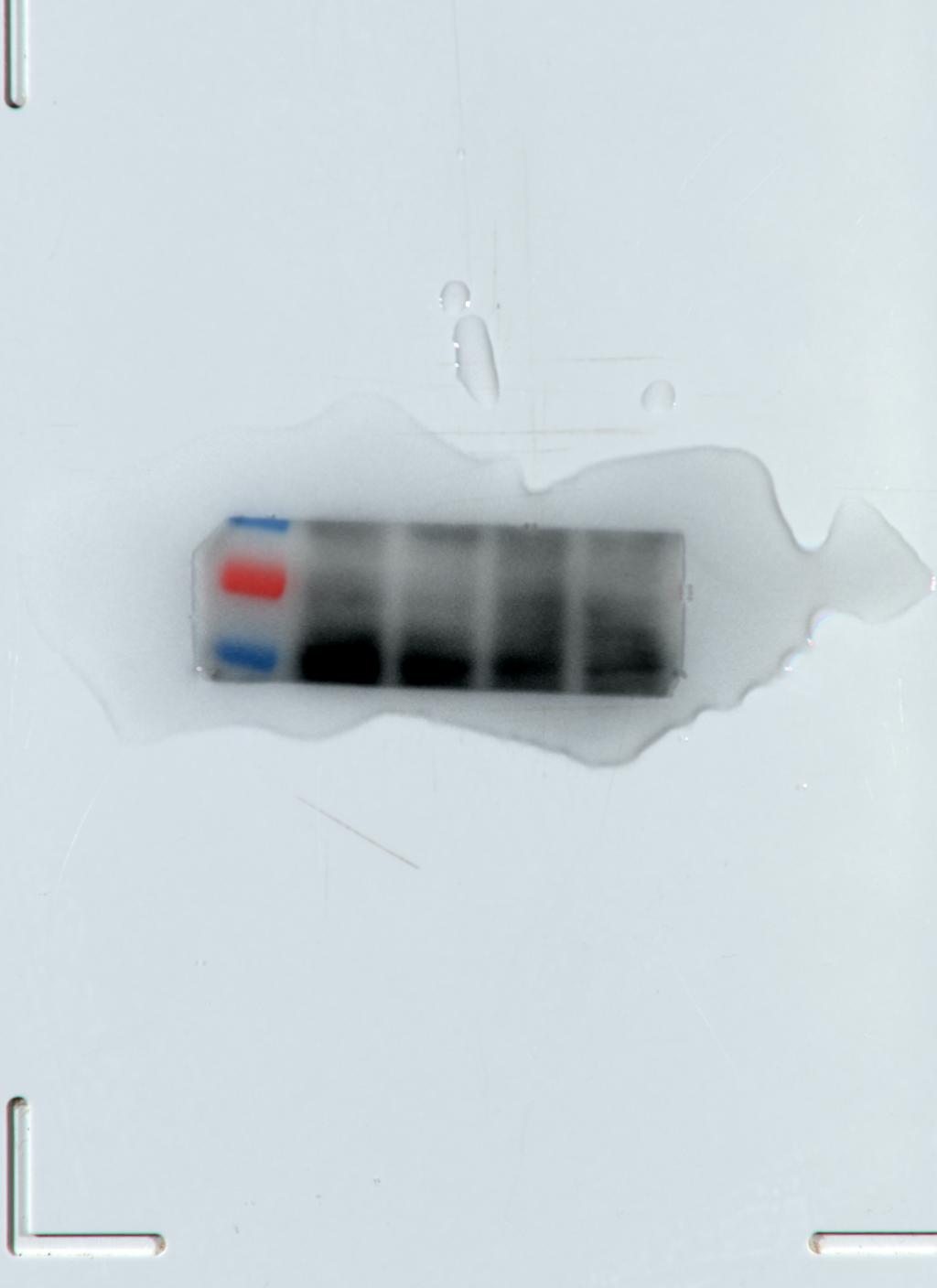


**ACTIN**


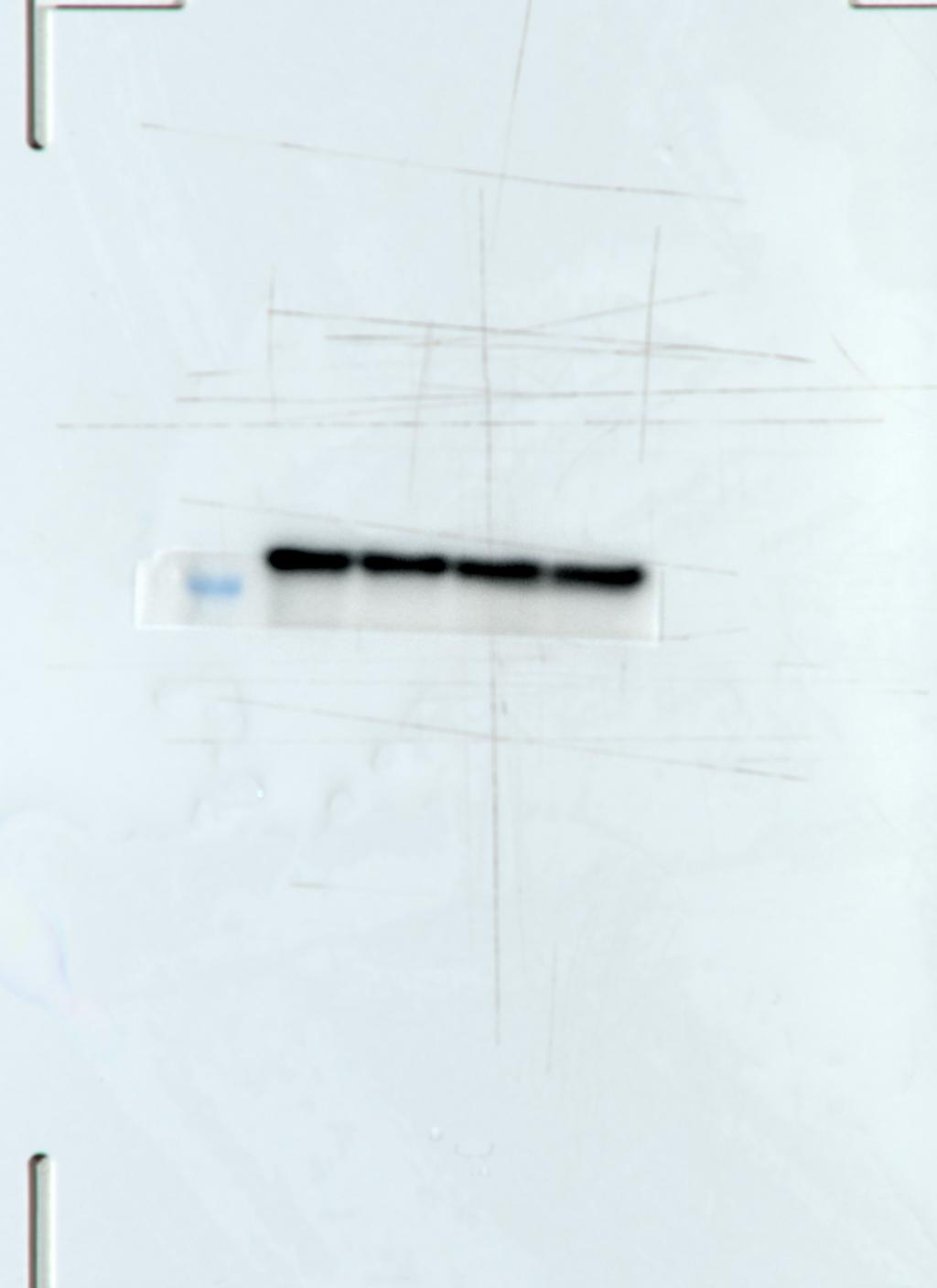

Supplement: Supplementary file 3 — Original Data File [file 41419_2023_6312_MOESM3_ESM.docx]
